# Genome-wide association study of the human brain functional connectome reveals strong vascular component underlying global network efficiency

**DOI:** 10.1038/s41598-022-19106-7

**Published:** 2022-09-02

**Authors:** Steven Bell, Daniel J. Tozer, Hugh S. Markus

**Affiliations:** Stroke Research Group, Department of Clinical Neurosciences, University of Cambridge, Cambridge, UK

**Keywords:** Neuroscience, Genetics of the nervous system, Neuro-vascular interactions

## Abstract

Complex brain networks play a central role in integrating activity across the human brain, and such networks can be identified in the absence of any external stimulus. We performed 10 genome-wide association studies of resting state network measures of intrinsic brain activity in up to 36,150 participants of European ancestry in the UK Biobank. We found that the heritability of global network efficiency was largely explained by blood oxygen level-dependent (BOLD) resting state fluctuation amplitudes (RSFA), which are thought to reflect the vascular component of the BOLD signal. RSFA itself had a significant genetic component and we identified 24 genomic loci associated with RSFA, 157 genes whose predicted expression correlated with it, and 3 proteins in the dorsolateral prefrontal cortex and 4 in plasma. We observed correlations with cardiovascular traits, and single-cell RNA specificity analyses revealed enrichment of vascular related cells. Our analyses also revealed a potential role of lipid transport, store-operated calcium channel activity, and inositol 1,4,5-trisphosphate binding in resting-state BOLD fluctuations. We conclude that that the heritability of global network efficiency is largely explained by the vascular component of the BOLD response as ascertained by RSFA, which itself has a significant genetic component.

## Introduction

Complex brain networks play a central role in integrating activity across the human brain^1,2^. Many of these have been studied in response to specific tasks, but the human brain remains active in the absence of any explicit stimuli^3^. Such resting state functional networks, or spontaneous intrinsic brain activity, can be studied utilising changes in blood oxygen level-dependent (BOLD) MRI signal, and analysing temporal correlations of signal change indicating connectivity between regions of grey matter. Resting state functional magnetic resonance imaging (rs-fMRI) has led to the discovery of multiple resting state networks including the default mode, central executive (ie. fronto-parietal), salience, attention, somatosensory and visual networks^4,5^. Furthermore, using graph theory methods measures of global integration across the resting brain can be derived^6^. These include ‘global efficiency’ which reflects the ability of the brain to integrate signals in parallel over the entire inter-component network, and ‘local efficiency’ which is an indicator of segregation in reflecting the average ability to propagate signals to immediate neighbouring areas of the brain. Increasing evidence has associated disruption in these networks with a variety of neurological and psychiatric disorders, including dementia^7–9^. Therefore better understanding of the mechanisms underlying these resting state brain networks is important.

Twin and family studies have reported a low to moderate degree of genetic contribution to intrinsic brain activity (e.g. 23–30% for global efficiency)^10^. Candidate gene studies have identified underlying genetic associations including *APOE* and *KIBRA*^11^. More recently genome wide association studies (GWAS) have offered the opportunity to identify completely novel associations in a hypothesis free manner. Functional connectivity traits in rs-fMRI are typically noisier than structural MRI traits and sensitive to scanner and acquisition differences, and this can reduce the ability to detect associations^4^. However, recently very large datasets performed using identical scanner and acquisition characteristics such as UK Biobank have become available. Such GWAS studies have identified a number of novel associations with measures related to resting state connectivity and shown genetic correlations with a variety of neurological and psychiatric disorders^12–14^.

It is often assumed that such functional networks primarily provide information about ‘brain function’ and reflect neuronal activity. However the BOLD signal from which they are derived depends upon vasoneuronal coupling^15^ and thus can be influenced by neuronal and vascular factors^16^. Therefore genetic influences on both the vascular and neuronal components of the signal could underlie the heritability of functional brain networks.

To investigate this further we utilised over 36,000 scans from UK Biobank. We performed a GWAS on both global and local efficiency, and also individual resting state networks. Additionally we performed a GWAS for BOLD resting state fluctuation amplitudes (RSFA) which are thought to reflect the vascular component of the BOLD signal^17^. We further adjusted for this variable in our GWAS analyses of functional networks to determine how much of any association was accounted for by this. We also performed transcriptome- and proteome-wide association studies to identify loci that influence network efficiency and resting state networks through their effects on gene-expression and protein abundance to provide further insights into biology of these complex traits.

Our analyses revealed that global efficiency was the most heritable of the functional network variables. However, this heritability was largely explained by the RSFA. We therefore applied several bioinformatic techniques to gain insights into the underlying biological mechanisms responsible for the vascular component of the BOLD signal.

## Results

### Genetic discoveries

We performed a GWAS of 17,546,374 genetic variants for our 10 network outcomes (graph-based metrics of global and local network efficiency, plus the default mode, medial frontal, fronto-pariental, subcortical-cerebellum, motor, visual association, visual 1 and visual 2 resting state subnetworks) in up to 36,150 participants of European ancestry in the UK Biobank. An overview of our findings is presented in Table 1 with further information for each analysis shown in Supplementary Table 1. We observed little evidence of genomic inflation in any of our analyses (LD intercept ranging from 1.00 to 1.01). QQ plots for all GWAS are presented in Supplementary Figs. 1–12.

**Table 1:** Overview of results for each network phenotype analysed.

Phenotype	N	Genomic loci	LDSC intercept	h^2^ (SE)	TWAS loci	Brain PWAS	Plasma PWAS
Global efficiency	36,147	14	1.00	0.15 (0.03)	82	7	2
Local efficiency	36,148	1	1.00	0.02 (0.01)	0	0	2
Default-mode network	36,149	0	1.00	0.07 (0.02)	0	0	0
Frontoparietal network	36,150	0	1.00	0.05 (0.01)	2	1	0
Medial frontal	36,150	0	1.01	0.06 (0.02)	0	0	0
Motor network	36,150	2	1.00	0.08 (0.01)	0	0	0
Subcortical-cerebellum network	36,150	2	1.00	0.07 (0.01)	1	0	0
Visual association network	36,150	0	1.00	0.06 (0.01)	0	1	1
Visual network 1	36,150	3	1.00	0.07 (0.01)	8	1	0
Visual network 2	36,150	0	1.00	0.04 (0.01)	0	0	0
Average global resting state fluctuation amplitude (RSFA)	36,143	24	1.03	0.23 (0.03)	157	3	4
Global efficiency adjusted for RSFA	36,143	3	0.99	0.08 (0.01)	14	0	0

N, number of individuals included in the analysis; LDSC, linkage disequilibrium score regression; h^2^, heritability; SE, standard error; TWAS, transcriptome -wide association study; PWAS, proteome-wide association study.

We calculated SNP-based heritability (h^2^) for each phenotype (Table 1). Global efficiency had the highest heritability at 0.15 (standard error [SE] = 0.03), while all other measures had heritability estimates less than 0.08, with local efficiency displaying the lowest (h^2^ 0.02, SE 0.01).

**Figure 1:**
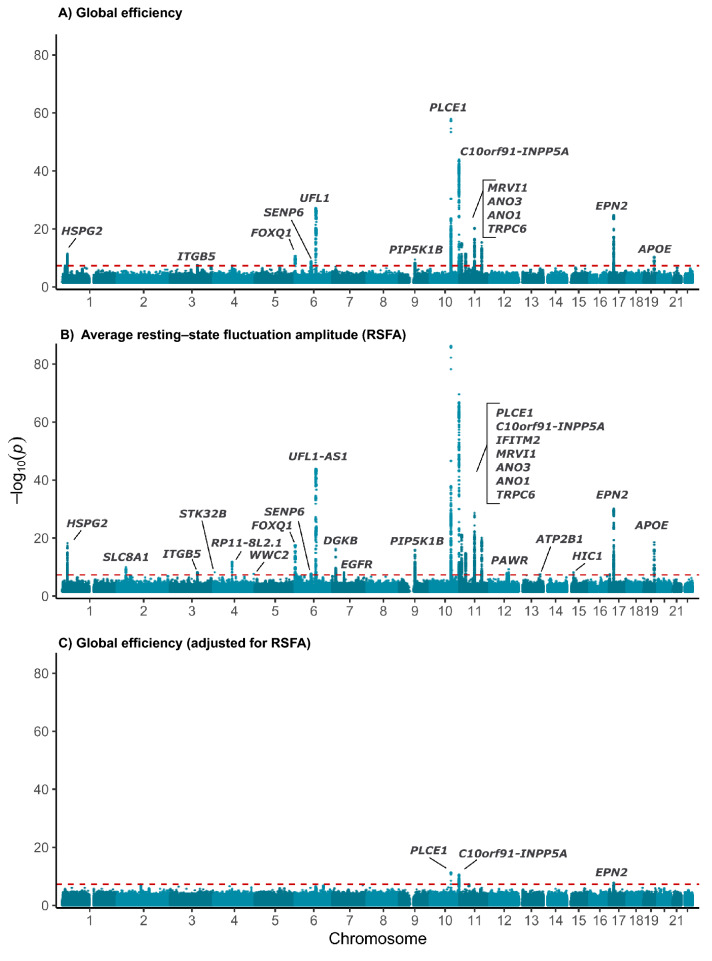
Manhattan plots for GWAS of a (**A**) global efficiency, (**B**) average resting state fluctuation amplitudes (RSFA), and (**C**) global network efficiency adjusted for RSFAPositional mapping used to assign loci to the nearest protein coding gene (see Materials and Methods for more detail).

In our primary analysis of global efficiency we found 14 genomic loci meeting genome-wide significance (Fig. 1A). This included associations in *PLCE1* (Phospholipase C Epsilon 1), *ANO1* (Anoctamin 1), *EPN2* (Epsin 2), *APOE* (Apolipoprotein E), *UFL1* (UFM1 Specific Ligase 1), *MRVL1* (also known as *IRAG1*, Inositol 1,4,5-Triphosphate Receptor Associated 1), *HSPG2* (Heparan Sulfate Proteoglycan 2), *ITGB5* (Integrin Subunit Beta 5) and *C10orf91* (chromosome 10 open reading frame 91), among others.

We found a single locus (sentinel variant rs187865392), near *GRIK2* (Glutamate Ionotropic Receptor Kainate Type Subunit 2), associated with local efficiency. Our GWAS of the resting state motor network revealed two loci, *EPHA3* (EPH Receptor A3) and *FAM3C* (Family With Sequence Similarity 3 Metabolism Regulating Signaling Molecule C). We also found two genomic loci for our GWAS of the subcortical-cerebellum network: *KCND2* (Potassium Voltage-Gated Channel Subfamily D Member 2) and *WNT16* (Wnt Family Member 16). Our GWAS of the visual I network (N = 36,150) revealed three loci all of which were also significant in our analyses of global efficiency (*PLCE1*, *UFL1*, and *C10orf91*). There were no other significant associations found for any other phenotype.

Our GWAS of RSFA revealed 24 significant genomic loci (Fig. 1B) and had a heritability estimate of 0.23 (SE 0.03; LDSC intercept = 1.03). Many of these overlapped with those we observed for global efficiency, with the addition of *SLC8A1* (Solute Carrier Family 8 Member A1), *RP11-8L2.1* (long interspersed ncRNA), *DGKB* (Diacylglycerol Kinase Beta), *EGFR* (Epidermal Growth Factor Receptor), *PAWR* (Pro-Apoptotic WT1 Regulator), *ATP2B1* (ATPase Plasma Membrane Ca2 + Transporting 1), *HIC1* (HIC ZBTB Transcriptional Repressor 1), *WWC2* (WW and C2 domain containing 2), and *STK32B* (Serine/Threonine Kinase 32B).

We next investigated whether the associations we observed for the network traits represented “true” associations with network integrity or were mediated by associations with the underlying vascular processes responsible for the BOLD response as, for example, the genetic correlation between global efficiency and RSFA was 0.79 (*p* = 5.4 × 10^–72^) indicating substantial shared genetic architecture. Following adjustment of global efficiency for average RSFA the estimated GWAS heritability fell markedly from 0.15 (SE 0.03) to 0.08 (SE 0.01). We also observed substantial attenuation in all 14 loci previously associated with global efficiency (Fig. 1C). Only three loci, two located on chromosome 10 (*PLCE1* and *C10orf91*) and one locus on chromosome 17 (*EPN2*) remained significant. Similar findings were observed for other phenotypes, particularly visual association 1 which was negatively correlated with global efficiency (r_g_ = − 0.56, *p* = 4.2 × 10^–10^). In contrast, adjusting for the related trait of systolic blood pressure exerted little effect on the associations we observed for global efficiency across the genome (Supplementary Fig. 13) and when we adjusted RSFA for global efficiency 18 of the 24 genome-wide significant loci remained (Supplementary Fig. 14).

Genetic associations and regional association plots for each locus are available in Supplementary Table 1 and Supplementary Figs. 15–61, respectively. Comparison of GWAS findings with and without adjustment for RSFA are presented in Supplementary Figs. 62–71.

Given our finding that RSFA appeared to drive most of the genetic variation observed in network measures, especially global efficiency, from herein we present a comparison of bioinformatic analyses of RSFA, global efficiency and global efficiency adjusted for RSFA. However, the results of these analyses for all network-related phenotypes are available in the supplementary material referred to.

### Multi-tissue transcriptome-wide association studies

**Figure 2:**
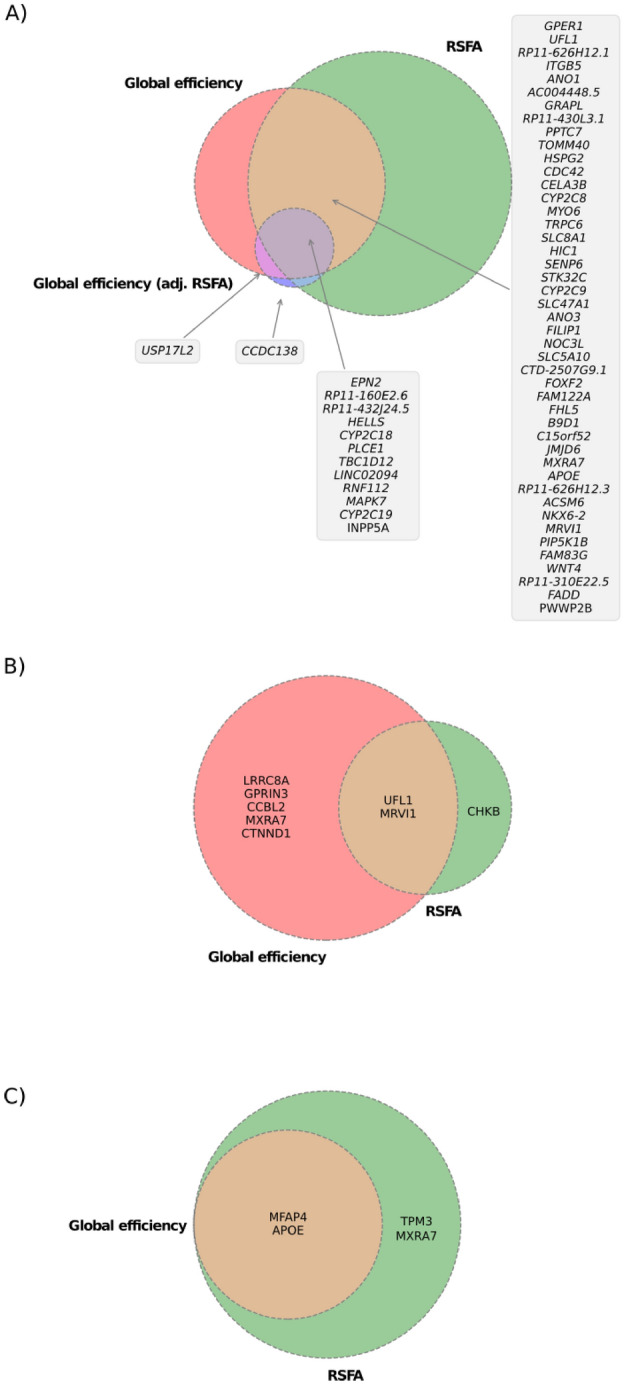
Proportional Venn diagrams for overlap of genes identified for (**A**) TWAS, (**B**) brain PWAS, and (**C**) plasma PWAS of global efficiency, RSFA and global efficiency adjusted for RSFA. TWAS, transcriptome-wide association study; PWAS, proteome-wide association study; RSFA, resting state fluctuation amplitudes. Omission of a network label from Venn diagrams indicates that no associations were found for that phenotype.

Our TWAS identified 157 genes whose predicted expression associated with RSFA (Supplementary Table 2). The most significant was *INPP5A* (Inositol Polyphosphate-5-Phosphatase A) followed by *PLCE1* which was also identified in our GWAS. Two genes, *CCDC138* (Coiled-Coil Domain Containing 138) and *SULT1C4* (Sulfotransferase Family 1C Member 4) were identified as having independent associations with global efficiency after adjusting for average RSFA—neither of which were prioritised in our analyses of global efficiency or RSFA separately (Fig. 2A).

### Proteome-wide association studies

#### Brain proteome

Our brain PWAS (Supplementary Table 3) for average RSFA identified associations with UFL1, MRVI1, and CHKB (Choline Kinase Beta). Two of these overlapped with proteins identified in our PWAS of global efficiency (Fig. 2B) which found independent associations for GPRIN3 (G Protein-Regulated Inducer Of Neurite Outgrowth 3), CTNND1 (Catenin delta-1), and CCBL2 (Cysteine Conjugate Beta Lyase 2/Kynurenine Aminotransferase 3). No proteins overlapped global efficiency, RSFA and global efficiency adjusted for RSFA.

#### Plasma proteome

We also performed plasma based PWAS (Supplementary Table 4), identifying MFAP4 (Microfibril-Associated Glycoprotein 4), APOE (isoform E2), TPM3 (Tropomyosin 3), and MXRA7 as being associated with average RSFA. Two of these (APOE, isoform E2 and MFAP4) overlapped with our analyses of global efficiency (Fig. 2).

### Properties and biological significance of associated variants

Gene set enrichment analyses at an FDR of 5%, revealed RSFA was enriched for terms relating to the regulation of lipid metabolic processes, including hydrolysis of phosphatidyl inositol-bisphosphate and the wider phosphatidylinositol signaling system, as well as regulation of platelet calcium homeostasis (Supplementary Tables 5-6).

### Cell type specificity of resting state brain networks

**Figure 3:**
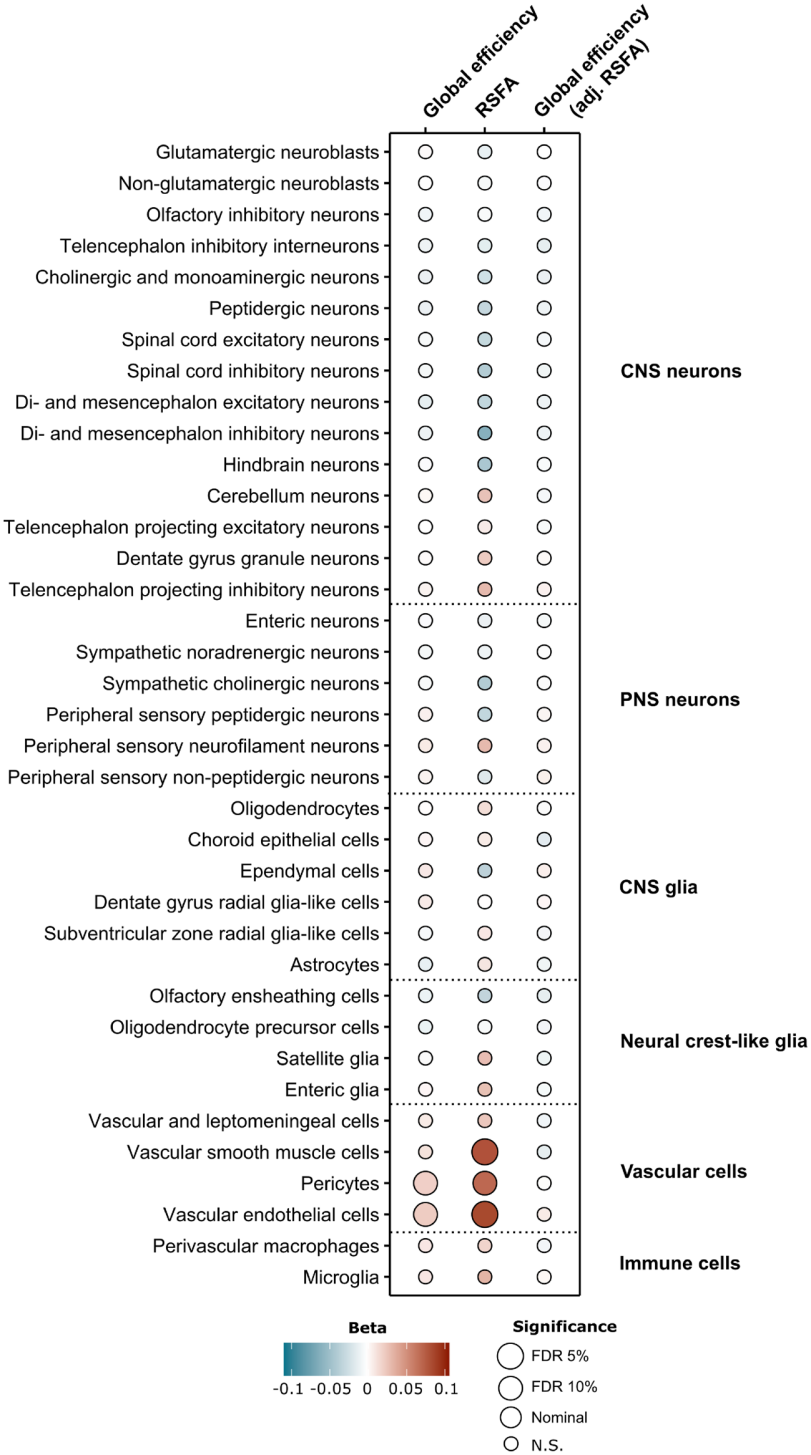
Bubbleplot of mouse nervous system single-cell RNA enrichment analyses for GWAS of global efficiency, resting state fluctuation amplitudes (RSFA) and global efficiency adjusted for RSFA. CNS, central nervous system; PNS, peripheral nervous system; FDR, false discovery rate; N.S., non-significant.

Our single-cell RNA specificity analyses based on the entire nervous system of the mouse revealed enrichment of vascular related cells, including pericytes, vascular endothelial cells, and vascular smooth muscle cells for genes associated with average RSFA (Fig. 3 and Supplementary Table 7). We also noted enrichment for several CNS glia cells and immune cells such as perivascular macrophages.

### Shared genetic architecture between brain networks and complex traits

**Figure 4:**
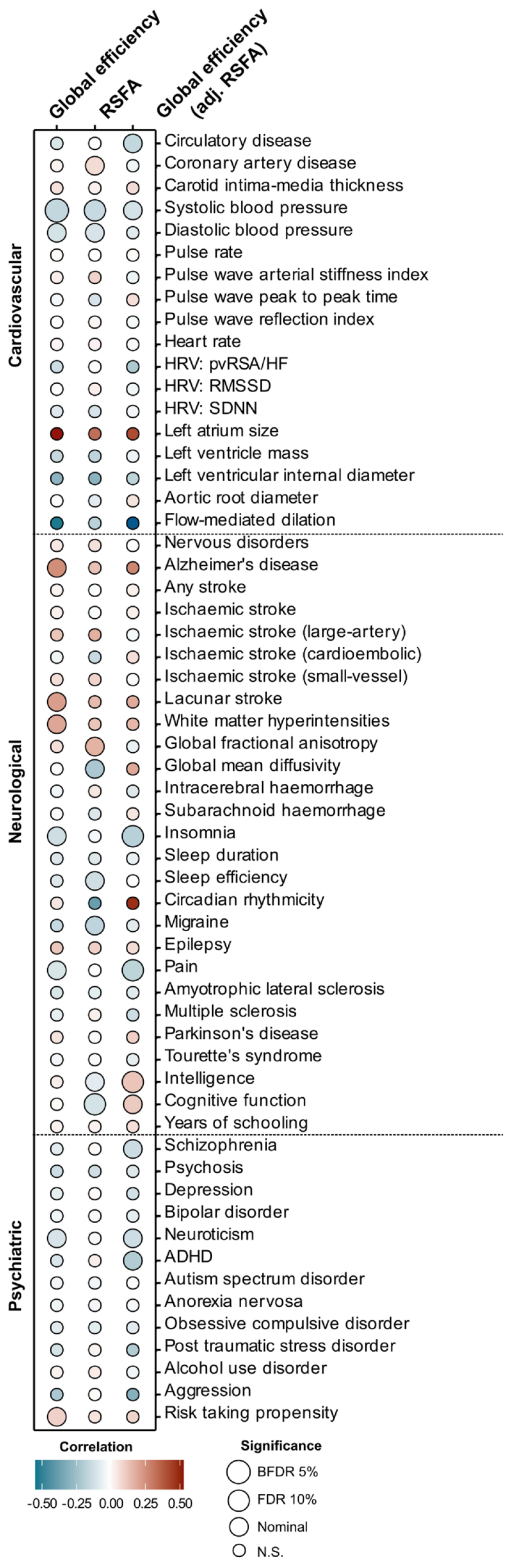
Bubbleplot for genetic correlations between global efficiency, resting state fluctuation amplitudes (RSFA) and global efficiency adjusted for RSFA and 58 cardiovascular, neurological and psychiatric traits. RSFA, resting state fluctuation amplitudes; HRV, heart rate variability; ADHD, attention deficit hyperactivity disorder; FDR, false-discovery rate; N.S., non-significant.

There were strong genetic correlations between our network measures (Supplementary Table 8). Average RSFA was significantly correlated with several cardiovascular metrics (Fig. 4 and Supplementary Table 9) including systolic (r_g_ = − 0.13, *p* = 0.002) and diastolic blood pressure (r_g_ = − 0.09, *p* = 0.02) as well as coronary artery disease (r_g_ = 0.08, *p* = 0.04). It was also associated with migraine (r_g_ = − 0.14, *p* = 0.04), and with global cognitive functioning (r_g_ = − 0.10, *p* = 0.003) and measures of intelligence (r_g_ = − 0.06, *p* = 0.05). Prior to adjusting for RSFA, global efficiency positively correlated with markers of small-vessel disease (white matter hyperintensities rg = 0.19, *p* = 0.025; lacunar stroke rg = 0.21, *p* = 0.009) and Alzheimer’s disease (rg = 0.24, *p* = 0.05) and was associated with both systolic and diastolic blood pressure, but all those associations were no longer significant after controlling for RSFA.

There were strong genetic correlations between our measures and those from earlier GWAS of rs-fMRI node amplitude and pairwise functional connectivity node pairs (Supplementary Table 10). In terms of brain morphology measures, RSFA was found to negatively correlate with the posterior cingulate brain surface area (plus diffusion-tensor imaging measures related to diffusivity in the cingulate gyrus and corpus callosum) and positively correlate with putamen volume. Global efficiency most strongly correlated with intracranial volume and the average surface area of the middle temporal gyrus. After adjusting global efficiency for average RSFA stronger correlations emerged for measures concerning brain surface area (Supplementary Table 11).

## Discussion

In this study we found that the global efficiency of resting state derived brain networks is moderately heritable, having identified 14 associated genomic loci meeting genome wide significance. Other resting state networks appeared less heritable, particularly local efficiency, with few genetic loci identified. However, we found that when we adjusted global efficiency for RSFA, the initial associations for global efficiency were very markedly attenuated. Therefore, we conclude that that the heritability of global efficiency is largely explained by the vascular component of the BOLD response as ascertained by RSFA, which itself has a significant genetic component. In total we identified 24 genomic loci associated with average RSFA, 157 genes whose predicted expression correlate with RSFA, as well as 3 proteins in the dorsolateral prefrontal cortex and 4 in plasma associated with RSFA. Our findings provide several important insights into the molecular basis of resting-state BOLD fluctuations and emphasise the importance of vascular processes underlying the BOLD response. Many of the associations we found have potential links with cardiovascular physiology or outcomes. Consistent with this single-cell RNA specificity analyses revealed enrichment of vascular related cells, including pericytes, vascular endothelial cells, and vascular smooth muscle cells for genes associated with average RSFA.

The gene with the smallest p-value in our GWAS of RSFA was *PLCE1* which encodes a protein that accelerates the hydrolysis of phosphatidylinositol-4,5-bisphosphate on the cell membrane to generate two secondary messengers—inositol-1,4,5-triphosphate and diacylglycerol. Consistent with our pathway enrichment analyses, inositol-1,4,5-triphosphate induces the release of Ca^2+^ in cells, and other genes identified in our analyses including *IRAG1* inhibit Ca^2+^ signalling and intracellular accumulation^18^ while *INPP5A* hydrolyses inositol-1,4,5-trisphosphate 5-phosphatase mobilises intracellular calcium and acts as a second messenger mediating cell responses to various stimulation. Inositol 1,4,5‐trisphosphate receptors, such as *IRAG1*, are recognised to play a vital role in vasodilation and blood pressure regulation^19^. *EPN2* is an epsin gene, recently shown to be under positive selection and thought to be associated with intelligence and speech in modern humans^20^. It has previously found to correlate with white matter hyperintensities^21^ and is known to affect Notch signalling^22^. *EPN2* has also been shown to interact with *IP3R1* (Inositol 1,4,5-trisphosphate receptor type 1) to accelerate atherosclerosis through triggering proteasomal degradation of this calcium release channel^23^.

Diacylglycerol is essential in the synthesis of phosphatidylcholine which is used to produce acetylcholine. Acetylcholine is an endothelium-dependent vasodilator that decreases with age^24^ and was recently demonstrated to control neurovascular coupling in blood microvessels through evoked Ca^2+^ oscillations^25^. Inositol 1,4,5‐trisphosphate receptors were shown to coordinate the repetitive release and reuptake of Ca^2+^ from the endoplasmic reticulum in response to acetylcholine, triggering the release of nitric oxide which would diffuse to nearby smooth muscle cells, activating guanylyl cyclase in the process to induce vasorelaxation. This is also supported by inositol 1,4,5‐trisphosphate receptor knockout mice having significantly reduced nitric oxide and blunted vasodilation in response to acetylcholine^19^. Diacylglyrol can also activate protein kinase C, a pathway recently highlighted as a potentially safe therapeutic target for reducing risk of hypertension^26^. We observed significant genetic correlations between RSFA and both systolic and diastolic blood pressure which these findings indirectly support. Protein kinase C is also a target of Bryostatin, a blood–brain barrier penetrant drug currently undergoing clinical trials for the treatment of Alzheimer’s disease^27^ that targets innate immunity in neuroinflammation and amyloid precursor protein α-secretases.

Several other genes identified in our analysis of RSFA have been found to associate with cerebrovascular haemodynamic measures. *IRAG1* is associated with arterial stiffening^28^, while genetic variants in *PLCE1* have been found to correlate with distensibility of the proximal descending aorta^29^ and flow reversal^30^. *ANO1* has been implicated in cerebral artery constriction^31^ and *UFL1* and *FHL5* are thought to regulate cerebral blood flow^32^. *UFL1, FHL5* and *PLCE1* have also has also been associated with migraine^33^. *LMOD1*, identified by our TWAS, is a coronary artery disease associated locus believed to regulate contractility in visceral smooth muscle cells^34^.

We found genetic variants at the *APOE* locus were associated with RSFA and our plasma proteomic analysis prioritised the E2 isoform. *APOE2* is considered “protective” of Alzheimer’s disease, with studies that have implanted the human isoform of the gene into mice with amyloid plaques showing shrinkage of some plaques and a decrease in plaque-associated damage in their brains^35^. *APOE2* has low affinity binding to low-density lipoprotein cholesterol and heparan sulfate proteoglycans^36^, both of which have been associated with the uptake of amyloid-β peptides in the brain^37^ We identified the heparan sulfate proteoglycan gene *HSPG2* as being associated with RSFA. Conditional *HSPG2* knockout mice are known to express lower levels of perlecan in the extracellular matrix which helps maintain blood–brain barrier integrity and improves pericyte migration^38^. Pericytes determine the direction of blood flow at capillary junctions^39^ and have recently been demonstrated to exert a substantial influence on blood flow^40^ which is consistent with our cell-type specificity analyses. Platelet calcium homeostasis was also identified as a possible pathway underlying resting-state BOLD fluctuations in our analyses. Perlecan Domain V interacts with platelet-derived growth factor BB to enhance pericyte migration in support of blood–brain barrier maintenance and repair following ischaemic stroke^38^. Diacylglycerol, via activation of protein kinase C, has also been described as a bi-directional regulator of platelet reactivity^41^.

Outside of RSFA, we found a single locus on chromosome 6 harbouring *GRIK2* that was associated with local efficiency. GRIK2 affects kainite, a neuroexcitatory amino acid agonist that activates glutamate receptors, and has been demonstrated to be important for synaptic transmission^42^ as well as having been implicated in a number of neurological conditions^43^. *KCND2* was identified in our GWAS of the subcortical-cerebellum network, it is a voltage-gated potassium channel gene known to associate with rare neurological conditions such as early myoclonic encephalopathy and developmental and epileptic encephalopathy 3. It has recently been suggested to limit burst firing in the thalamic reticular nucleus^44^. We identified *FAM3C*, which encodes interleukin-like epithelial-to-mesenchymal transition inducer, in our GWAS of the motor subnetwork. Levels of FAM3C have been shown to decline as Huntington’s disease (a condition with substantial impairments in involuntary movement) progresses^45^.

Our study has several strengths. It included a large cohort of over 36,000 participants. All were scanned on one of three identical MR scanners using identical acquisition parameters, and analysed via a single pipeline. However, it also has limitations. Participants were of European ancestry therefore our findings cannot be guaranteed to generalise to individuals of all ancestries. Furthermore, to maximise statistical power to identify associations we chose to include all participants in the discovery cohort in lieu of using a subset as a replication sample. It would be useful to perform similar analyses in independent datasets to demonstrate that our findings are reproducible. To minimise potential bias, we did not adjust our analyses for any heritable covariates^46^. It is also worth noting that the rs-fMRI performed in UK Biobank operated under an “eyes open” protocol which has been demonstrated to affect resting state connectivity differently than “eyes closed” procedures^47^. Indeed, recent studies have shown that light influences cerebral blood flow by decreasing calcium in arteriolar smooth muscle cells, leading to pronounced vasodilation, without eliciting neuronal or astrocyte excitation^48^. The extent to which this may have influenced our findings is difficult to quantify but it is worth highlighting that one of our lead genomic loci, *PLCE1*, which was associated with global efficiency even after adjusting for RSFA is highly expressed in the retina (particularly Müller glia cells)^49^ and we observed strong genetic correlations between both global efficiency and RSFA, and visual subnetworks 1 and 2. We focussed on a whole-brain average measure of RSFA, however, RSFA is known to differ across brain regions^2^. Subnetworks are thought to coalesce to shape global neural activity^50^ and as such investigation of the genomic architecture of RSFA in specific subnetworks would be an interesting avenue for future investigation. As would studies of other graph metrics, such as transitivity, modularity, assortativity, or global brain connectivity. It is also worth noting that the measures described here reflect static functional connectivity not dynamic^51,52^. The latter is purported to provide a better reflection of the reality of functional connections, in that they are not static in nature, but time varying, and is becoming more widespread in the literature. Similarly, there are limitations in calculating efficiency using functional correlation matrices as this relies on taking the shortest path between nodes (after transforming edge weights from magnitude to distance using the inverse function). Paths over edges defined by correlations are difficult to interpret, whereas defining paths over structural edges (which signals can traverse) makes more conceptual sense. However, the measures we use are still widely used to illustrate functional connectivity in the brain.

In summary, we found that the heritability of global efficiency is largely explained by RSFA. We identified 24 genomic loci associated with average RSFA. Our analyses highlight differences in the likely cell types underlying these processes (vascular smooth muscle and endothelial cells plus pericytes for global efficiency), and emphasise the function of lipid transport, store-operated calcium channel activity, and inositol 1,4,5-trisphosphate binding in resting-state BOLD fluctuations.

## Materials and methods

### Sample

Participants were drawn from the UK Biobank, a population based study of 502,535 individuals aged 40–69 at study entry, recruited between May 2006 and July 2010 from 22 assessment centres covering all major areas of the UK^53^. Of these, approximately 100,000 are scheduled to have a brain MRI, and we used data from the first 36,858 (imaging study data release February 2020). The mean age at the time of brain imaging was 63.6 (standard deviation 7.5) years. We excluded 707 participants with a history of stroke or other major central nervous system (CNS) disease (eg., multiple sclerosis, Parkinson’s disease, dementia or any other CNS neurodegenerative condition). A full list of International Classification of Disease (ICD) versions 9 and 10 diagnosis codes, and UK Biobank self-report codes can be found in Supplementary Table 12. UK Biobank received ethical approval from the Research Ethics Committee (reference 11/NW/0382), all methods were performed in accordance with the relevant guidelines and regulations, and all participants provided written informed consent.

### MRI imaging and functional networks

An extended description of the MRI acquisition protocol in UK Biobank is available elsewhere^54^. Briefly, all MRI data were acquired on three identical Siemens Skyra 3 T scanners (Siemens Medical Solutions, Erlangen, Germany) using a standard Siemens 32-channel head coil. The resting state fMRI sequence was a gradient echo EPI sequence covering the whole brain. During the sequence (performed at 2.4 × 2.4 × 2.4mm^3^ resolution, with 64 slices) participants were instructed to relax, keep their eyes fixated on a cross-hair, and “think of nothing in particular”. Four hundred and ninety brain volumes (or timepoints) were acquired in an acquisition that took six minutes. These images were then analysed to assess signal variation across the time course.

The “raw” data were processed by UK Biobank as described in detail^55^, this involved motion correction, grand-mean intensity normalisation, high-pass temporal filtering (using Gaussian-weighted least-squares straight line fitting, with sigma = 50.0 s), echo-planar imaging unwarping (using fieldmaps additionally acquired to remove geometric distortions) and the removal of structured artefacts. We only used data deemed of “useable quality” (~ 96%) based on the quality control pipeline implemented by the central UK Biobank team. A group principal component analysis and subsequently an independent component analysis (ICA) was performed on 4100 datasets to produce a ‘parcellation’ of the 55 largest components, each component relates to a volume of brain tissue. The partial temporal correlation between the signal time course from each pair of components was calculated and used to create a correlation matrix. The matrices were thresholded to include only the top 10% of connections.

The Brain Connectivity Toolbox^56^ was then used with the correlation matrices to derive a number of network measures. For this work we calculated the weighted global efficiency which is the average inverse shortest path length between each pair of nodes, or brain regions, which is a measure of the integrationof the brain and weighted local efficiency which is calculated for each node as the inverse path length between those nodes connected to it and averaged, this is related to network segregation or capability to perform specialised processing within densely interconnected brain regions. Average connection values for eight functional networks, as defined in Finn et al.^5^ were also calculated. Following the analysis in Zhao et al.^12^ the 55 components from the ICA described above were assigned to one or more of the eight networks: default mode, medial frontal, fronto-pariental, subcortical, motor, visual association, visual 1 and visual 2. For each of these networks the mean within-network functional connectivity between each pair of components was calculated to give an overall measure of the connectivity for the network for each subject.

We also derived a whole brain average of the area specific resting state fluctuation amplitudes (RSFA)—thought to reflect the vascular component of BOLD signals^17^—and performed a GWAS of this, as well as network measures adjusted for RSFA to determine whether signals identified were common or persisted after controlling for the average spontaneous vascular response. The RSFA for each of the 55 regions described earlier was calculated by UK Biobank^55^ and then averaged to give a measure for the whole brain.

We excluded extreme values on any network measure, defined as values ± 5 standard deviations from the mean (three values for global efficiency and RSFA, none for local efficiency).

In addition we extracted the diffusion tensor and brain volume measures derived by UK Biobank.

## Genotyping, quality control and imputation

Two genotyping arrays specifically designed for UK Biobank were used to genotype individuals (Affymetrix UK BiLEVE or UK Biobank Axiom Array). Quality control (QC), pre- and post-imputation, as well as the full imputation pipeline were performed centrally by UK Biobank^57^. In short, genotype QC metrics included a minor allele frequency (MAF) > 0.001, call rate > 98%, and Hardy–Weinberg equilibrium (*p* > 10^−6^). The pre-imputation sample exclusion criteria included a call rate of > 95%, heterozygosity rate > median + 3 × IQR, plus removal of gender mismatches, duplicates, and outliers from principal component analysis with reference samples from the 1000 Genomes Project. All genotypes were on the forward stand of the Genome Reference Consortium Human Build 37 (GRCh37) and imputation was performed using a combined Haplotype Reference Consortium and UK10K haplotype panel.

### Additional demographic and phenotypic measures

We further recorded information on the participants age at the time of scanning, their sex, and the imaging centre they attended (Newcastle upon Tyne, Stockport, or Reading), as well as an indicator of head motion during the resting state component of the scanning session^58^. We also noted the array used when genotyping DNA samples.

### Genome-wide association analyses

Prior to carrying out our GWAS we fit a linear regression of each network measure on age, age-squared and sex (and their interaction terms), as well as technical covariates of head motion (natural-log transformed and squared) and imaging centre. For analyses of network measures accounting for RSFA we also included RSFA values in the model as covariates. The residuals from these models were extracted, inverse normalised and used as the outcome variables in our GWAS. Assuming an additive genetic model we performed statistical analyses of genotype doses in BOLT-LMM^59^ using a linear mixed model to account for cryptic relatedness. We included the first 10 principal components of ancestry and an indicator of genotyping array in these models as potential genomic confounds. Quality control thresholds applied to the GWAS included testing of variants with a MAF > 0.001 and an imputation quality score > 0.4. We used the linkage disequilibrium score (LDSC) intercept^60^ to discern genomic inflation from “true” polygenicity. We performed positional mapping to protein coding genes in Ensembl v92 within FUMA^61^ using a maximum window of ± 10 kb from the variant in the locus with the lowest p-value (herein referred to as the “sentinel variant”). Secondary signals at a locus were defined as variants with an r^2^ < 0.1 with the sentinel variant using a reference panel based on 10,000 randomly selected individuals of European ancestry in UK Biobank. Single-nucleotide polymorphism (SNP) based heritability was also calculated using LDSC. Genome-wide statistical significance was set as *p* ≤ 5 × 10^−8^.

#### Transcriptome-wide association study

We performed a transcriptome-wide association study (TWAS) using S-PrediXcan^62^ for 22,291 genes with weights derived for 49 tissues from the Genotype-Tissue Expression GTEx project v8^63–65^. TWAS leverage studies with expression and genotype data to discover and/or prioritise gene–trait associations from GWAS datasets. Gene-level predictive models of variation in expression are learned from these data and then applied to calculate gene expression profiles associated with the phenotype of interest. To quantify the overall gene-trait association we aggregated findings into a single metric^66^. The significance threshold was set to a 5% false discovery rate (FDR) using the Benjamini–Hochberg procedure.

#### Proteome-wide association studies

FUSION^67^ was used to perform proteome-wide association studies (PWAS) based on weights derived for the abundance of 1,464 proteins in the dorsolateral prefrontal cortex^68^ and 1,054 plasma proteins^69,70^. Weights for PWAS are calculated in the same manner as those from imputed TWAS in that the effect of SNPs on protein abundance are computed using multiple models and the “most predictive” selected for upstream analyses. GWAS summary statistics are transformed to *z* scores and protein abundance calculated as the linear sum of *z* score × PWAS weight for the independent SNPs at a given locus. We selected an FDR of 5% within each brain network measure to account for multiple testing whilst acknowledging the modest correlation between proteins.

#### Gene enrichment and pathway analysis

We conducted gene based tests for 19,253 genes and gene set enrichment analysis with MAGMA v1.08b^71^ using an annotation window of 35 kb upstream and 10 kb downstream of genes and adopting a 5% FDR for significance^72^. We excluded the major histocompatibility complex region from annotations (spanning from *MOG* to *COL11A2*; chr6:28,477,797–33,448,354) given its complex LD structure. Briefly, MAGMA averages p-values of SNPs located within a given annotation window of a gene (taking into account LD structure) and transforms this statistic to a *z* score. This is then used to test whether a particular gene set associates with the gene-level *z* score using linear regression.

#### Cell-type specificity

To examine specific cell types underlying our brain network measures we integrated single-cell transcriptomic data from the entire mouse nervous system^73^ alongside the results of our GWAS to perform gene-set enrichment tests using MAGMA v1.08b^71^ as described in detail elsewhere^72^. In short, any genes: (a) with non-unique names, (b) not expressed in any cell types, (c) that do not code for a protein, and (d) that had no expert-curated 1:1 orthologs between mice and humans, were filtered out. Gene expression was then scaled to 1 million unique molecular identifiers for each cell type. Following this the same procedures outlined above to calculate gene-set enrichment were used. In these analyses we adjusted for gene size, gene density, the mean sample size for tested SNPs per gene, the inverse of the minor allele counts per gene and the log of these metrics. An FDR of 5% was selected to account for the testing of multiple cell types within each brain network measure.

#### Genetic correlation with other traits

Genetic correlations were performed using pre-calculated weights derived for European ancestry individuals (based on the 1000 Genomes, Phase 3 release) in LDSC^74^. We downloaded GWAS summary statistics for 58 neurological, cardiovascular and psychiatric conditions, plus related quantitative traits. We also performed genetic correlation analyses with summary results from an earlier rs-fMRI GWAS of 201 node amplitude/pairwise functional connectivity node pairs^12^ to examine the magnitude of genetic sharing between our network measures and these lower level, constitute parts. Further to this we carried out correlation analyses with other MRI derived measures including volume of various anatomical structures within the brain and thickness/area size of different cortical regions, as well as brain activity recorded by electroencephalogram.

To account for multiple testing across each of these sets of analysis for each brain network measure we adopted an FDR of 5%. For a full list of traits and resources investigated please see Supplementary Table 13.

## Supplementary Information

Supplementary Information 1.

Supplementary Information 2.

## Data Availability

Summary statistics from the GWAS performed here have been archived on Zenodo (https://zenodo.org/record/7034803). Individual level data used in this study are available to *bona fide* researchers upon application to UK Biobank. Network measures derived for this study will be returned to UK Biobank for wider use by the research community. Auxiliary data for all other analyses has been made available by the developers of software packages cited.
