# Supplementary material for: Genome-wide association study of the human brain functional connectome reveals strong vascular component underlying global network efficiency
Source: Sci Rep. 2022 Sep 2;12:14938. doi: 10.1038/s41598-022-19106-7 (PMC9440133; doi:10.1038/s41598-022-19106-7)
Supplement: Supplementary file 1 — Supplementary Information 1. [file 41598_2022_19106_MOESM1_ESM.pdf]

Supplementary material to:
Genome-wide association study of the human brain functional connectome reveals strong vascular component underlying global network efficiency (Bell, Tozer & Markus)

Table of Contents

Q-Q plots ... 3
- Global efficiency ... 3
- Local efficiency ... 4
- Default-mode network ... 5
- Medial frontal network ... 6
- Frontoparietal network ... 7
- Subcortical-cerebellum network ... 8
- Motor network ... 9
- Visual association network ... 10
- Visual network I ... 11
- Visual network II ... 12
- Global efficiency adjusted for RSFA ... 13
- RSFA ... 14

Additional adjustments ... 15
- Systolic blood pressure ... 15
- Global efficiency and RSFA mutually adjusted ... 16

Regional association plots ... 17
- Global efficiency - *HSPG2* ... 17
- Global efficiency - *ITGB5* ... 18
- Global efficiency - *FOXQ1* ... 19
- Global efficiency – *RP11-474L11.3-6ENP6* ... 20
- Global efficiency - *UFL1* ... 21
- Global efficiency - *PIP5K1B* ... 22
- Global efficiency - *PLCE1* ... 23
- Global efficiency - *C10orf91/INPP5A* ... 24
- Global efficiency - *MRVI1* ... 25
- Global efficiency - *ANO3* ... 26
- Global efficiency - *ANO1* ... 27
- Global efficiency - *TRPC6* ... 28
- Global efficiency - *EPN2* ... 29
- Global efficiency - *APOE* ... 30
- Local efficiency - *GRIK2* ... 31
- Motor network - *EPHA3* ... 32
- Motor network - *FAM3C* ... 33
- Subcortical-cerebellum network - *KCND2* ... 34
- Subcortical-cerebellum network - *WNT16* ... 35
- Visual network I - *UFL1* ... 36
- Visual network I - *PLCE1* ... 37
- Visual network I - *C10orf91* ... 38
- Global efficiency adjusted for RSFA – *PLCE1* ... 39
- Global efficiency adjusted for RSFA – *C10orf91* ... 40
- Global efficiency adjusted for RSFA – *EPN2* ... 41
- RSFA – *HSPG2* ... 42
- RSFA – *SLC8A1* ... 43

RSFA – *ITGB5* 44
RSFA – *STK32B* 45
RSFA – *RP11-8L2.1* 46
RSFA – *WWC2* 47
RSFA – *FOXQ1* 48
RSFA – *SENP6* 49
RSFA – *UFL1* 50
RSFA – *DGKB* 51
RSFA – *EGFR* 52
RSFA – *PIP5K1B* 53
RSFA – *PLCE1* 54
RSFA – *C10orf91-INPP5A* 55
RSFA – *IFITM2* 56
RSFA – *MRVI1* 57
RSFA – *ANO3* 58
RSFA – *ANO1* 59
RSFA – *TRPC6* 60
RSFA – *PAWR* 61
RSFA – *ATP2B1* 62
RSFA – *HIC1* 63
RSFA – *EPN2* 64
RSFA – *APOE* 65
Associations without (upper) and with (lower) adjustment for RSFA 66
Global efficiency 66
Local efficiency 67
Default-mode network 68
Frontoparietal network 69
Medial frontal network 70
Motor network 71
Subcortical-cerebellum network 72
Visual association network 73
Visual network 1 74
Visual network 2 75

Q-Q plots

Global efficiency

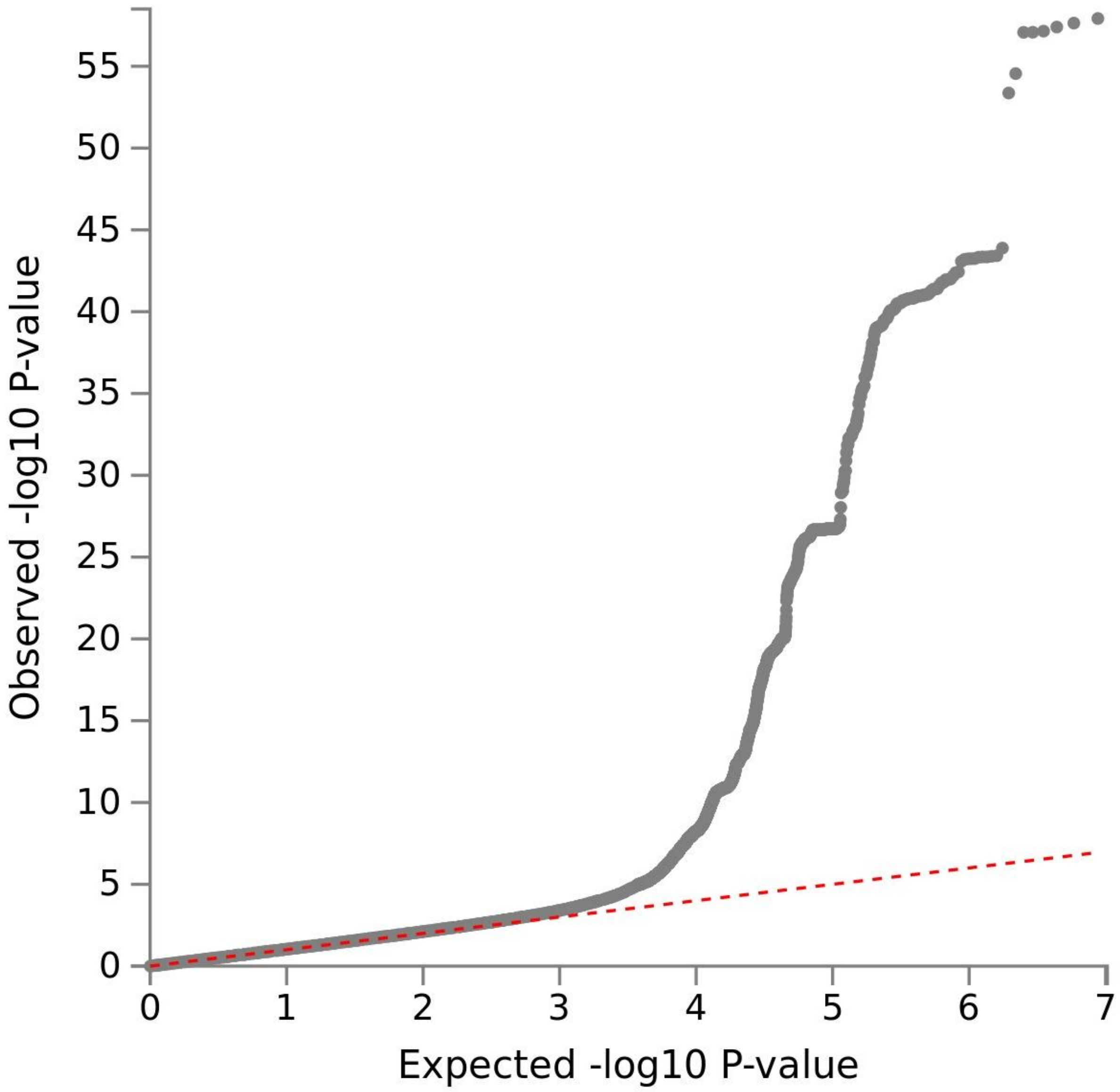

Supplementary Figure 1- Q-Q plot for GWAS of global network efficiency

Local efficiency

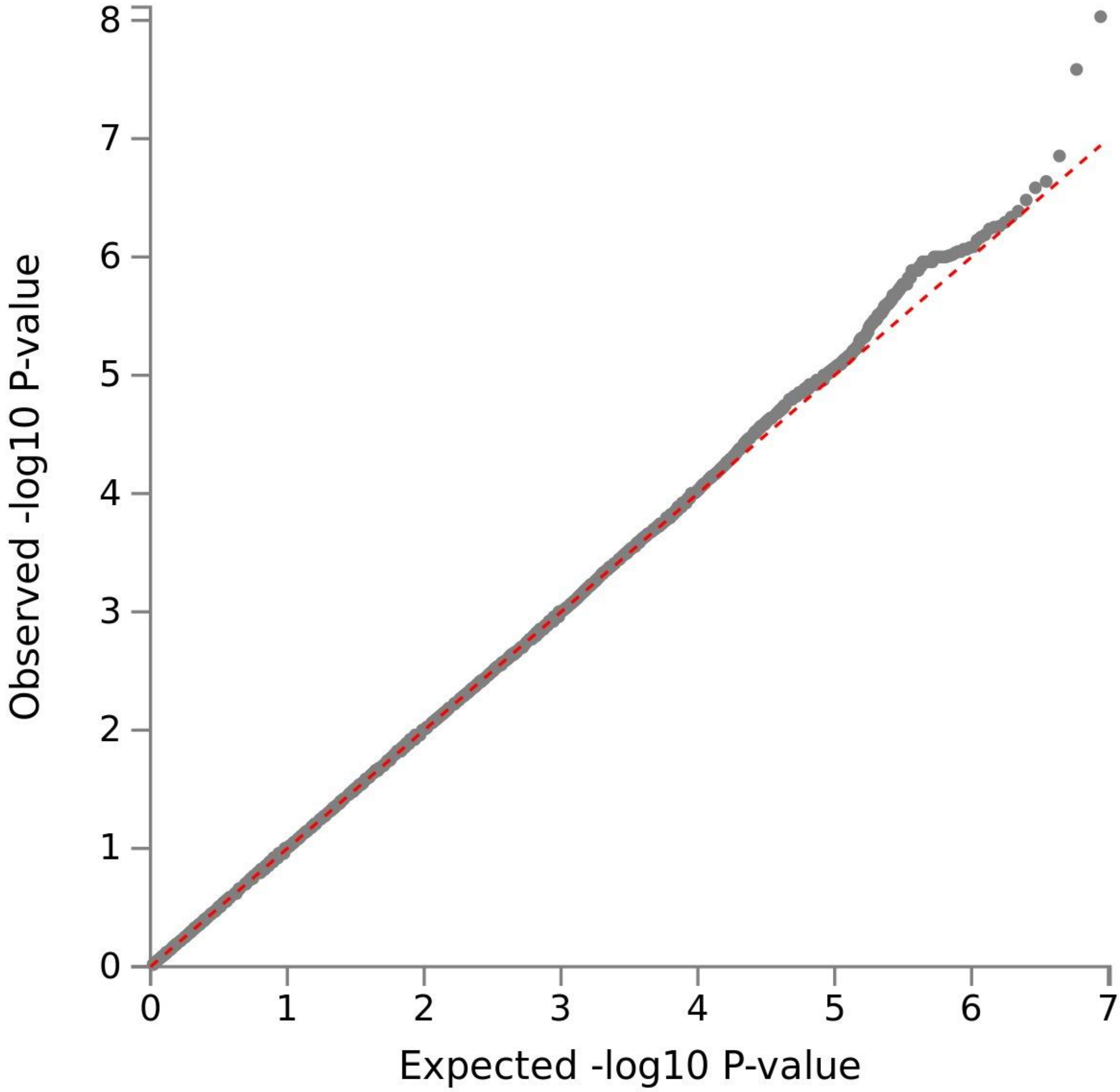

Supplementary Figure 2- Q-Q plot for GWAS of local network efficiency

Default-mode network

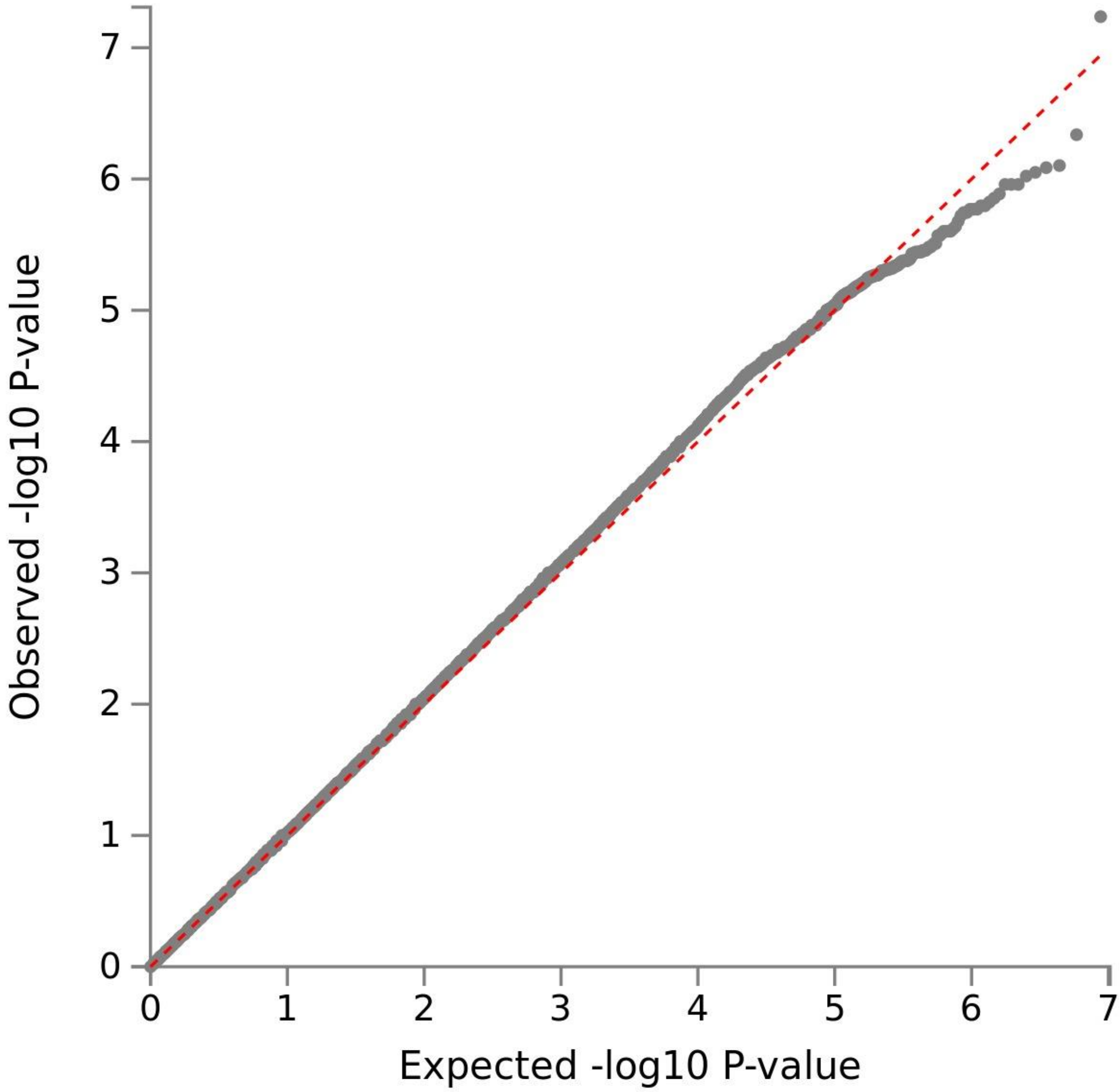

Supplementary Figure 3- Q-Q plot for GWAS of the default-mode network

Medial frontal network

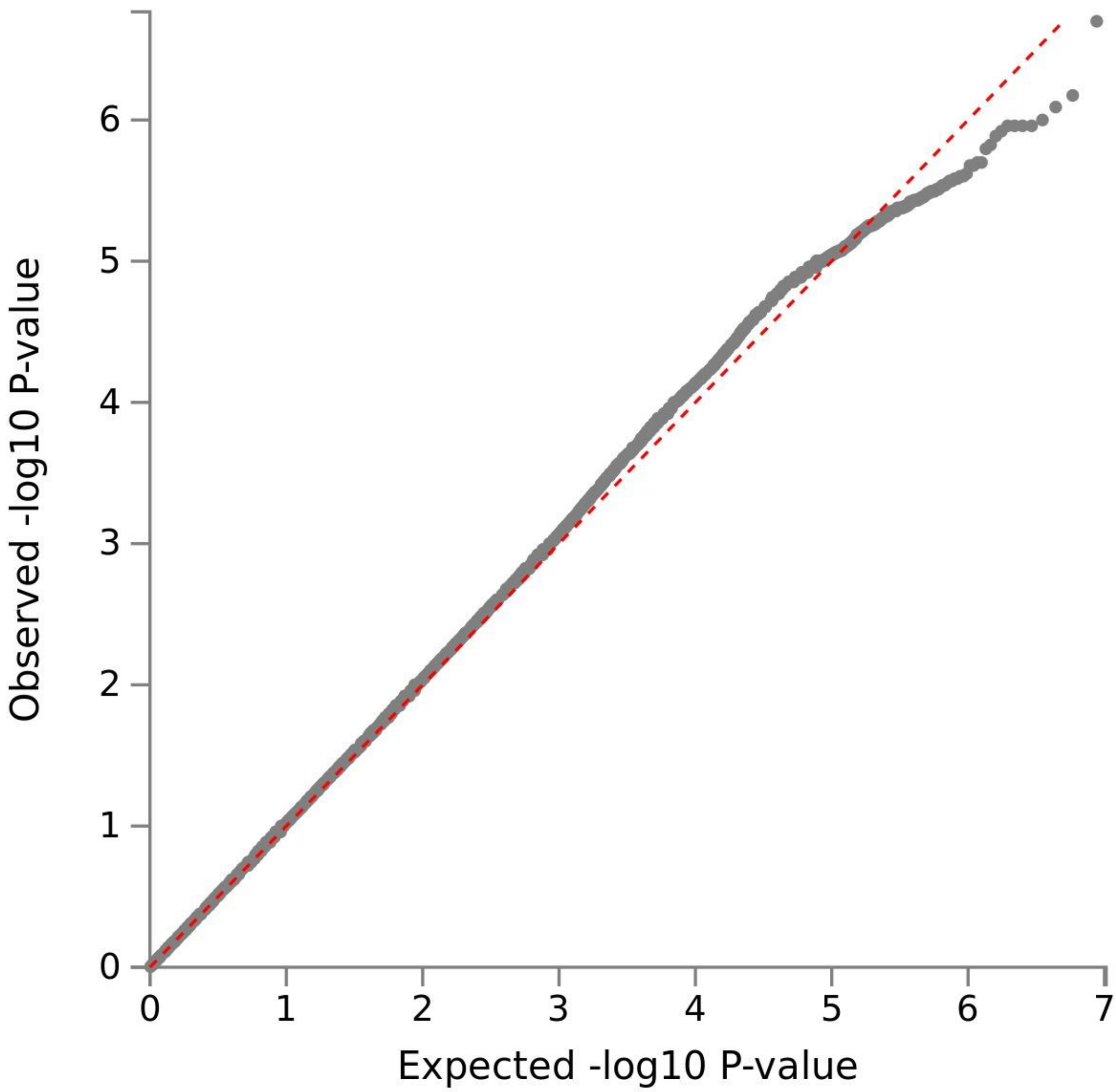

Supplementary Figure 4- Q-Q plot for GWAS of the medial frontal network

Frontoparietal network

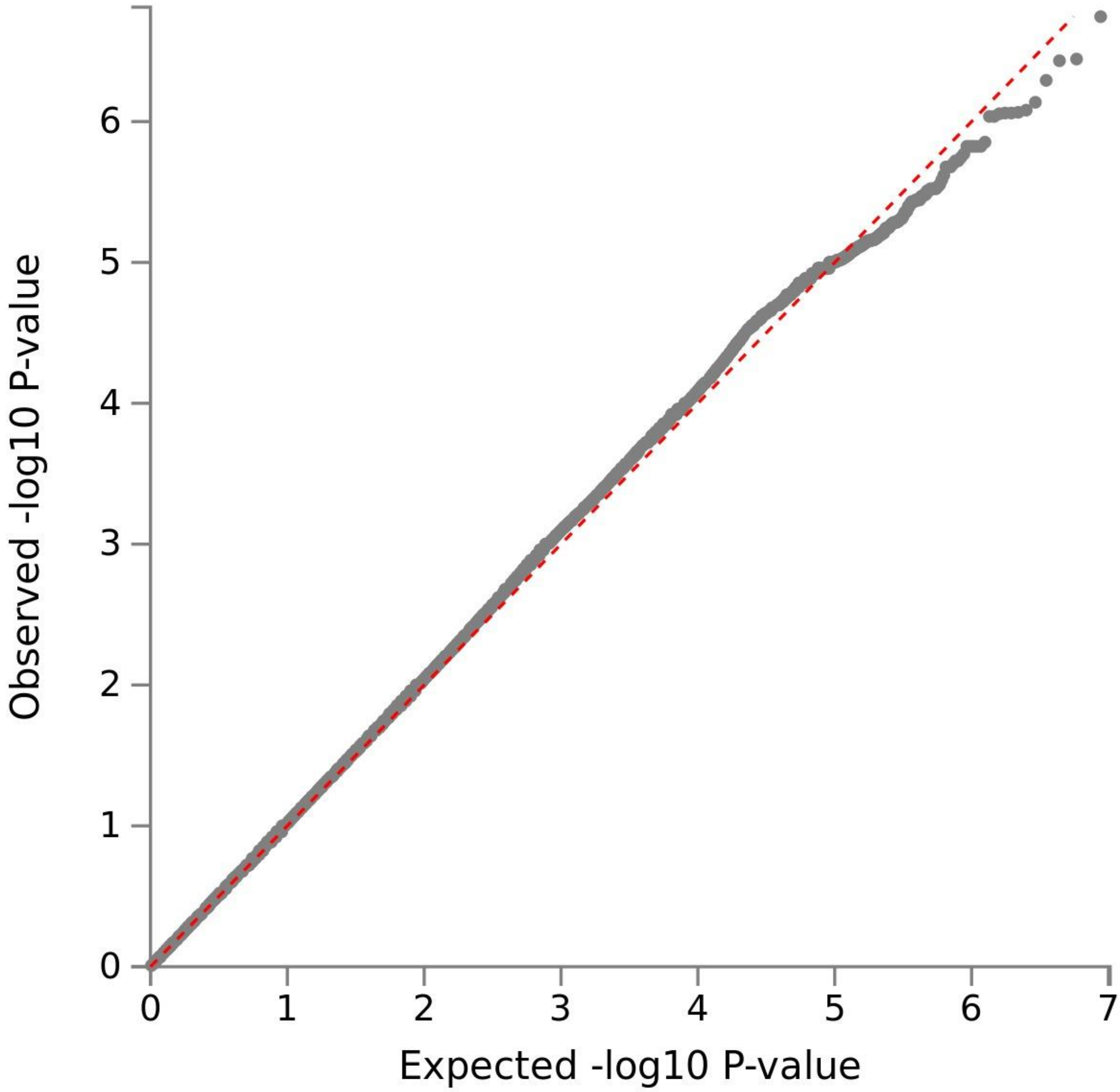

Supplementary Figure 5- Q-Q plot for GWAS of the frontoparietal network

Subcortical-cerebellum network

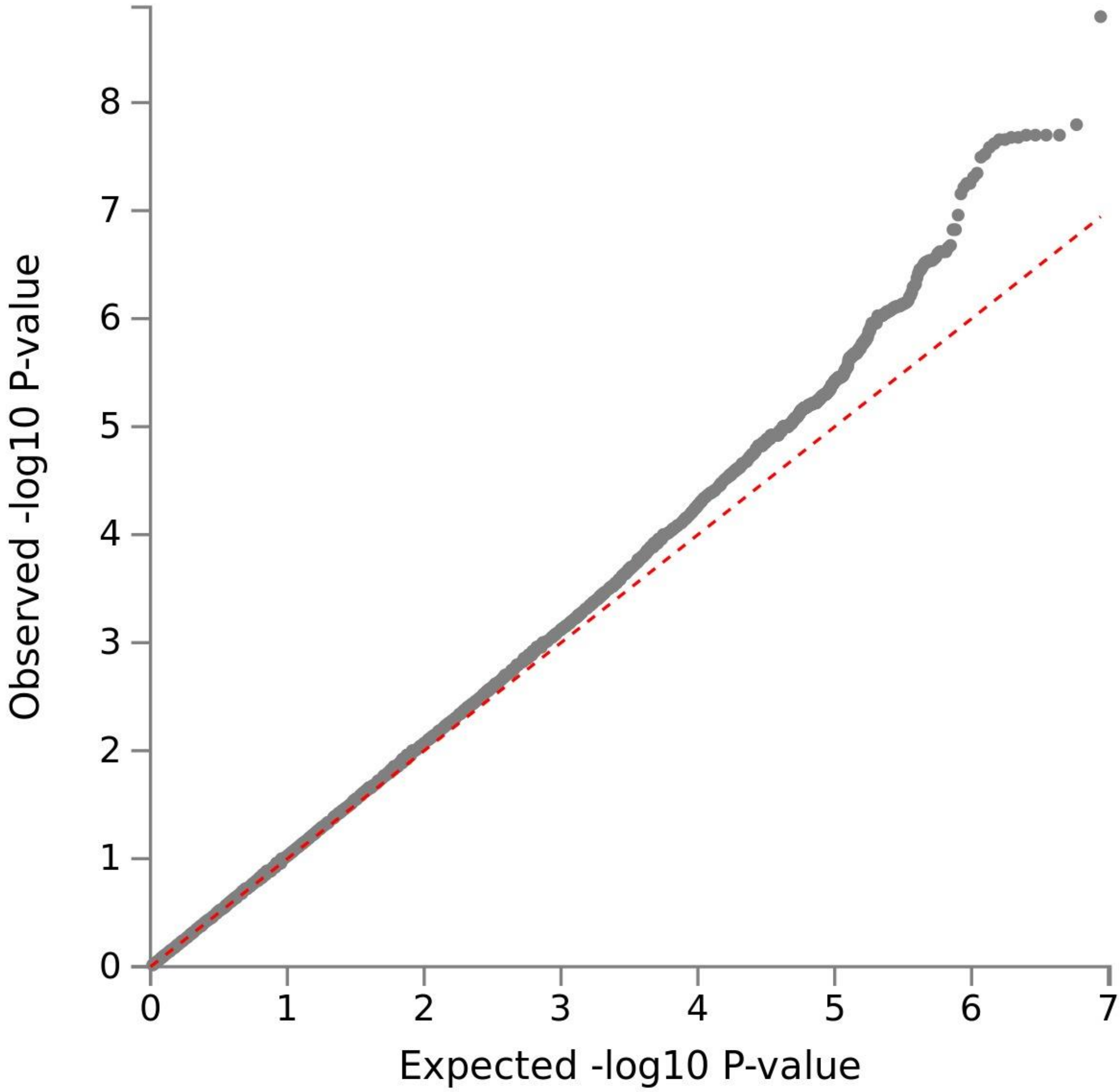

Supplementary Figure 6- Q-Q plot for GWAS of the subcortical-cerebellum network

Motor network

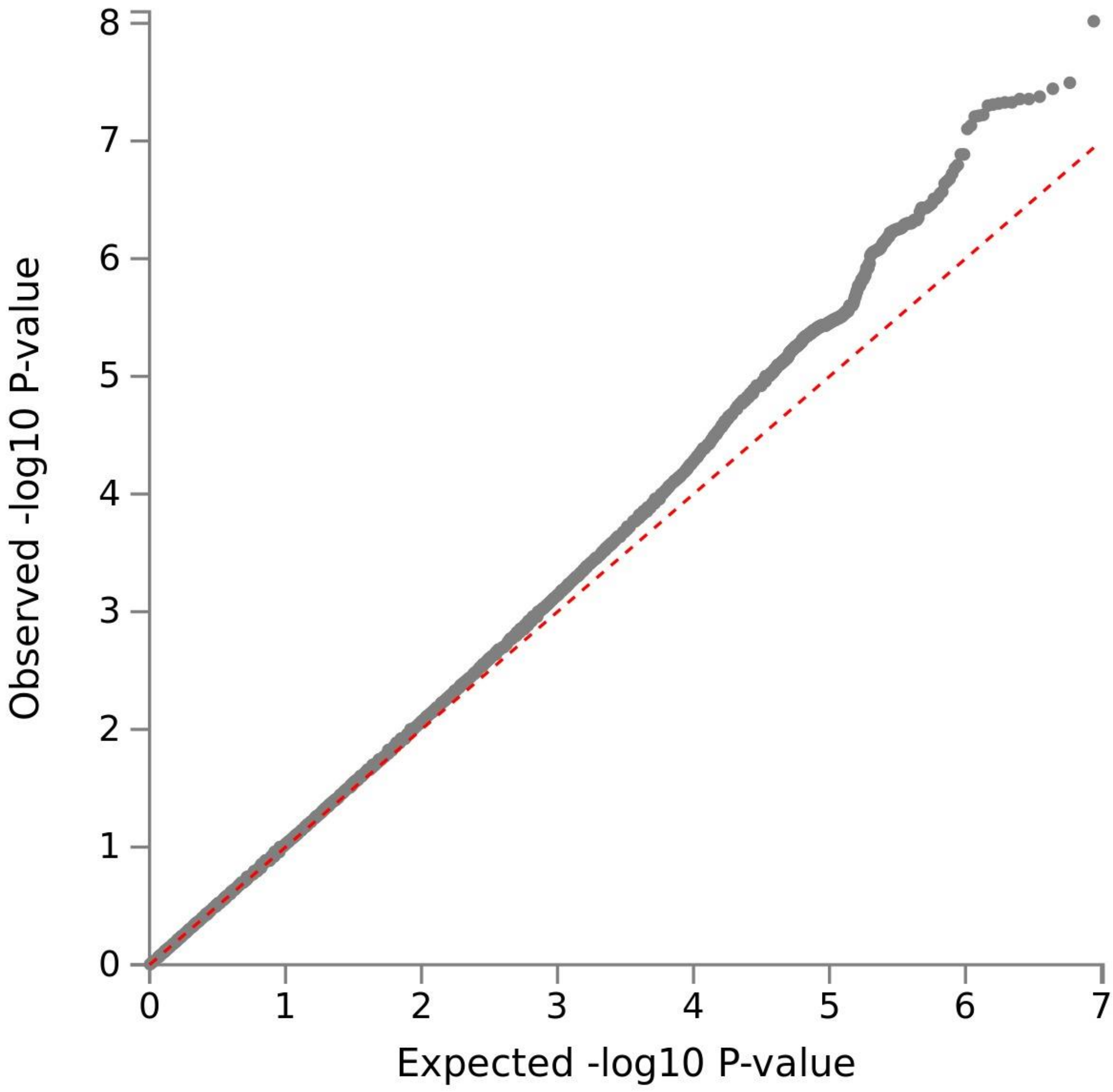

Supplementary Figure 7- Q-Q plot for GWAS of the motor network

Visual association network

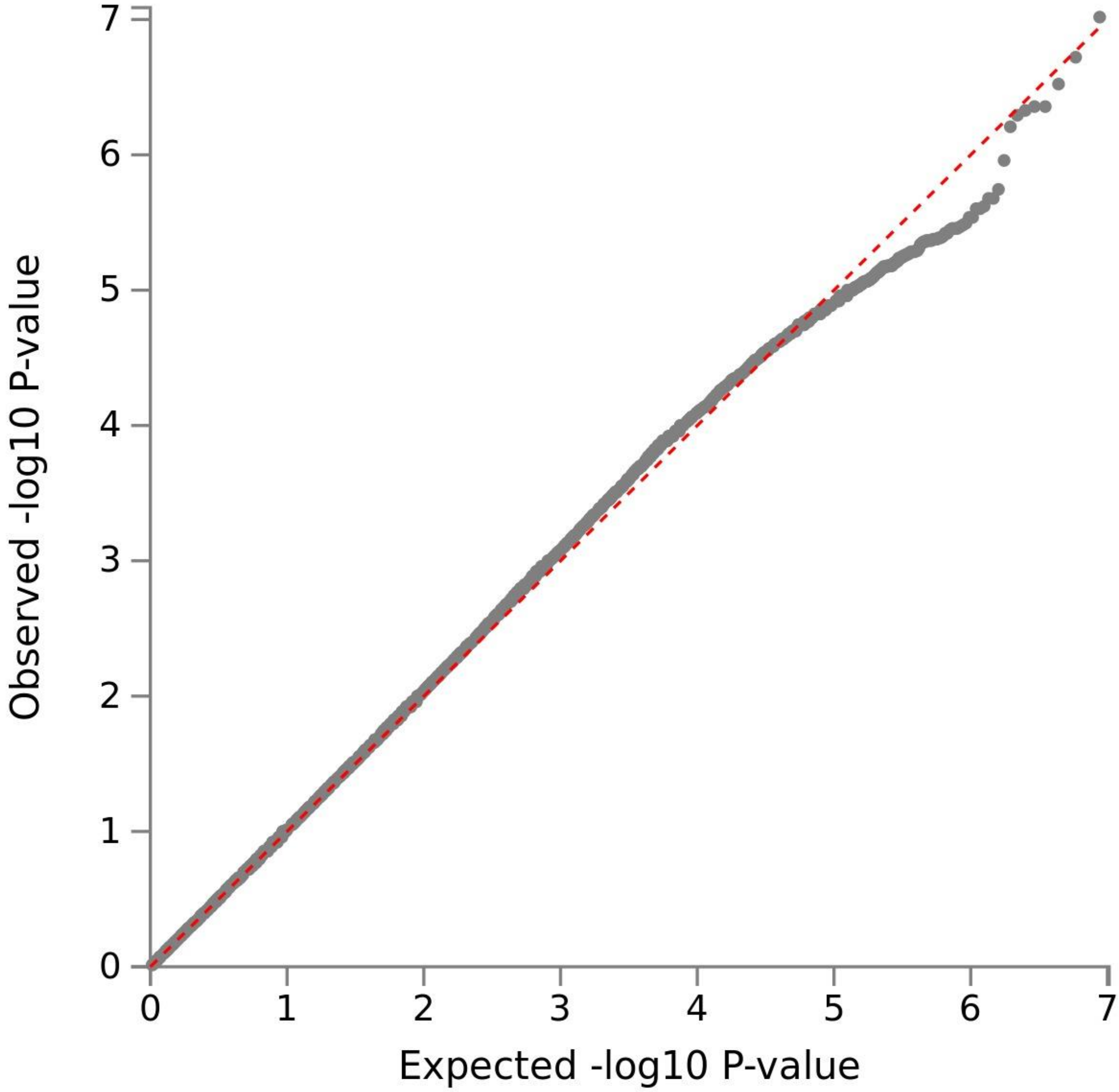

Supplementary Figure 8- Q-Q plot for GWAS of the visual association network

Visual network I

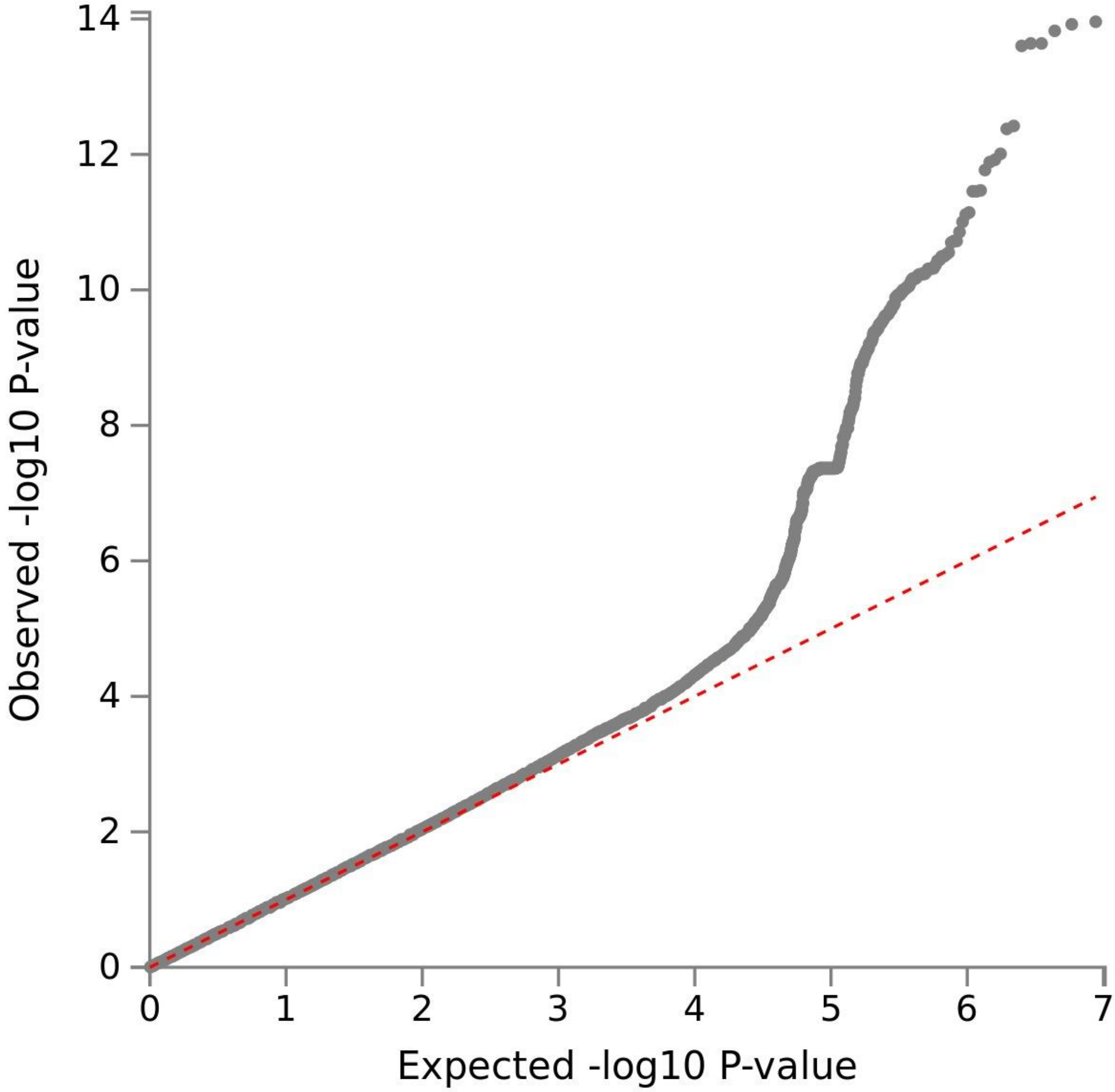

Supplementary Figure 9- Q-Q plot for GWAS of visual network I

Visual network II

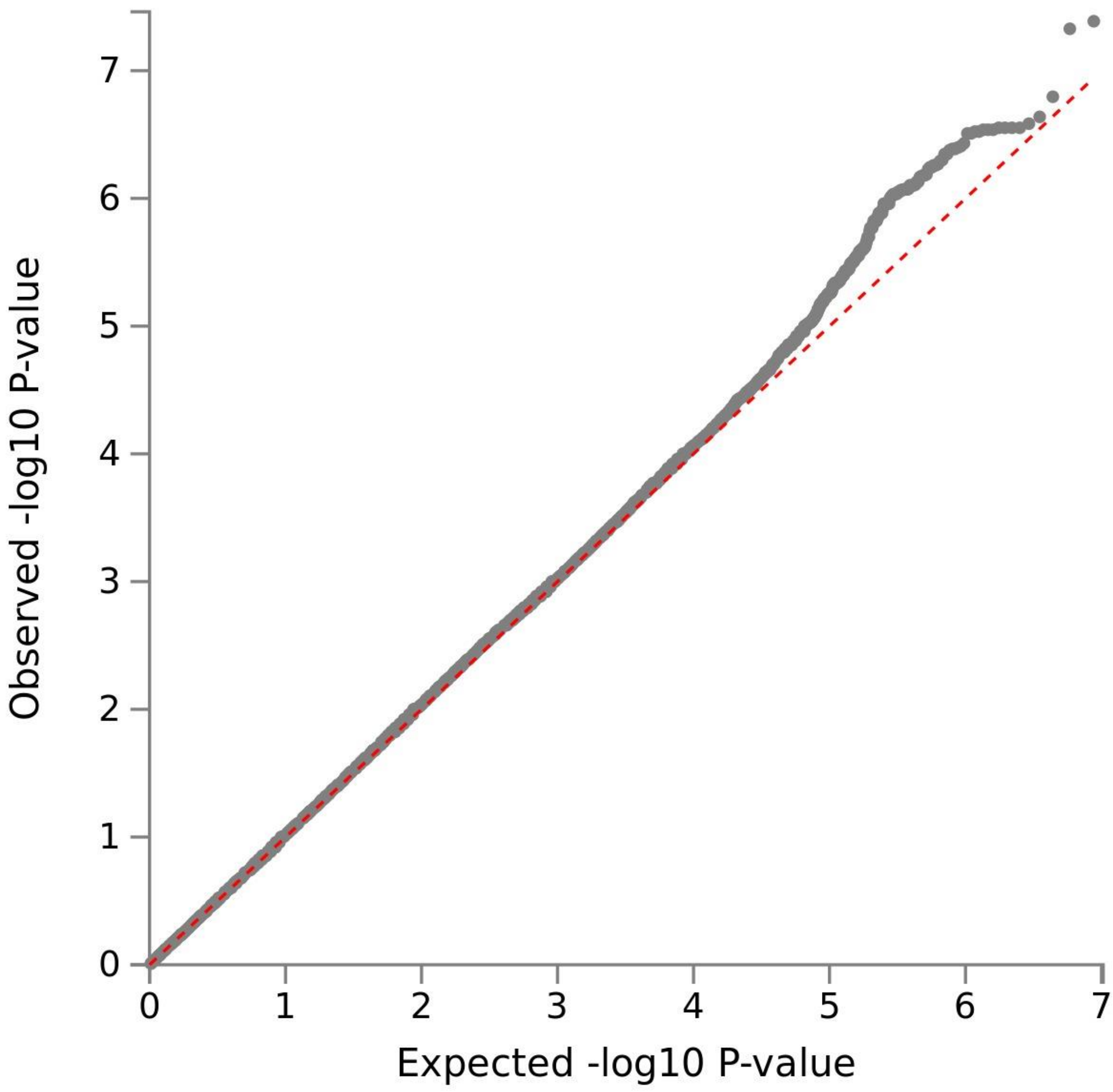

Supplementary Figure 10- Q-Q plot for GWAS of visual network II

Global efficiency adjusted for RSFA

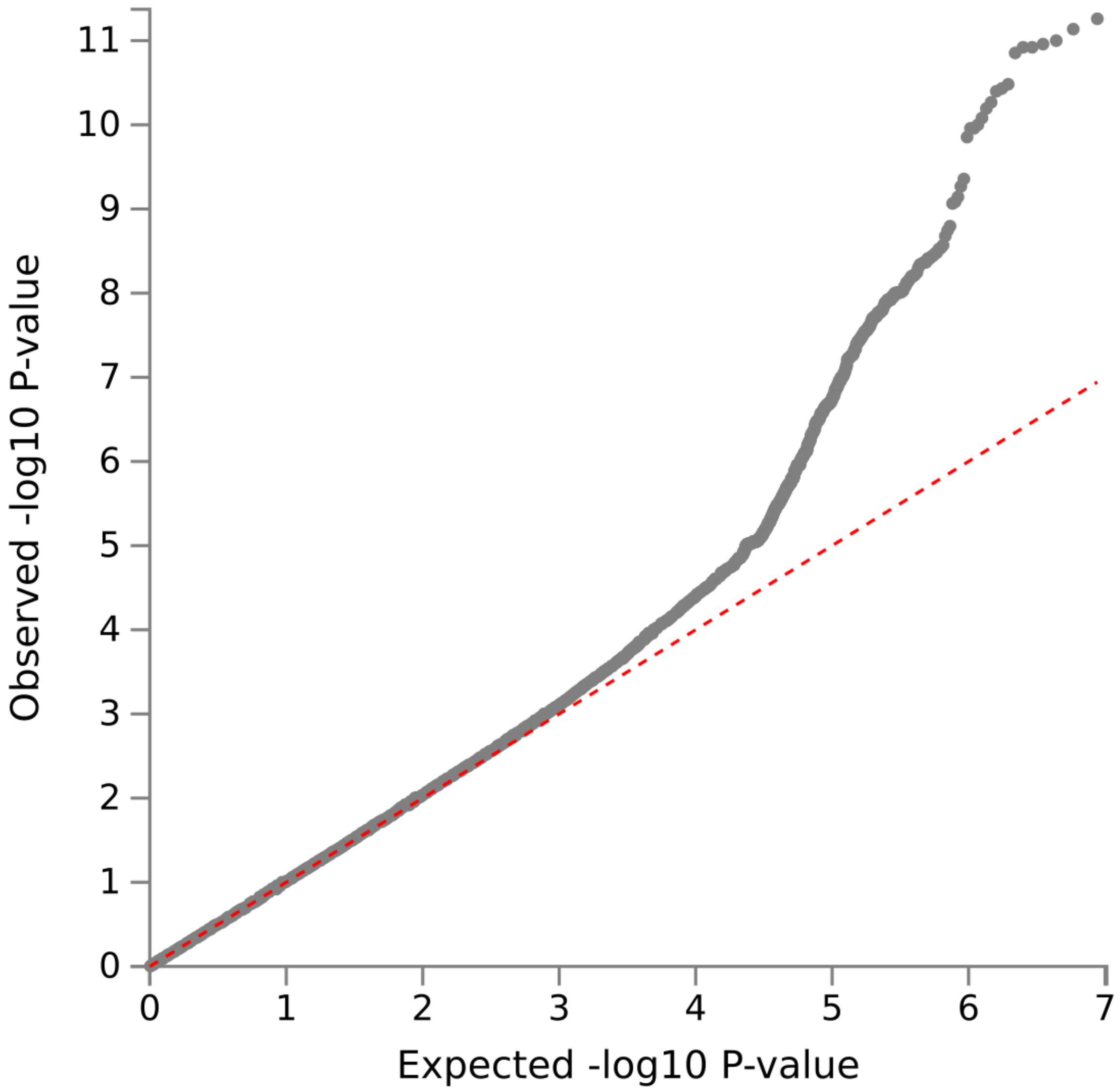

Supplementary Figure 11- Q-Q plot for GWAS of global network efficiency adjusted for RSFA

RSFA

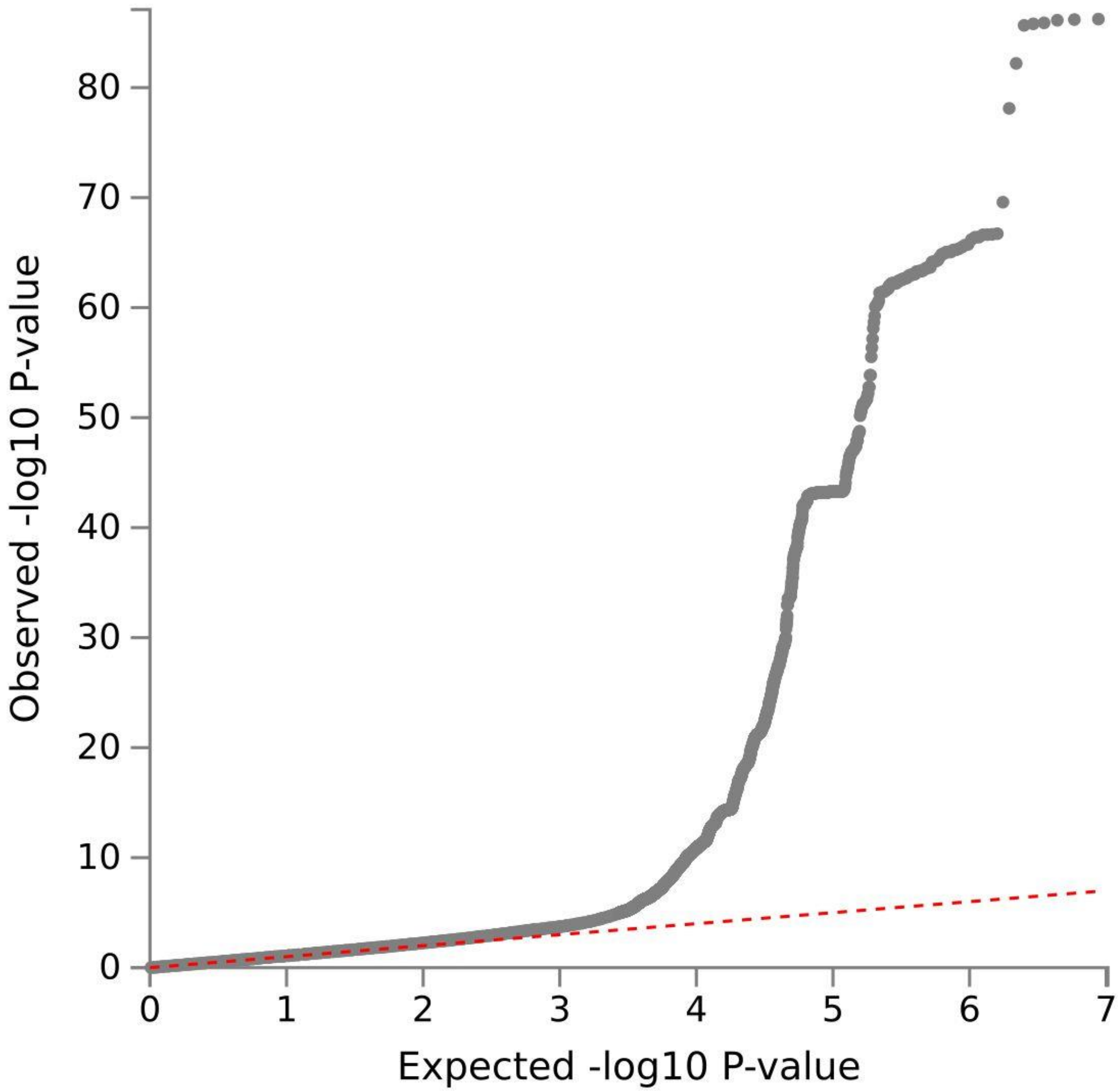

Supplementary Figure 12- Q-Q plot for GWAS of RSFA

Additional adjustments

Systolic blood pressure

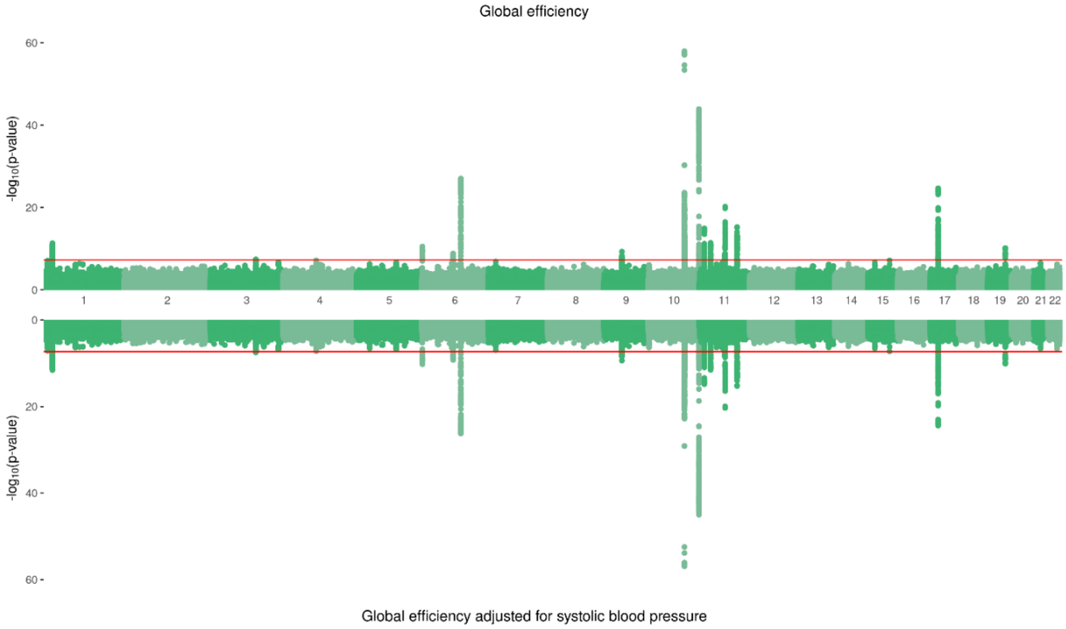

Supplementary Figure 13 - Miami plot of GWAS of global efficiency with (lower) and without (upper) adjustment for systolic blood pressure

Global efficiency and RSFA mutually adjusted

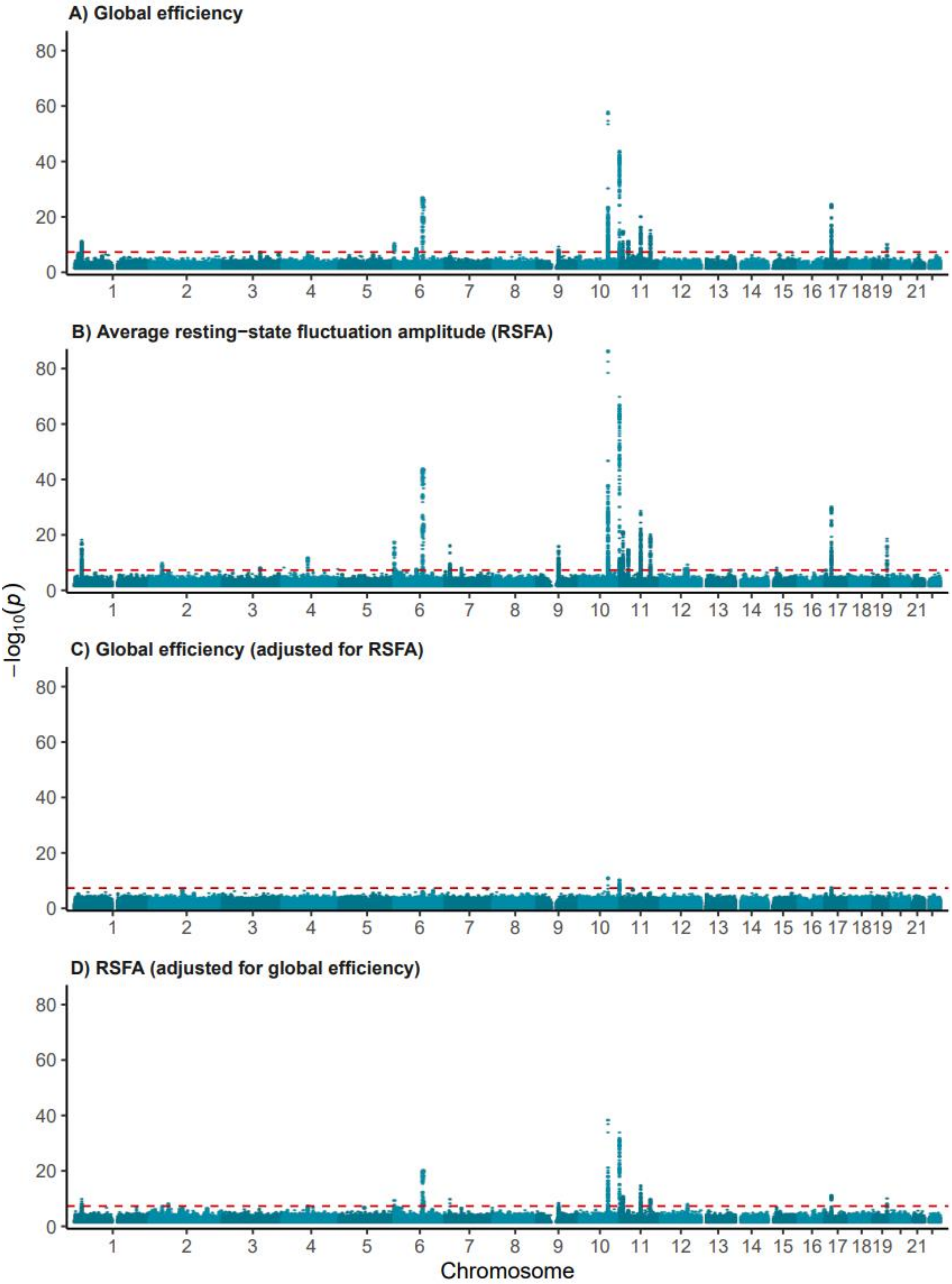

Supplementary Figure 14 - Manhattan plots for global efficiency and resting state fluctuation amplitude with and without adjustment for each other

Regional association plots

Global efficiency - *HSPG2*

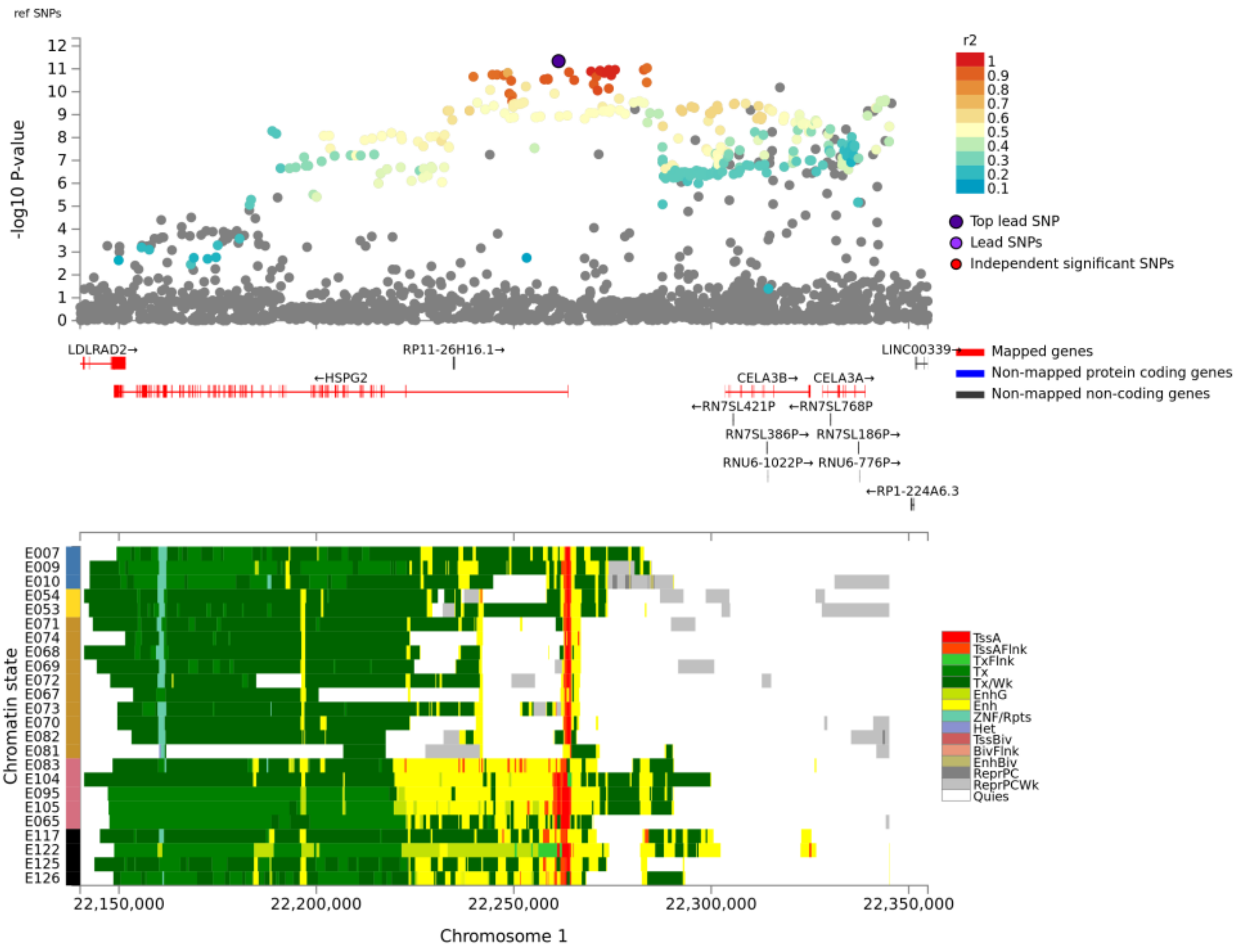

Supplementary Figure 15- Regional association plot with relevant tissue epigenomic data for global efficiency locus HSPG2

Epigenome ID

EID	Color	Group	Anatomy	Standerdized epigenome name
E007	#4178AE	ES-deriv	ESC_DERIVED	H1 Derived Neuronal Progenitor Cultured Cells
E009	#4178AE	ES-deriv	ESC_DERIVED	H9 Derived Neuronal Progenitor Cultured Cells
E010	#4178AE	ES-deriv	ESC_DERIVED	H9 Derived Neuron Cultured Cells
E054	#FFD924	Neurosph	BRAIN	Ganglion Eminence derived primary cultured neurospheres
E053	#FFD924	Neurosph	BRAIN	Cortex derived primary cultured neurospheres
E071	#C5912B	Brain	BRAIN	Brain Hippocampus Middle
E074	#C5912B	Brain	BRAIN	Brain Substantia Nigra
E068	#C5912B	Brain	BRAIN	Brain Anterior Caudate
E069	#C5912B	Brain	BRAIN	Brain Cingulate Gyrus
E072	#C5912B	Brain	BRAIN	Brain Inferior Temporal Lobe
E067	#C5912B	Brain	BRAIN	Brain Angular Gyrus
E073	#C5912B	Brain	BRAIN	Brain_Dorsolateral_Prefrontal_Cortex
E070	#C5912B	Brain	BRAIN	Brain Germinal Matrix
E082	#C5912B	Brain	BRAIN	Fetal Brain Female
E081	#C5912B	Brain	BRAIN	Fetal Brain Male
E083	#D56F80	Heart	HEART	Fetal Heart
E104	#D56F80	Heart	HEART	Right Atrium
E095	#D56F80	Heart	HEART	Left Ventricle
E105	#D56F80	Heart	HEART	Right Ventricle
E065	#D56F80	Heart	VASCULAR	Aorta
E117	#000000	ENCODE2012	CERVIX	HeLa-S3 Cervical Carcinoma Cell Line
E122	#000000	ENCODE2012	VASCULAR	HUVEC Umbilical Vein Endothelial Primary Cells
E125	#000000	ENCODE2012	BRAIN	NH-A Astrocytes Primary Cells
E126	#000000	ENCODE2012	SKIN	NHDF-Ad Adult Dermal Fibroblast Primary Cells

Global efficiency - *ITGB5*

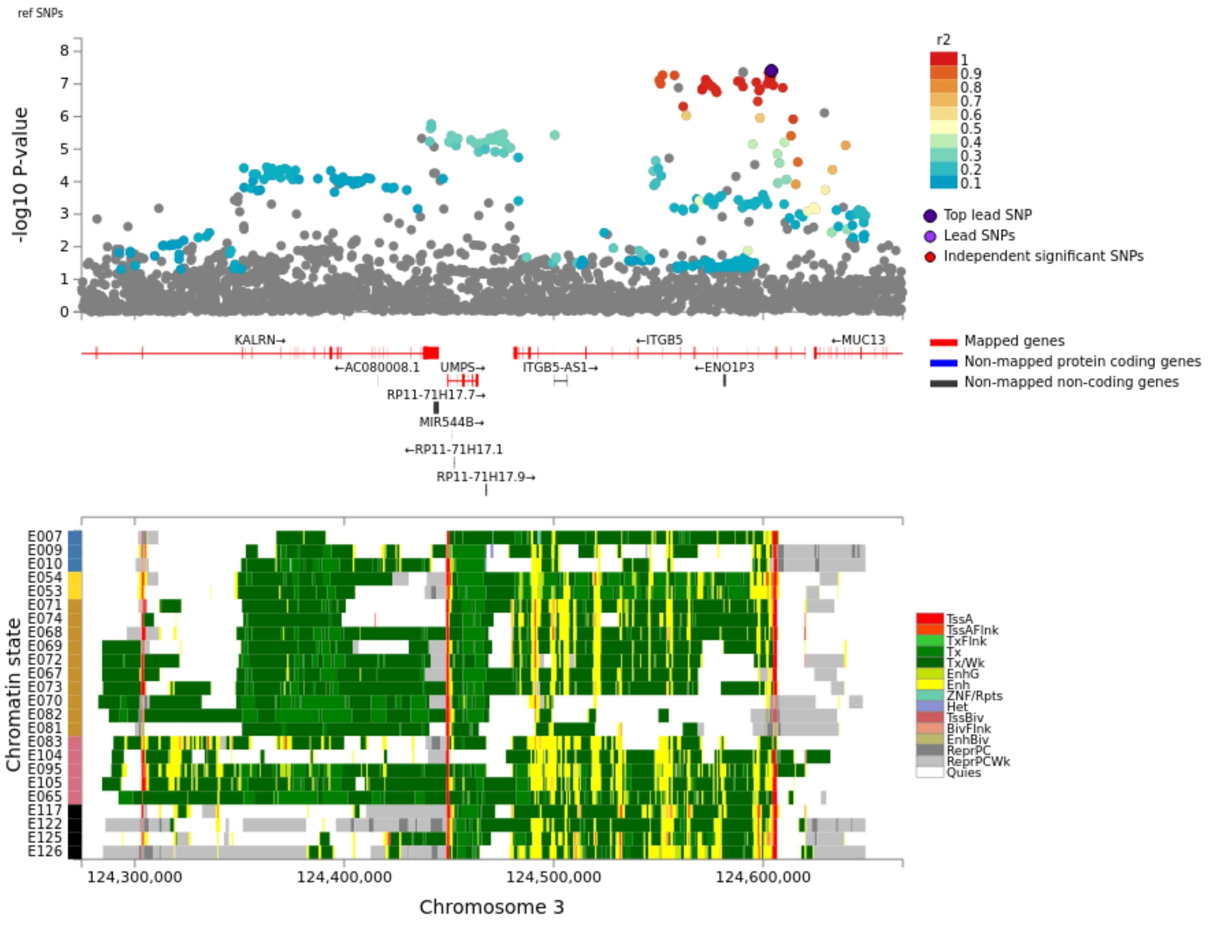

Supplementary Figure 16- Regional association plot with relevant tissue epigenomic data for global efficiency locus ITGB5

Global efficiency - *FOXQ1*

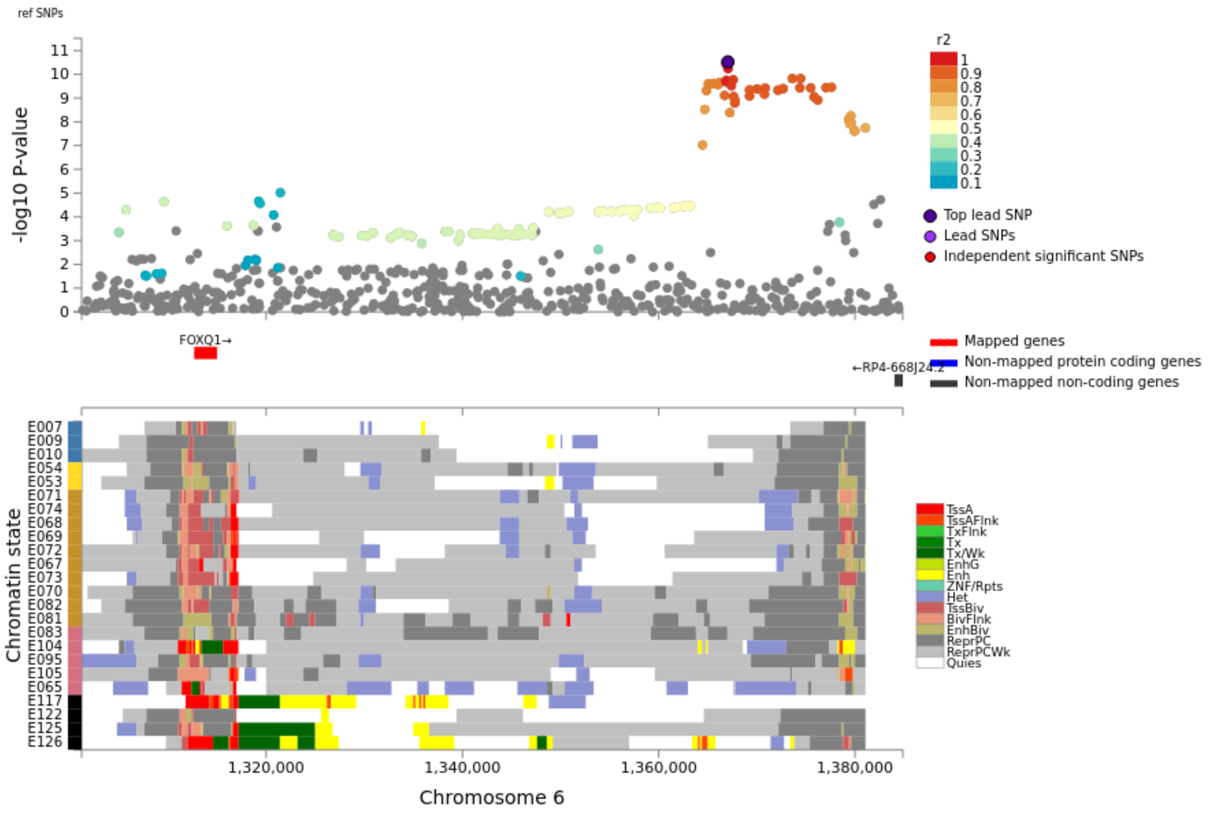

Supplementary Figure 17- Regional association plot with relevant tissue epigenomic data for global efficiency locus FOXQ1

Global efficiency – *RP11-474L11.3-6ENP6*

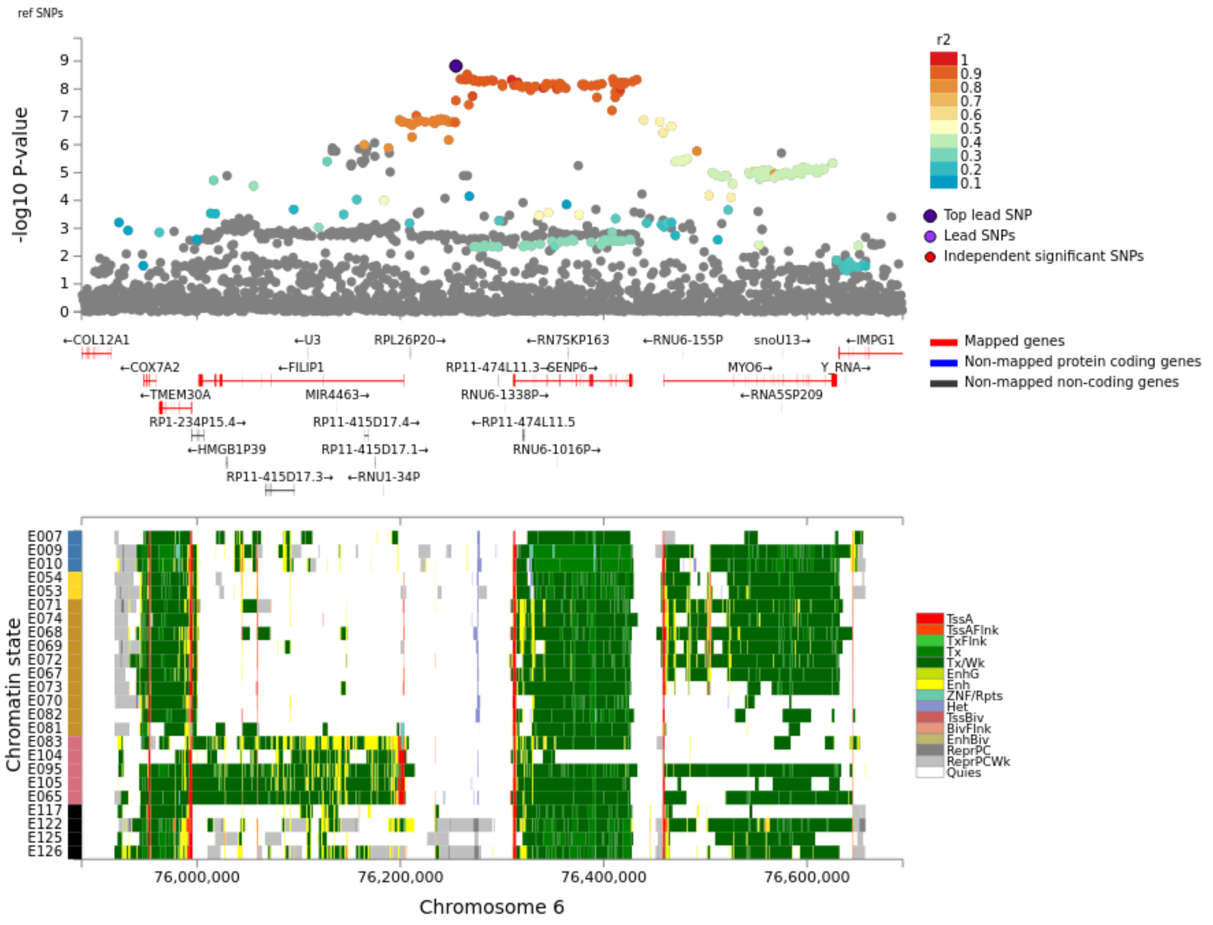

Supplementary Figure 18- Regional association plot with relevant tissue epigenomic data for global efficiency locus RP11-474L11.3-6ENP6

Global efficiency - *UFL1*

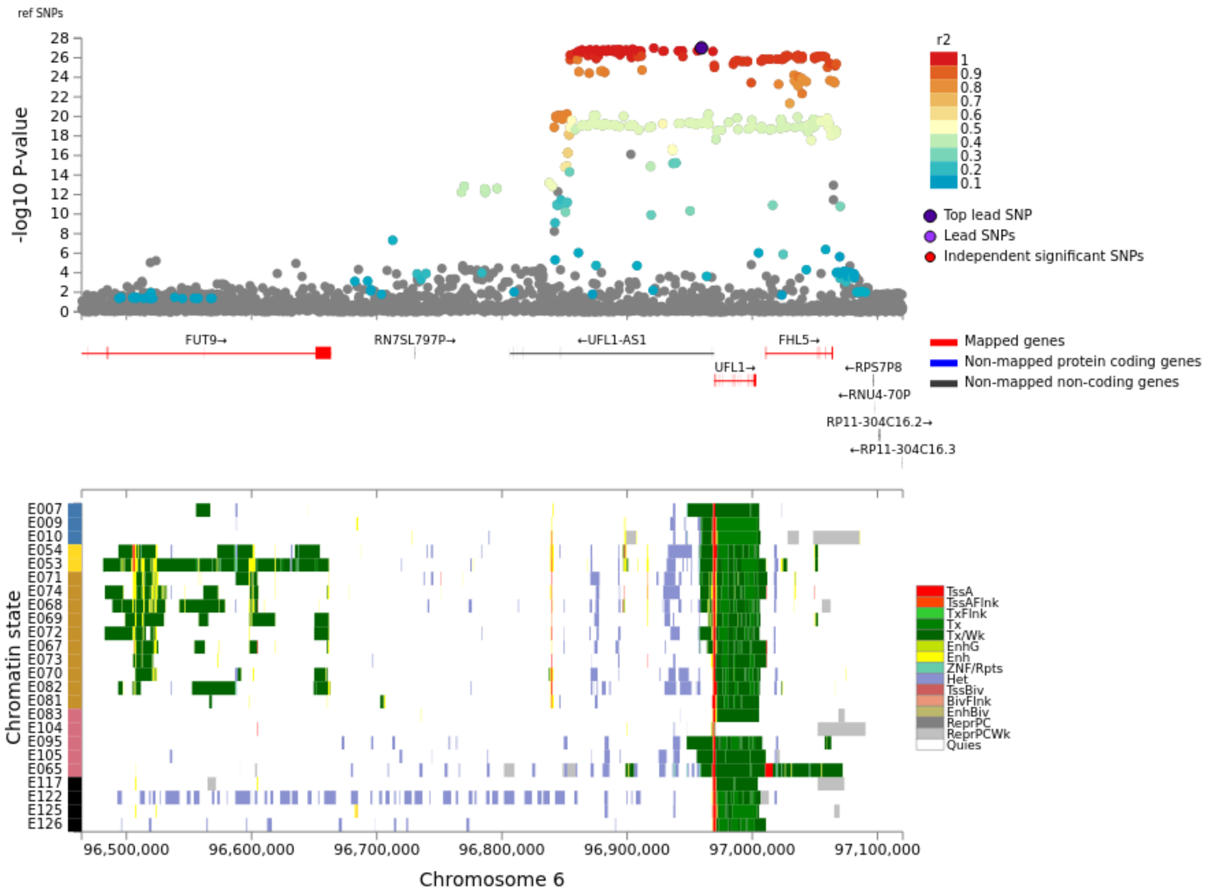

Supplementary Figure 19- Regional association plot with relevant tissue epigenomic data for global efficiency locus UFL1

Global efficiency - *PIP5K1B*

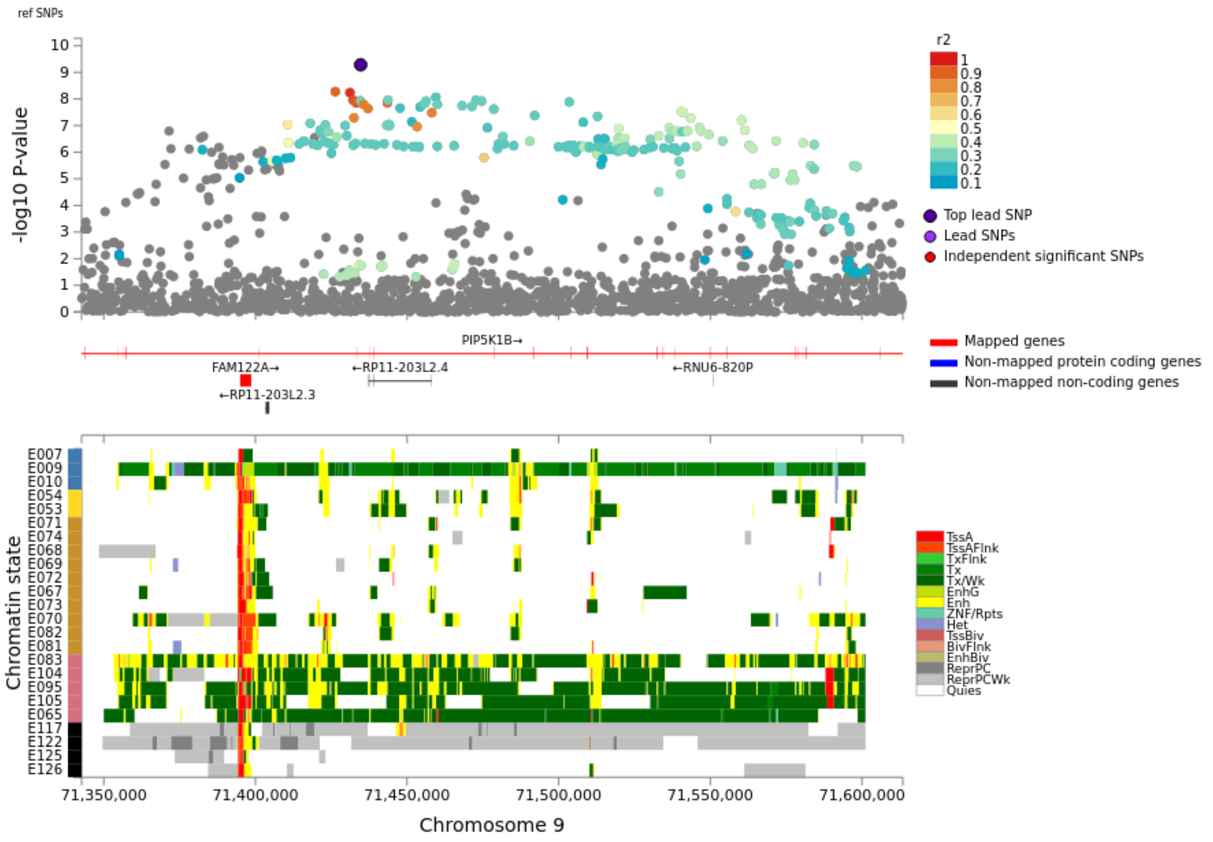

Supplementary Figure 20- Regional association plot with relevant tissue epigenomic data for global efficiency locus PIP5K18

Global efficiency - *PLCE1*

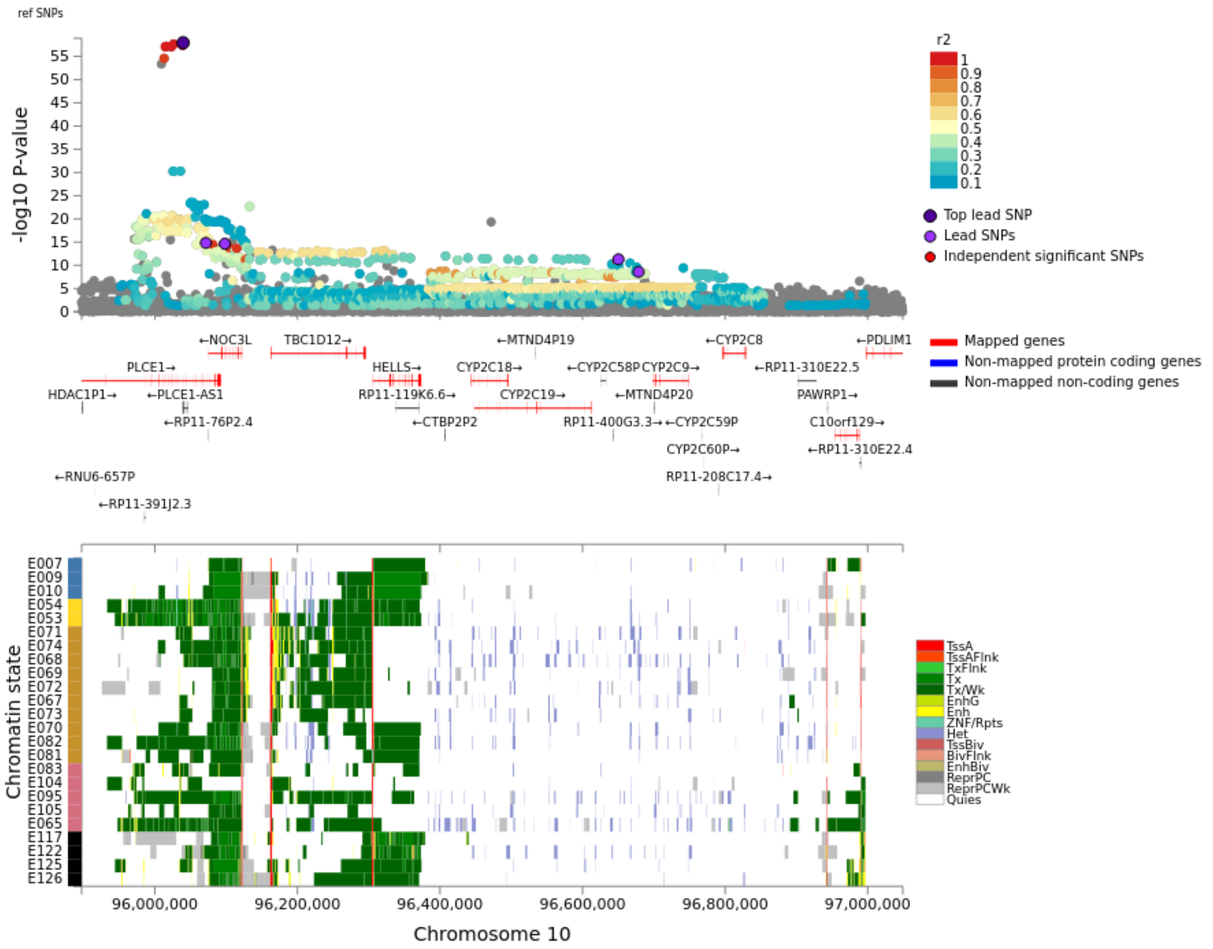

Supplementary Figure 21- Regional association plot with relevant tissue epigenomic data for global efficiency locus PLCE1

Global efficiency - *C10orf91/INPP5A*

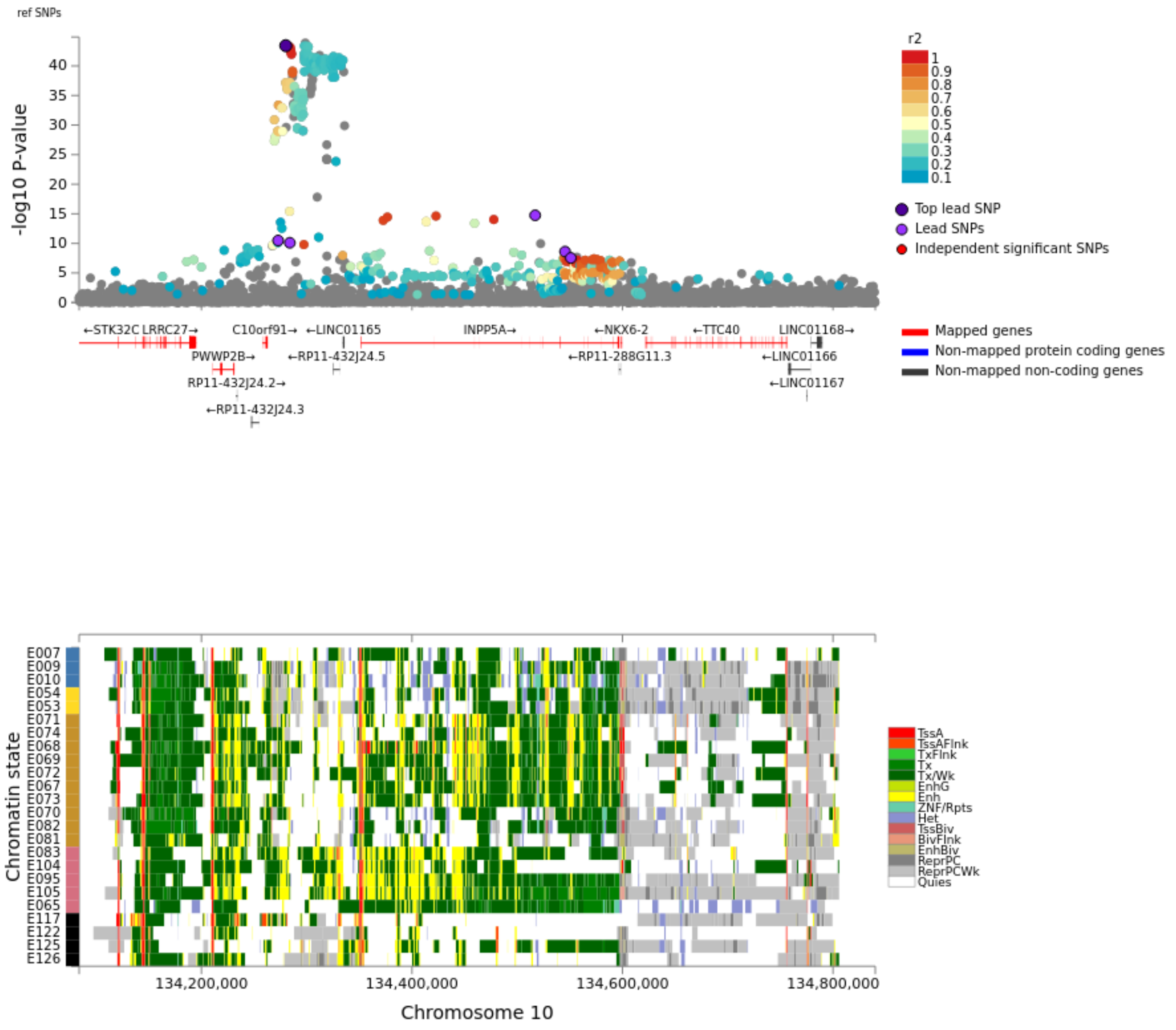

Supplementary Figure 22- Regional association plot with relevant tissue epigenomic data for global efficiency locus C10orf91/INPP5A

Global efficiency - *MRVI1*

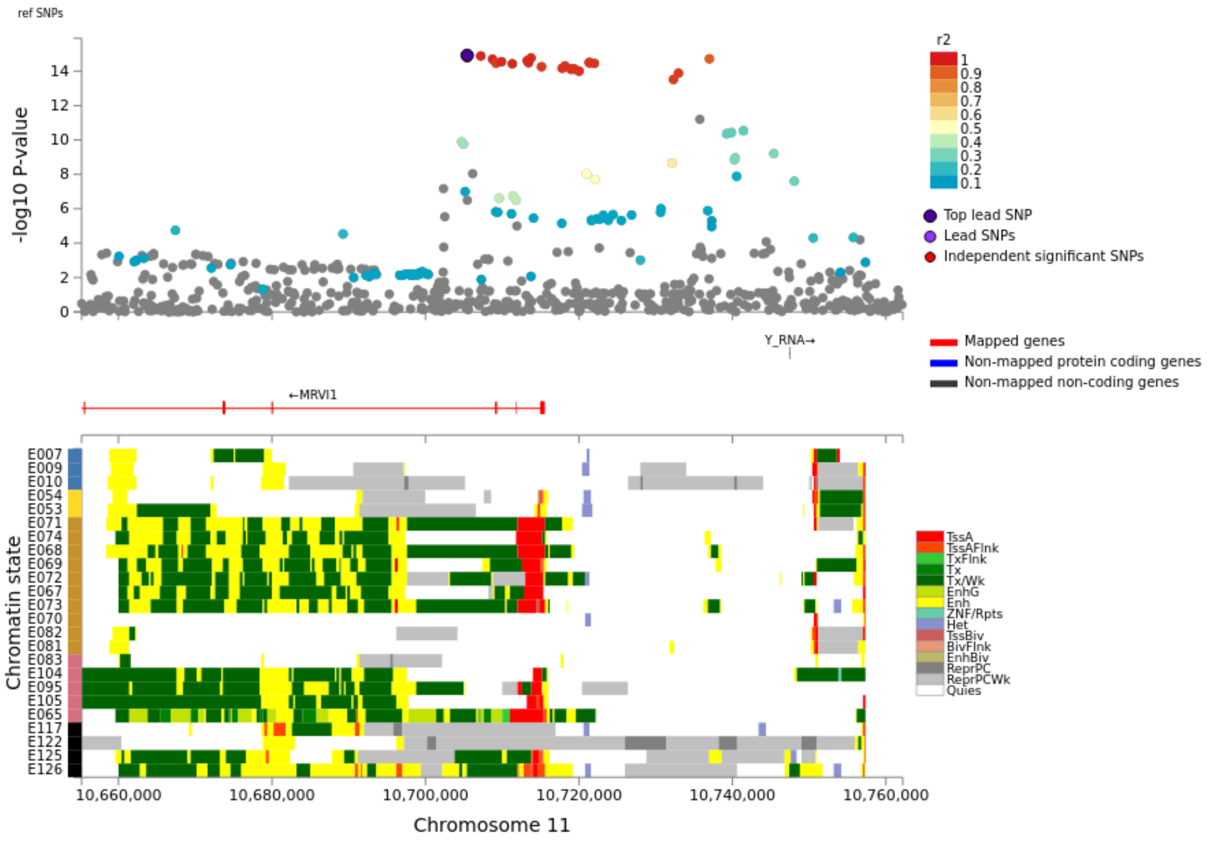

Supplementary Figure 23- Regional association plot with relevant tissue epigenomic data for global efficiency locus MRVI1

Global efficiency - *ANO3*

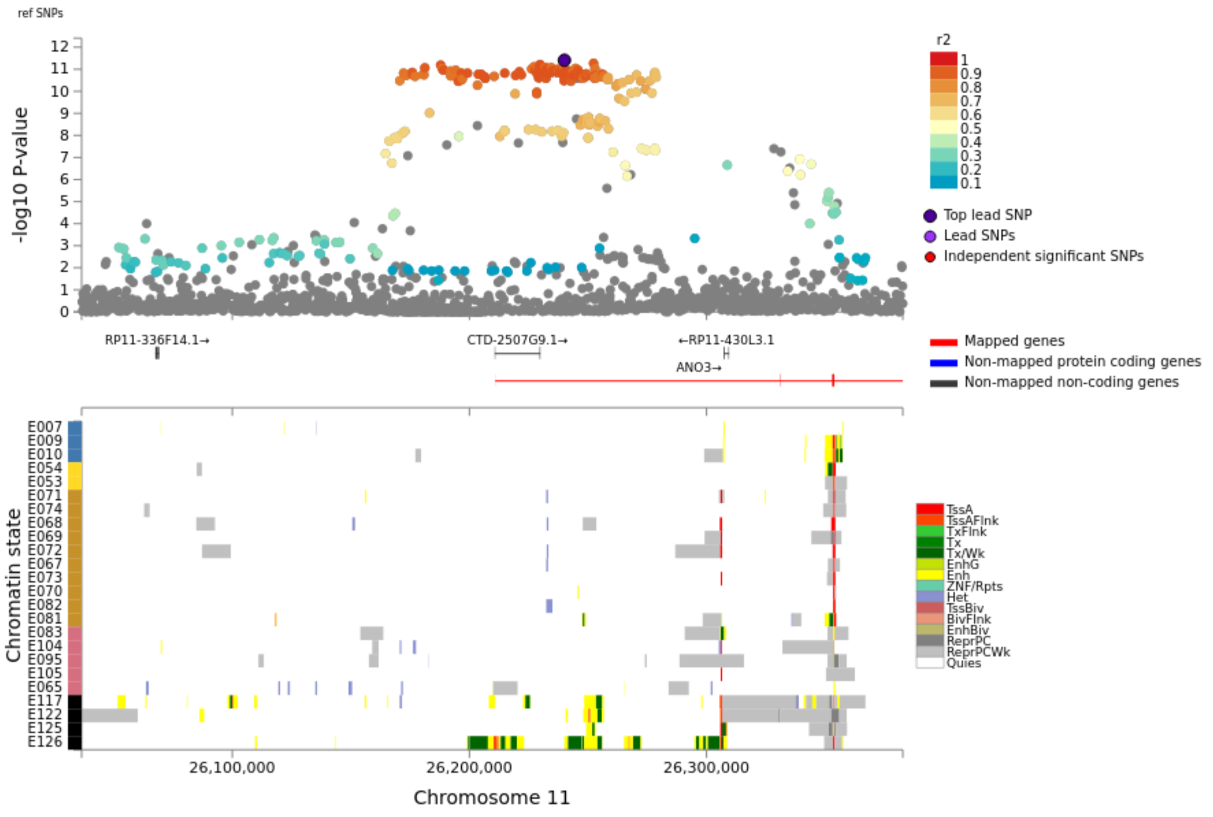

Supplementary Figure 24- Regional association plot with relevant tissue epigenomic data for global efficiency locus ANO3

Global efficiency - *ANO1*

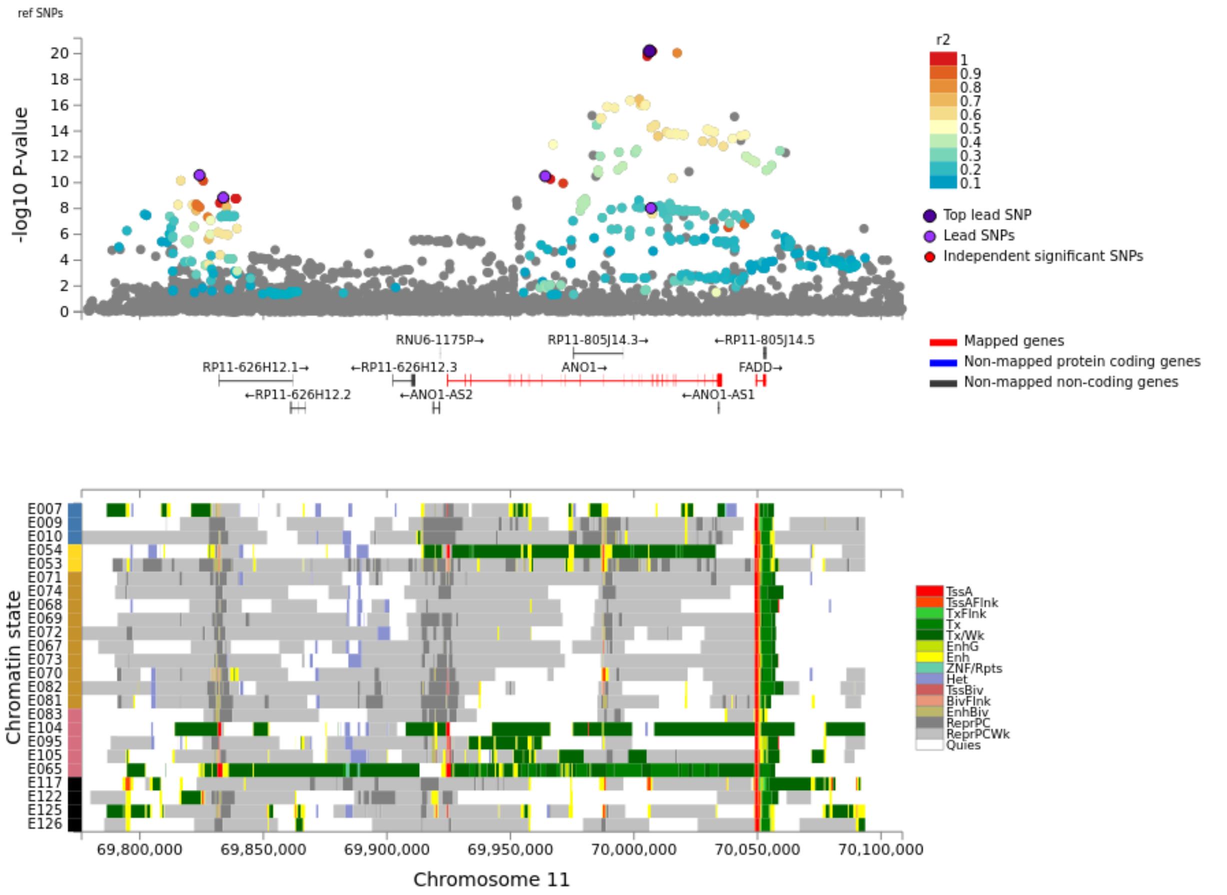

Supplementary Figure 25- Regional association plot with relevant tissue epigenomic data for global efficiency locus ANO1

Global efficiency - *TRPC6*

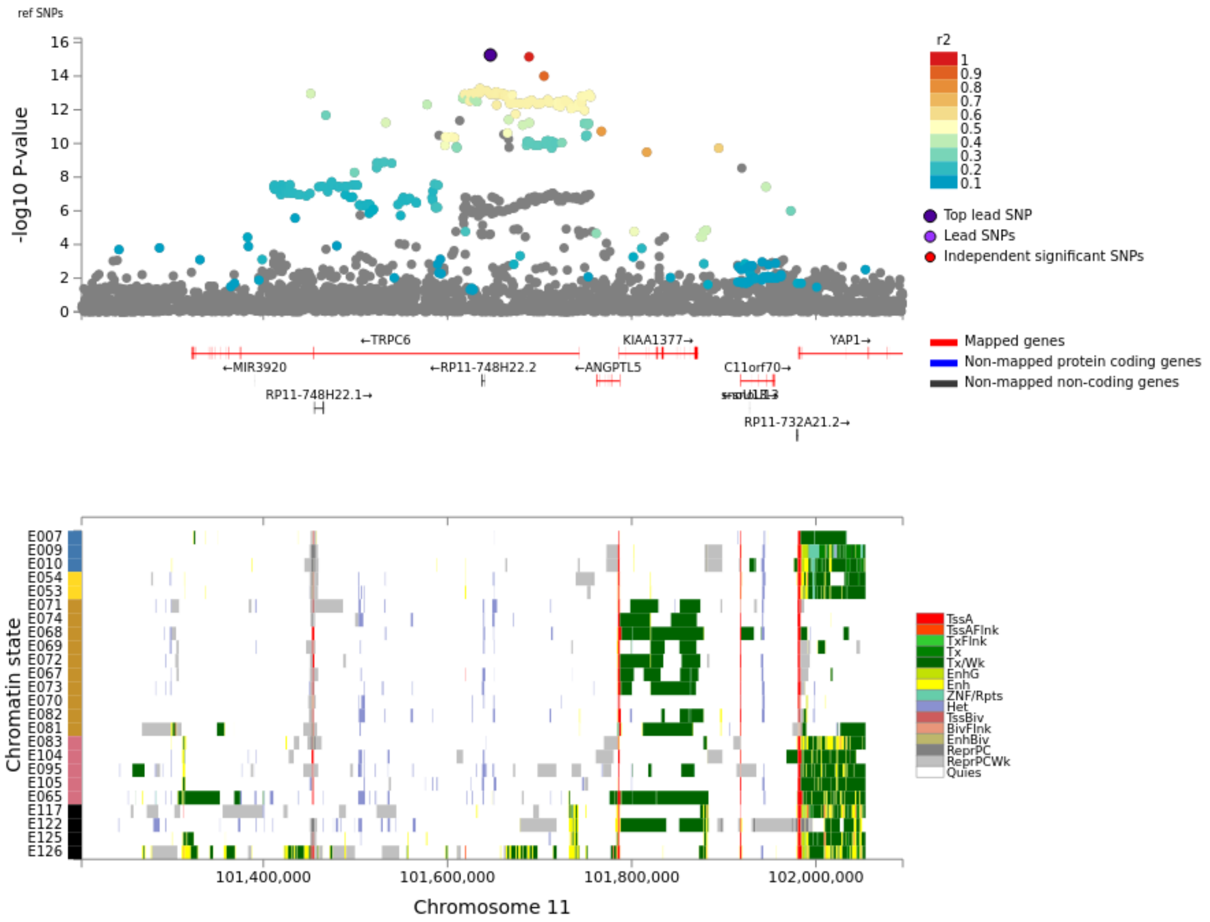

Supplementary Figure 26- Regional association plot with relevant tissue epigenomic data for global efficiency locus TRPC6

Global efficiency - *EPN2*

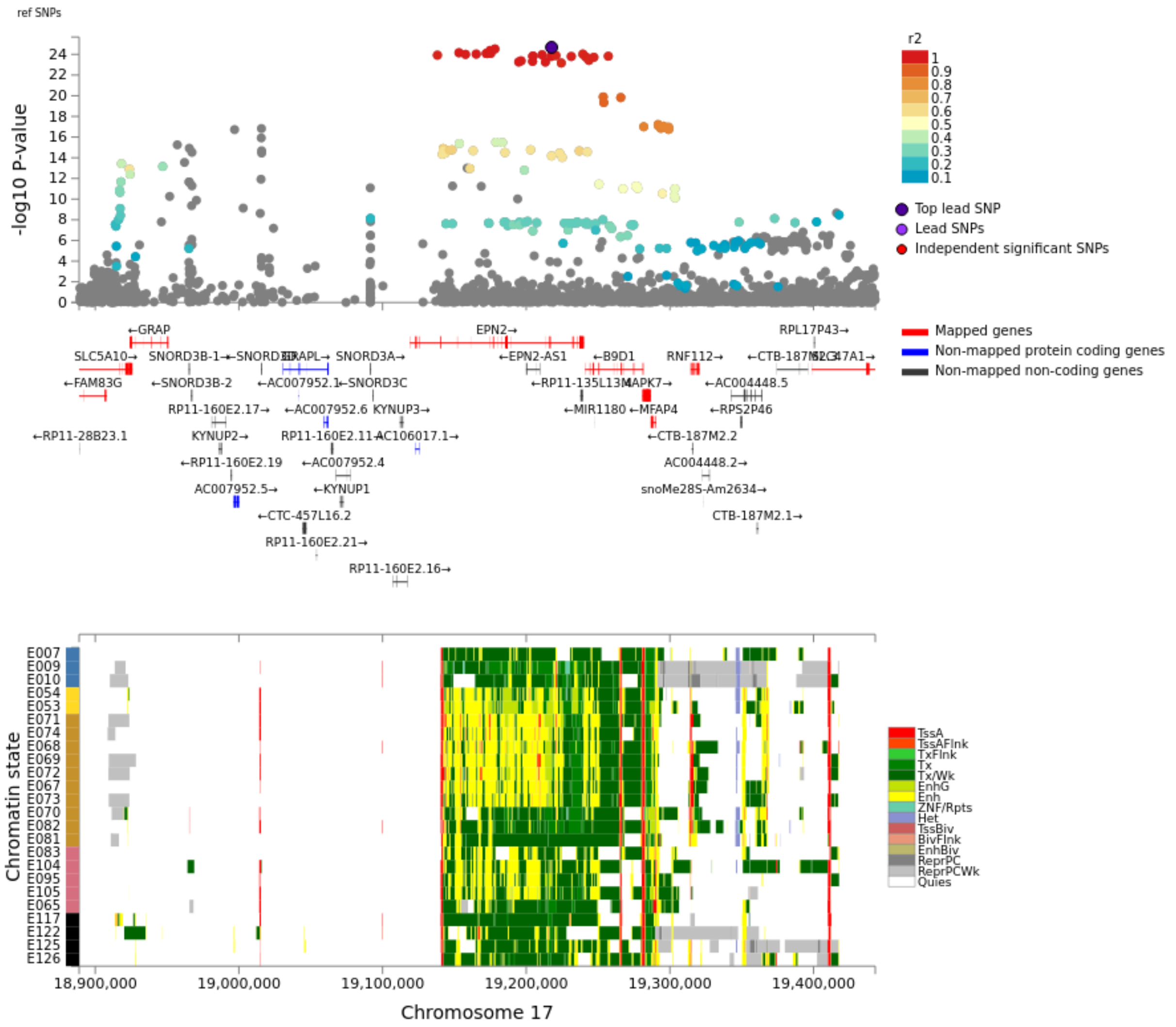

Supplementary Figure 27- Regional association plot with relevant tissue epigenomic data for global efficiency locus EPN2

Global efficiency - *APOE*

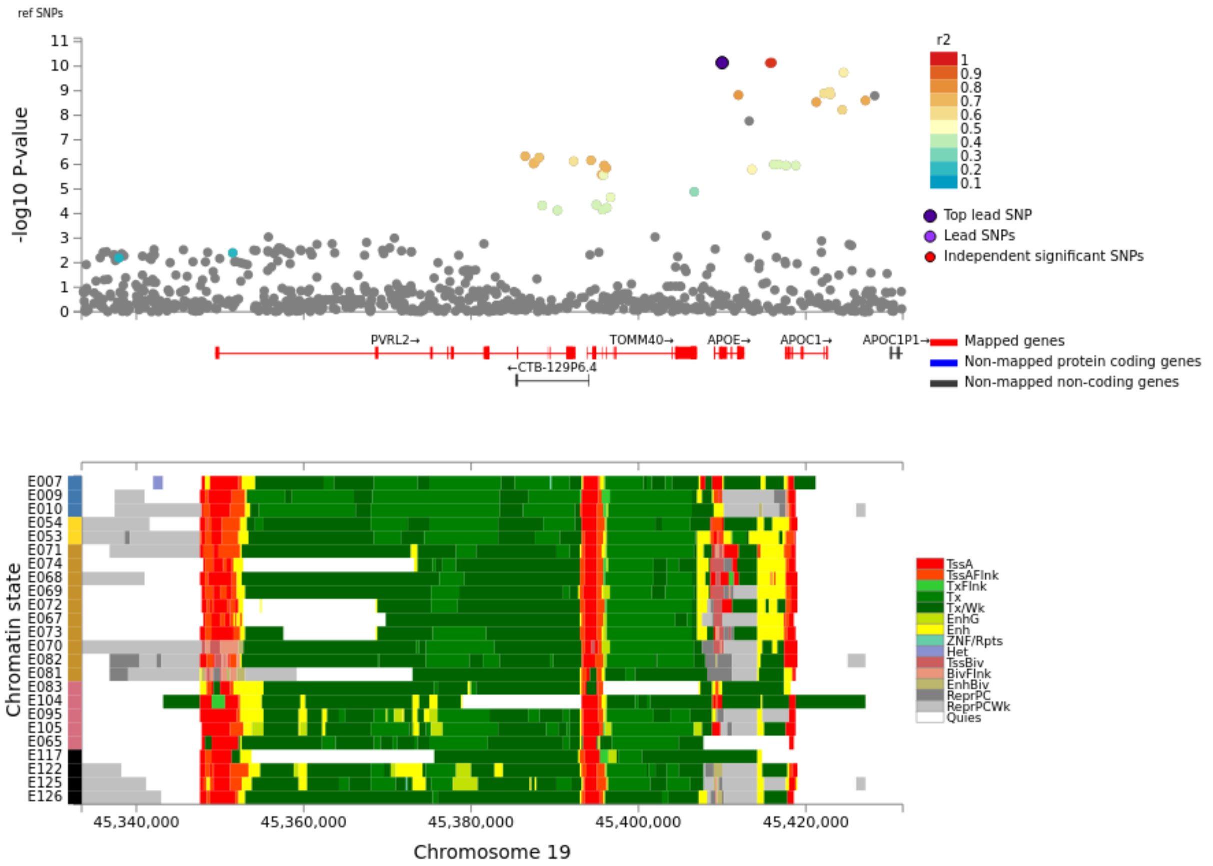

Supplementary Figure 28- Regional association plot with relevant tissue epigenomic data for global efficiency locus APOE

Local efficiency - *GRIK2*

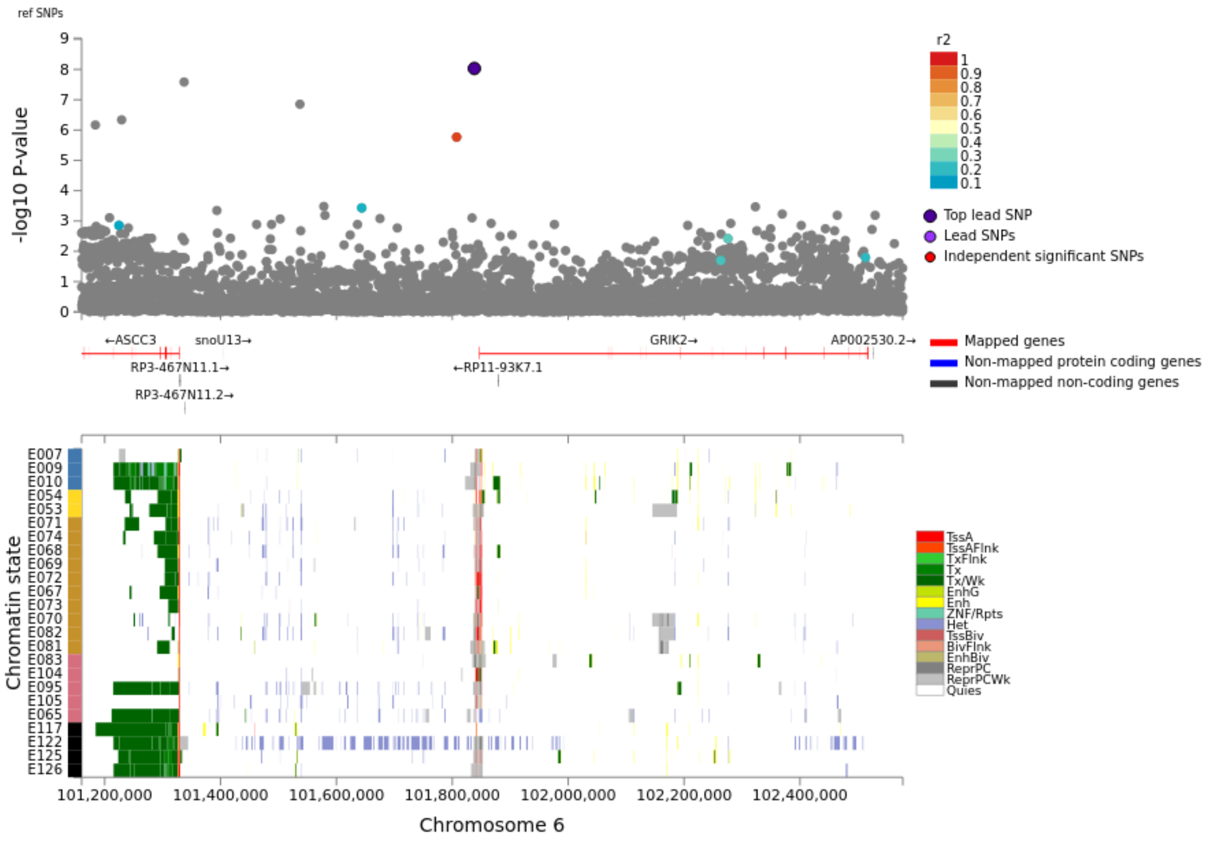

Supplementary Figure 29- Regional association plot with relevant tissue epigenomic data for local efficiency locus GRIK2

Motor network - *EPHA3*

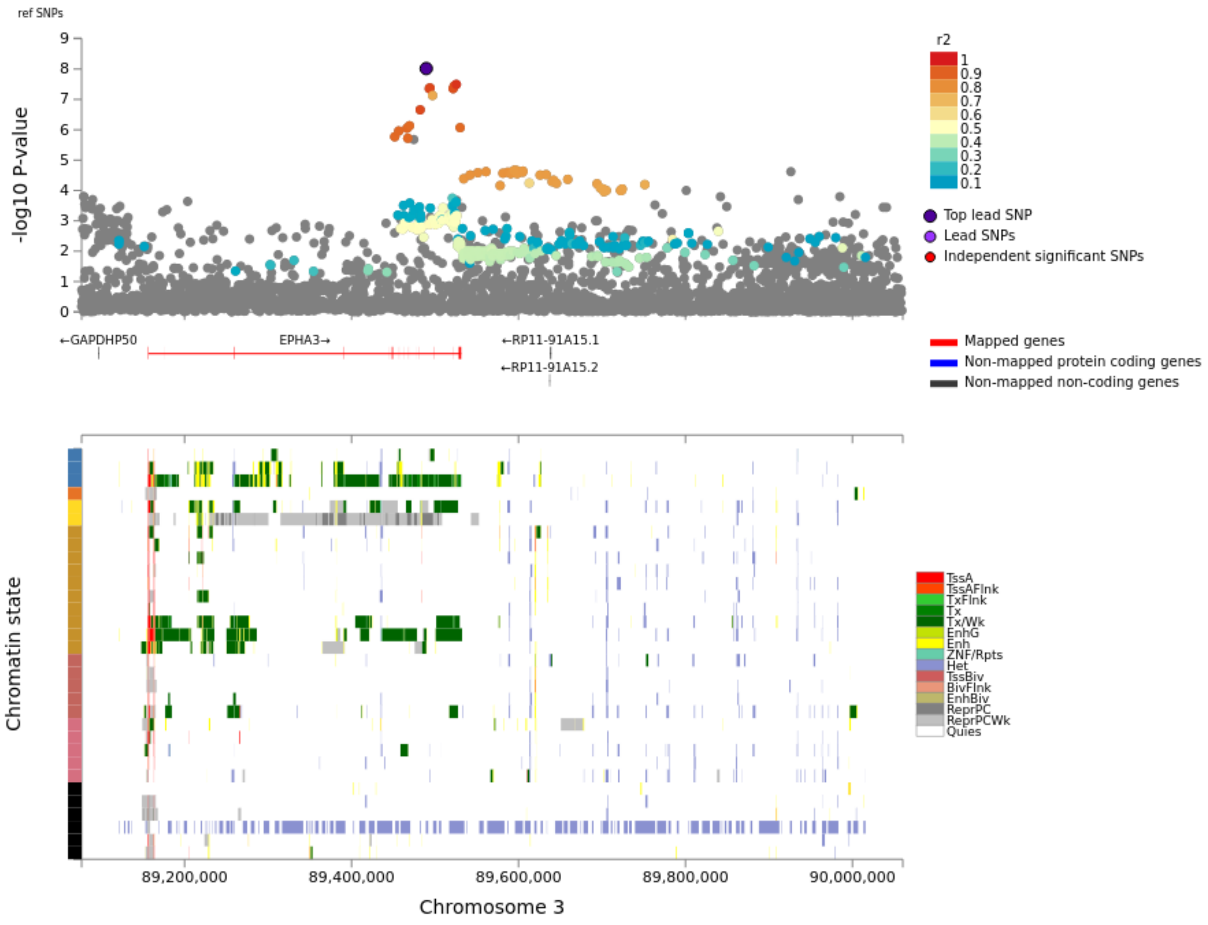

Supplementary Figure 30- Regional association plot with relevant tissue epigenomic data for motor network locus EPHA3

Epigenome ID

EID	Color	Group	Anatomy	Standerdized epigenome name
E007	#4178AE	ES-deriv	ESC_DERIVED	H1 Derived Neuronal Progenitor Cultured Cells
E009	#4178AE	ES-deriv	ESC_DERIVED	H9 Derived Neuronal Progenitor Cultured Cells
E010	#4178AE	ES-deriv	ESC_DERIVED	H9 Derived Neuron Cultured Cells
E052	#E67326	Myosat	MUSCLE	Muscle Satellite Cultured Cells
E054	#FFD924	Neurosph	BRAIN	Ganglion Eminence derived primary cultured neurospheres
E053	#FFD924	Neurosph	BRAIN	Cortex derived primary cultured neurospheres
E071	#C5912B	Brain	BRAIN	Brain Hippocampus Middle
E074	#C5912B	Brain	BRAIN	Brain Substantia Nigra
E068	#C5912B	Brain	BRAIN	Brain Anterior Caudate
E069	#C5912B	Brain	BRAIN	Brain Cingulate Gyrus
E072	#C5912B	Brain	BRAIN	Brain Inferior Temporal Lobe
E067	#C5912B	Brain	BRAIN	Brain Angular Gyrus
E073	#C5912B	Brain	BRAIN	Brain_Dorsolateral_Prefrontal_Cortex
E070	#C5912B	Brain	BRAIN	Brain Germinal Matrix
E082	#C5912B	Brain	BRAIN	Fetal Brain Female
E081	#C5912B	Brain	BRAIN	Fetal Brain Male
E100	#C2655D	Muscle	MUSCLE	Psoas Muscle
E108	#C2655D	Muscle	MUSCLE	Skeletal Muscle Female
E107	#C2655D	Muscle	MUSCLE	Skeletal Muscle Male
E089	#C2655D	Muscle	MUSCLE	Fetal Muscle Trunk
E090	#C2655D	Muscle	MUSCLE_LEG	Fetal Muscle Leg
E083	#D56F80	Heart	HEART	Fetal Heart
E104	#D56F80	Heart	HEART	Right Atrium
E095	#D56F80	Heart	HEART	Left Ventricle
E105	#D56F80	Heart	HEART	Right Ventricle
E065	#D56F80	Heart	VASCULAR	Aorta
E117	#000000	ENCODE2012	CERVIX	HeLa-S3 Cervical Carcinoma Cell Line
E120	#000000	ENCODE2012	MUSCLE	HSMM Skeletal Muscle Myoblasts Cells
E121	#000000	ENCODE2012	MUSCLE	HSMM cell derived Skeletal Muscle Myotubes Cells
E122	#000000	ENCODE2012	VASCULAR	HUVEC Umbilical Vein Endothelial Primary Cells
E125	#000000	ENCODE2012	BRAIN	NH-A Astrocytes Primary Cells
E126	#000000	ENCODE2012	SKIN	NHDF-Ad Adult Dermal Fibroblast Primary Cells

Motor network - *FAM3C*

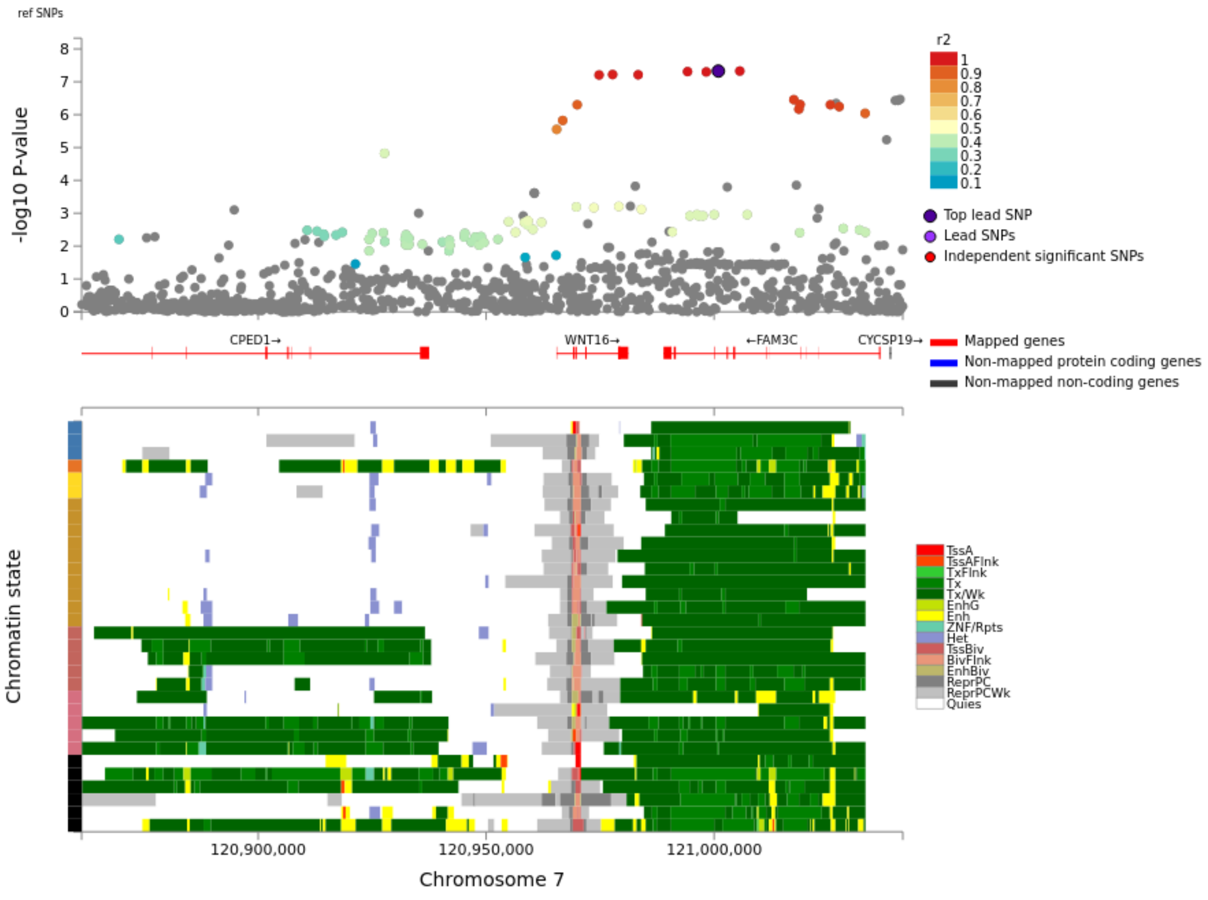

Supplementary Figure 31- Regional association plot with relevant tissue epigenomic data for motor network locus FAM3C

Subcortical-cerebellum network - *KCND2*

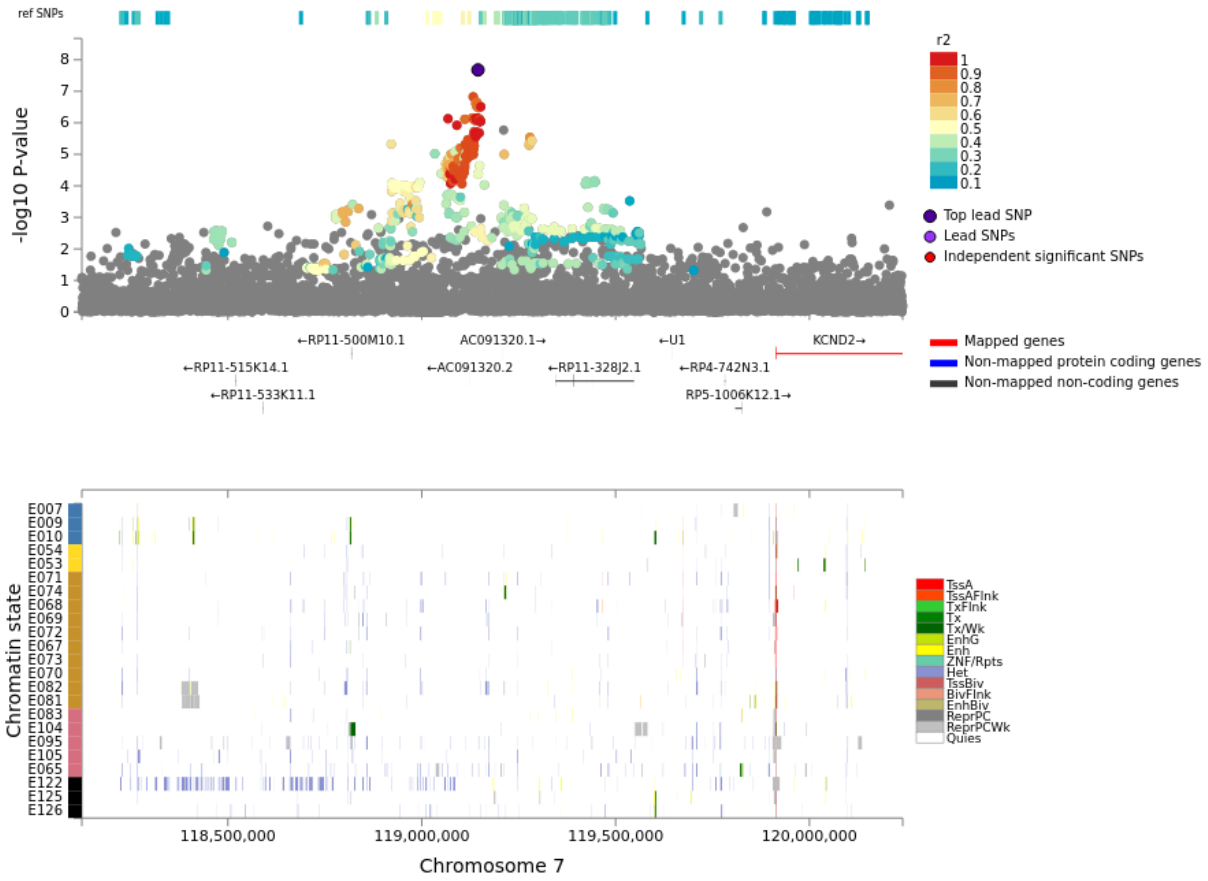

Supplementary Figure 32- Regional association plot with relevant tissue epigenomic data for subcortical-cerebellum network locus KCND2

Subcortical-cerebellum network - *WNT16*

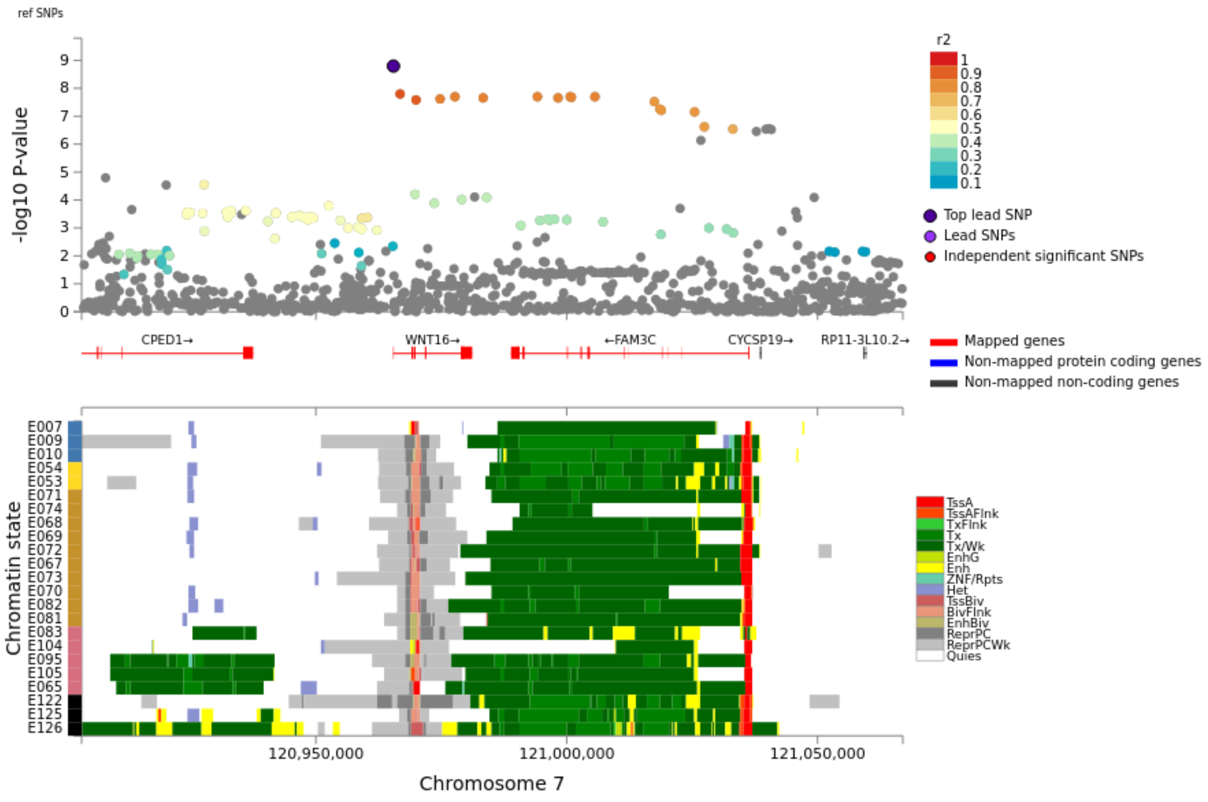

Supplementary Figure 33- Regional association plot with relevant tissue epigenomic data for subcortical-cerebellum network locus WNT16

Visual network I - *UFL1*

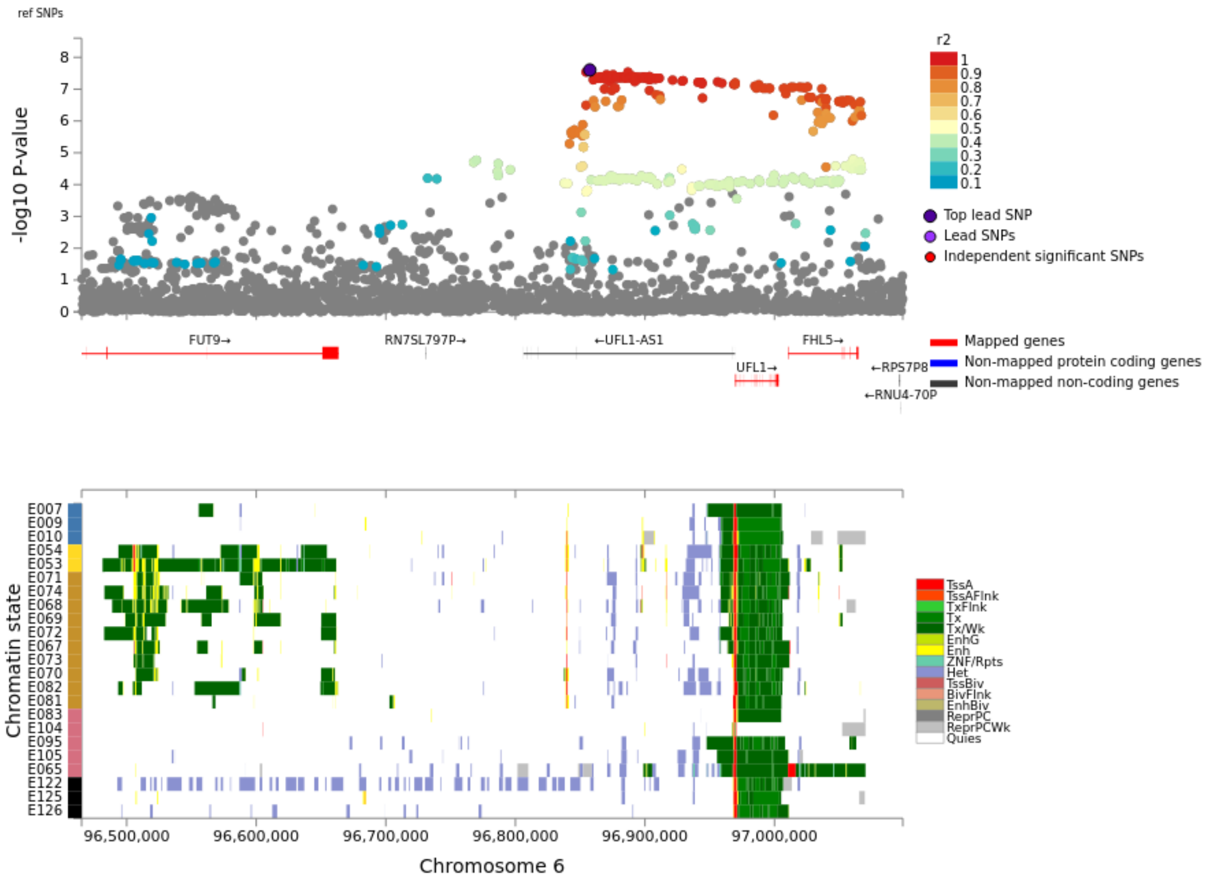

Supplementary Figure 34- Regional association plot with relevant tissue epigenomic data for visual network I locus UFL1

Visual network 1 - *PLCE1*

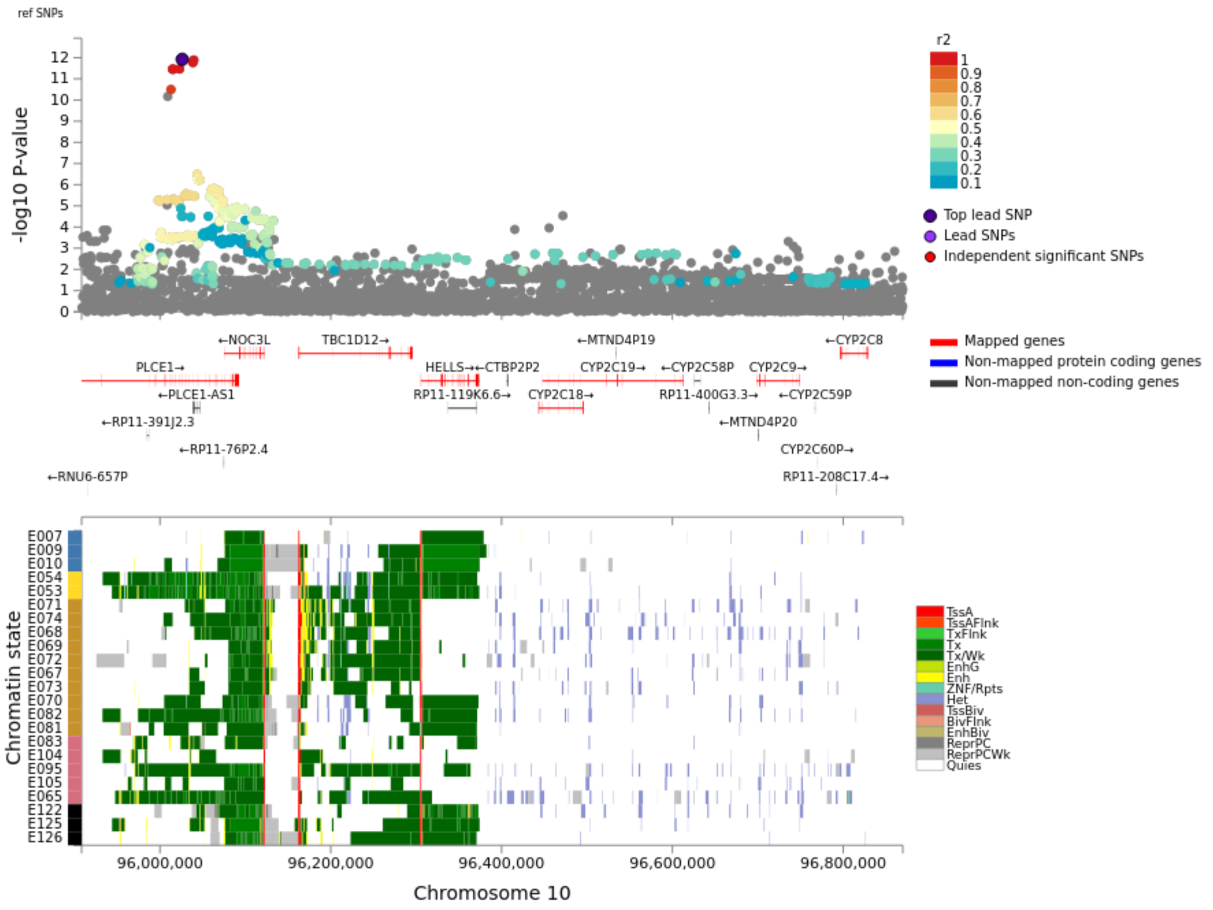

Supplementary Figure 35- Regional association plot with relevant tissue epigenomic data for visual network 1 locus PLCE1

Visual network I - *C10orf91*

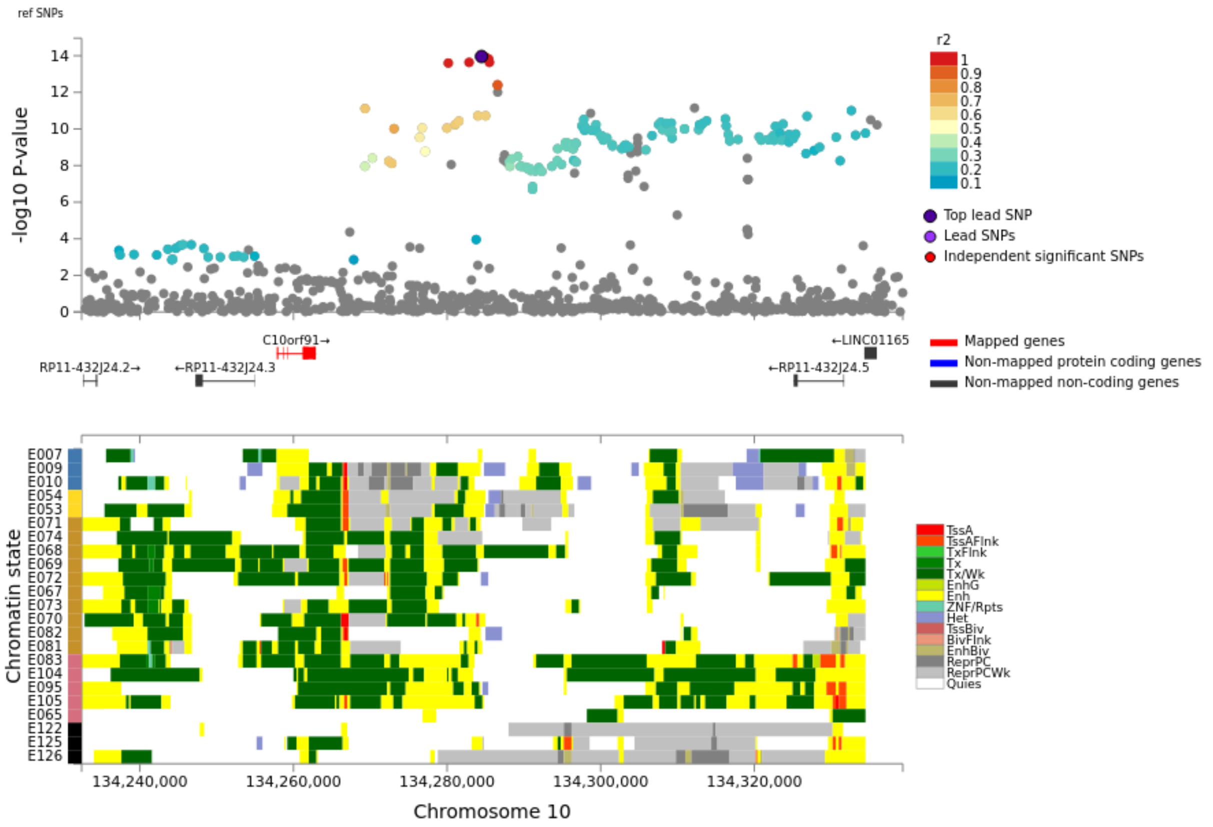

Supplementary Figure 36- Regional association plot with relevant tissue epigenomic data for visual network I locus C10orf91

Global efficiency adjusted for RSFA – *PLCE1*

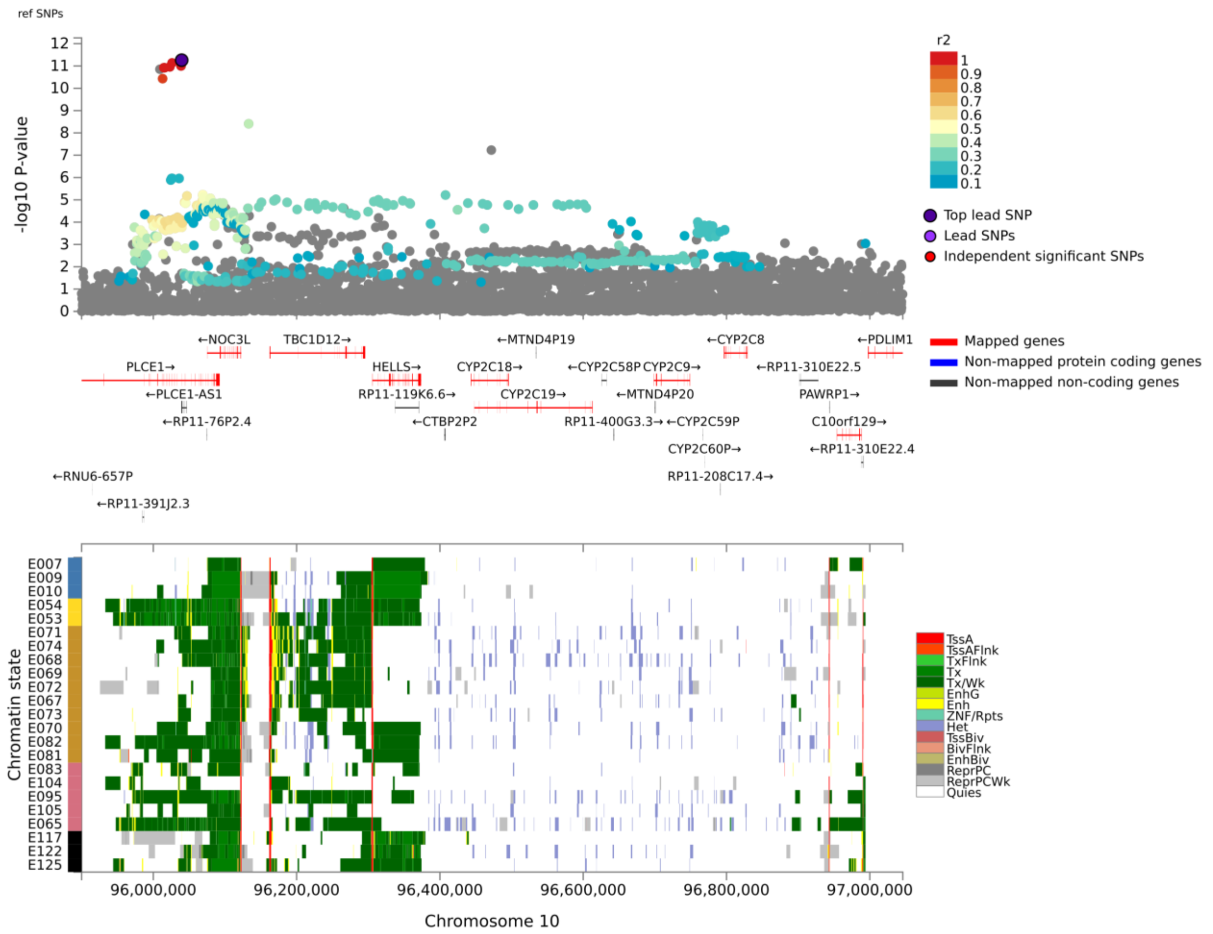

Supplementary Figure 37- Regional association plot with relevant tissue epigenomic data for global network efficiency adjusted for RSFA locus PLCE1

Global efficiency adjusted for RSFA – *C10orf91*

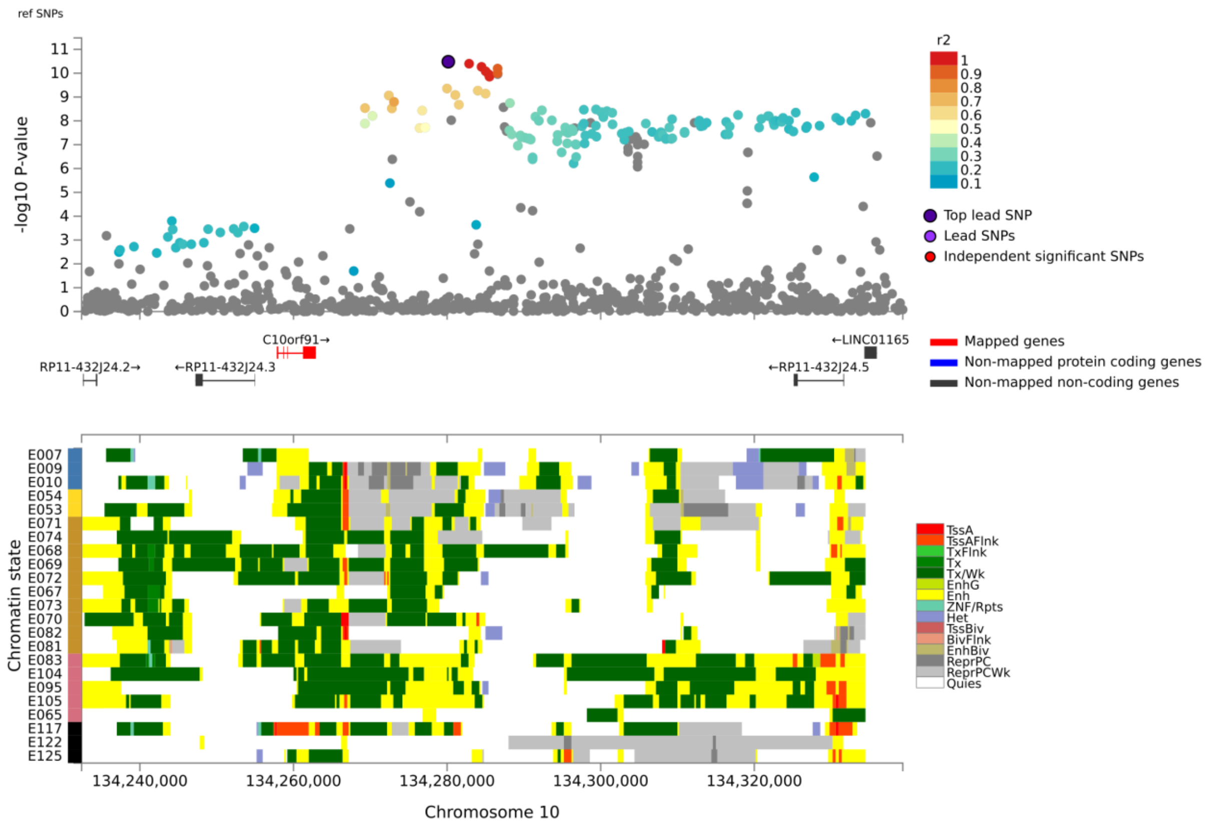

Supplementary Figure 38- Regional association plot with relevant tissue epigenomic data for global network efficiency adjusted for RSFA locus C10orf91

Global efficiency adjusted for RSFA – *EPN2*

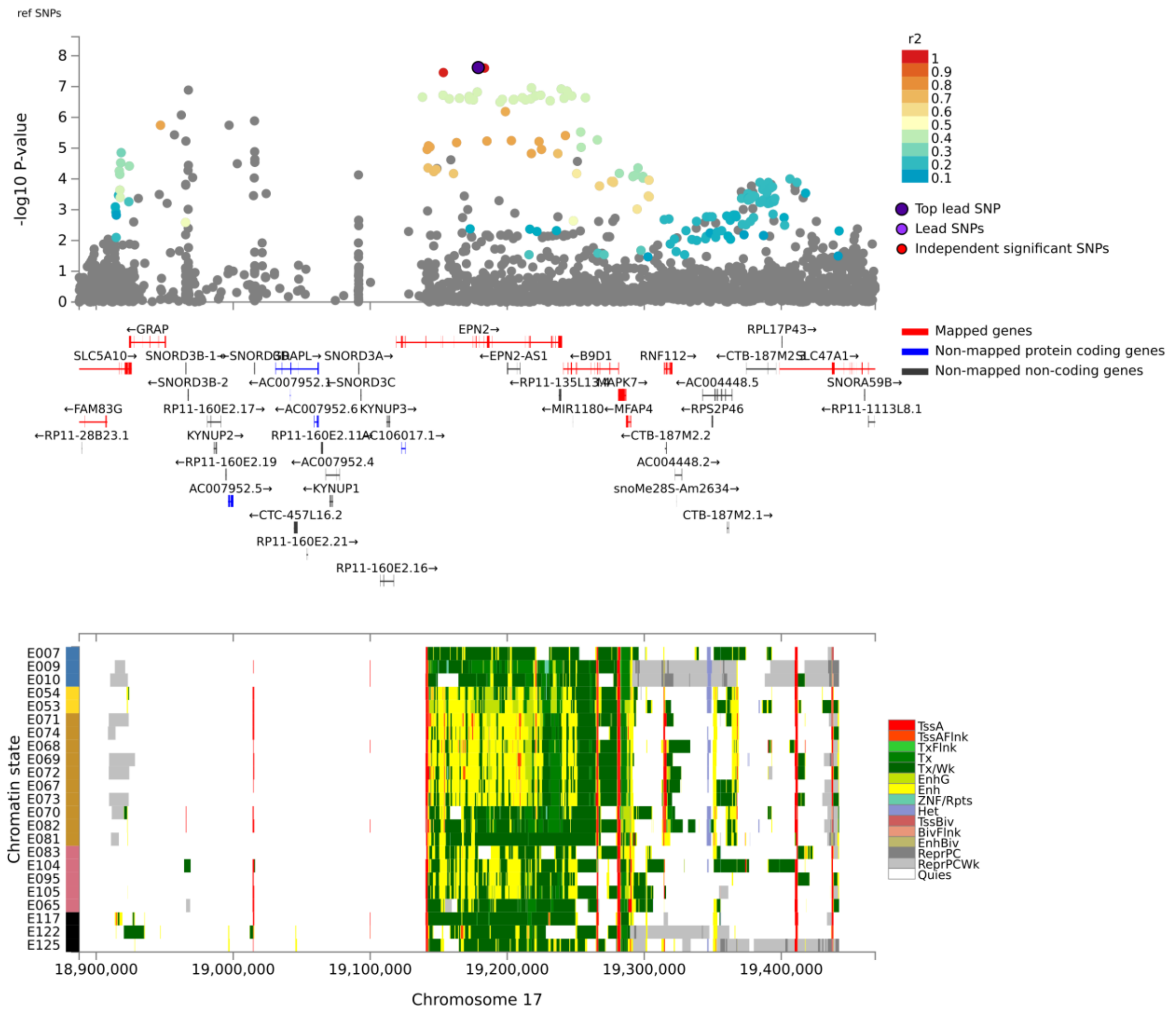

Supplementary Figure 39- Regional association plot with relevant tissue epigenomic data for global network efficiency adjusted for RSFA locus EPN2

RSFA – *HSPG2*

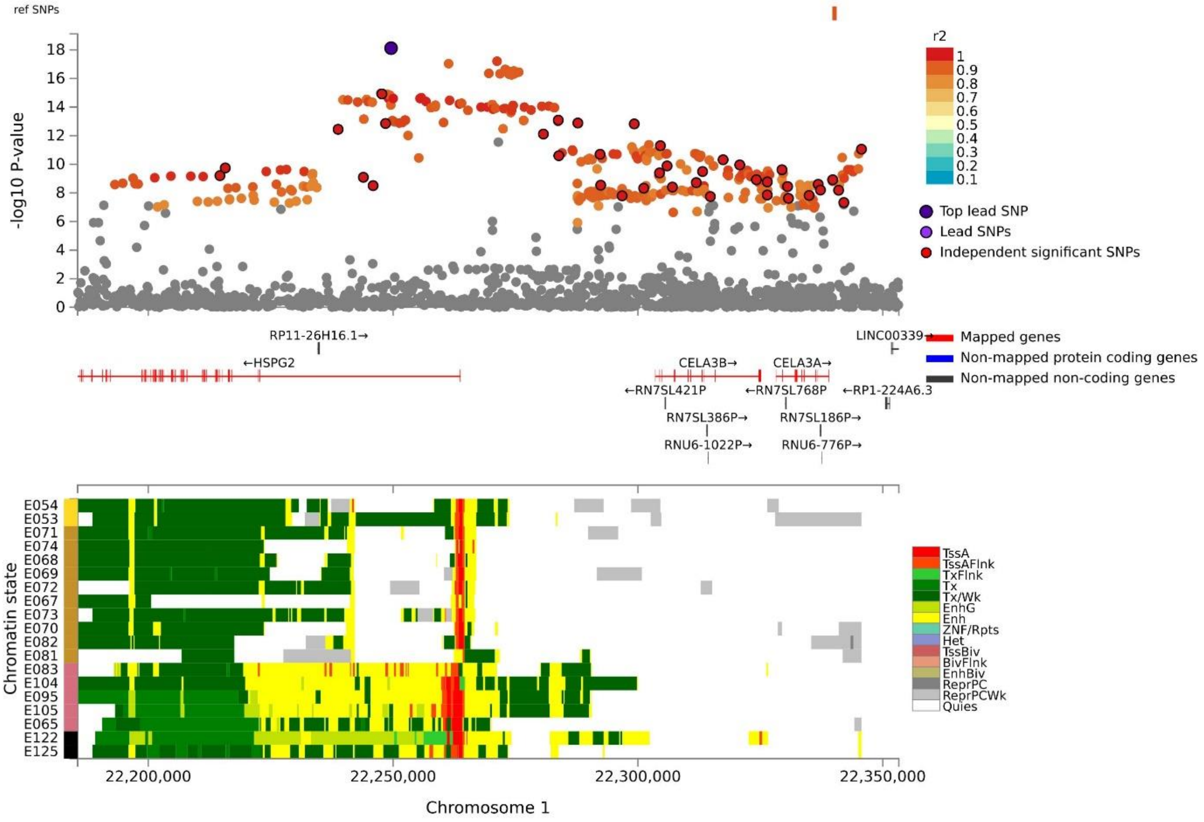

Supplementary Figure 40- Regional association plot with relevant tissue epigenomic data for RSFA locus HSPG2

RSFA – *SLC8A1*

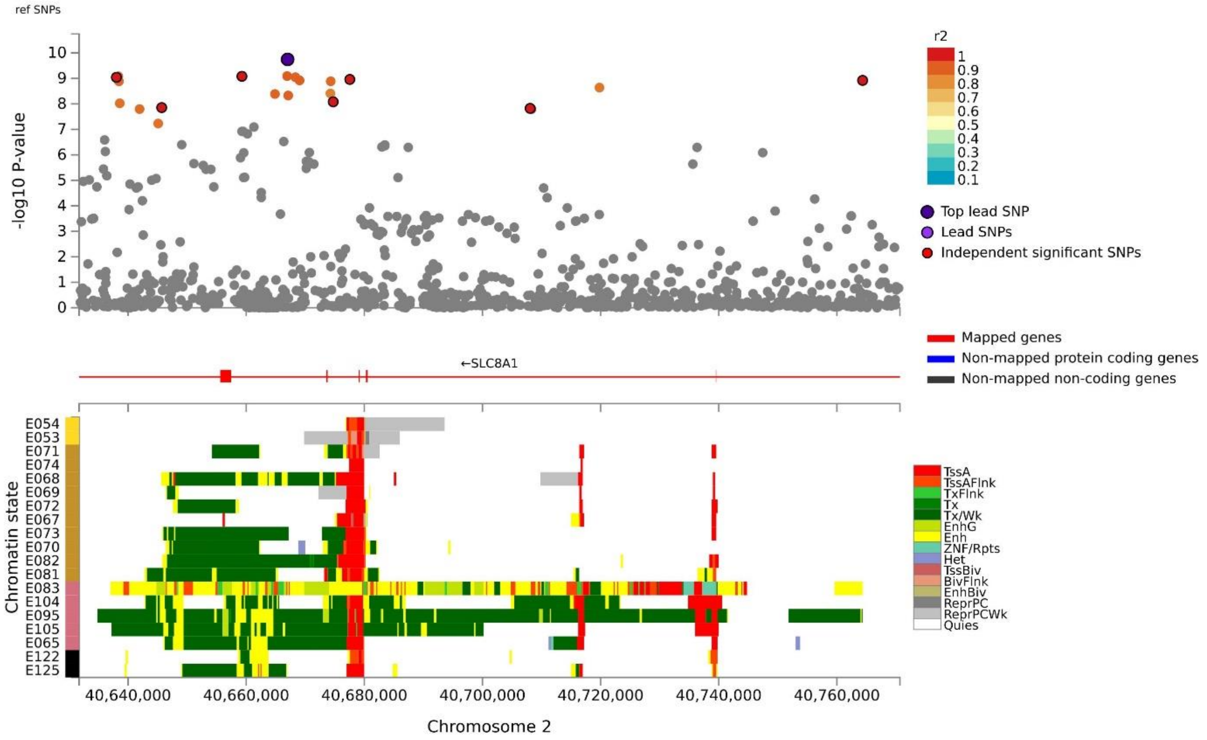

Supplementary Figure 41- Regional association plot with relevant tissue epigenomic data for RSFA locus SLC8A1

RSFA – *ITGB5*

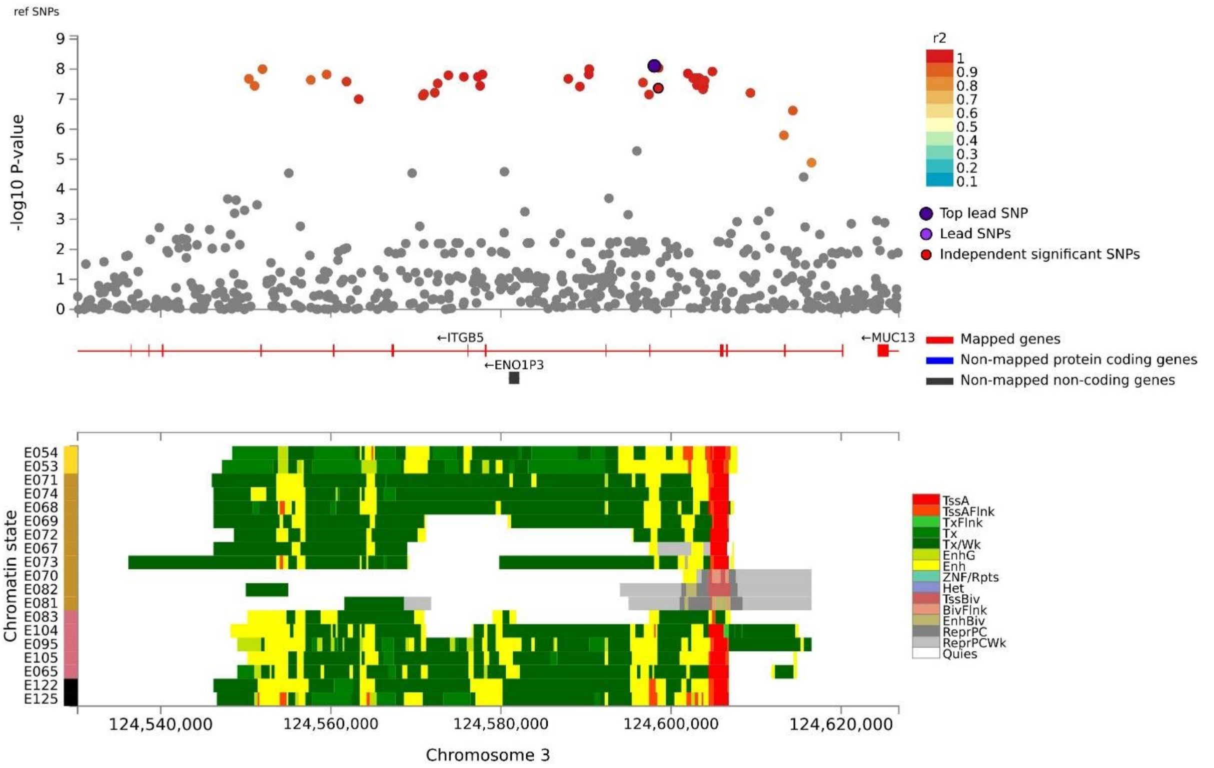

Supplementary Figure 42- Regional association plot with relevant tissue epigenomic data for RSFA locus ITGB5

RSFA – *STK32B*

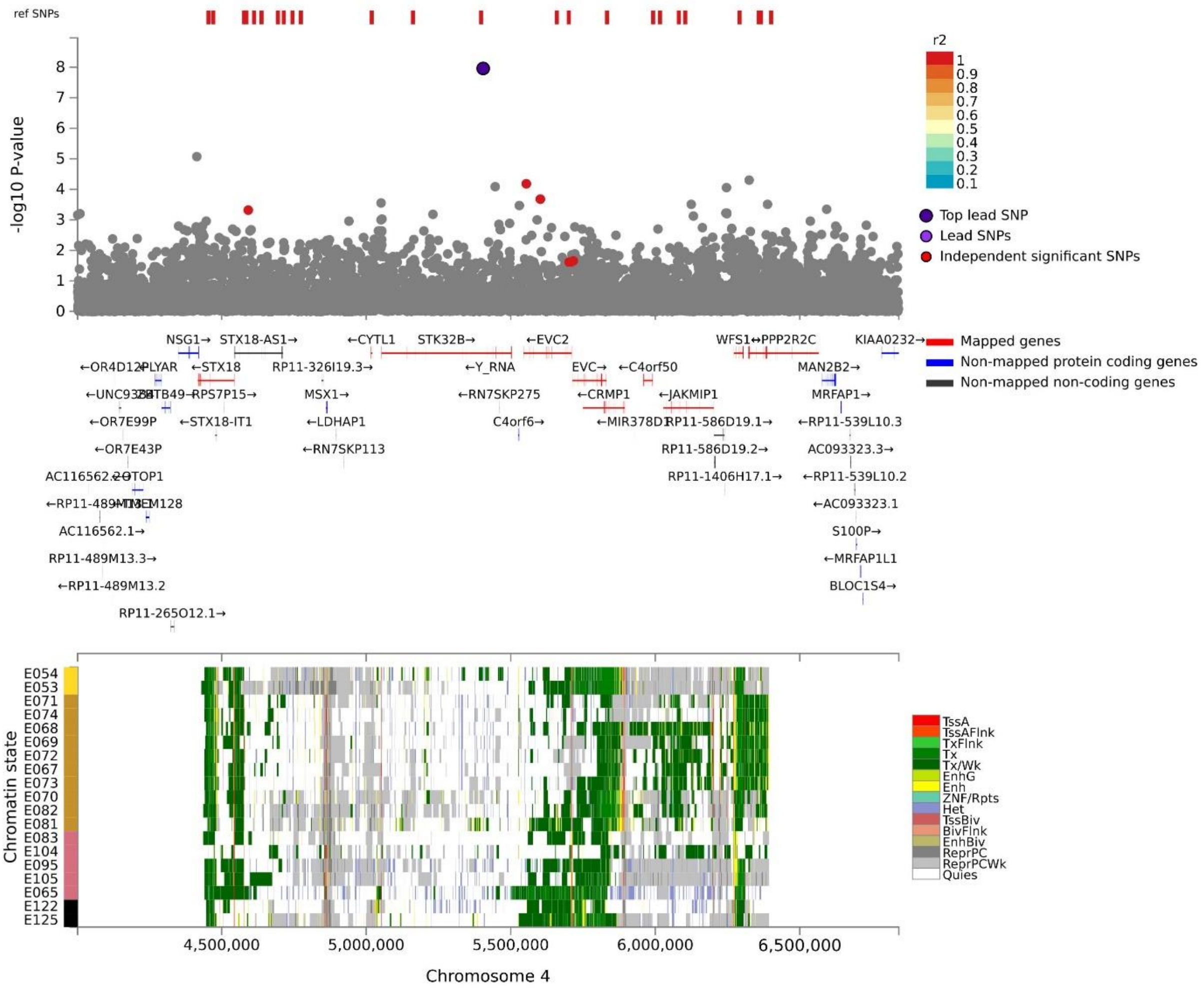

Figure 1 - Regional association plot with relevant tissue epigenomic data for RSFA locus STK32B.

RSFA – *RP11-8L2.1*

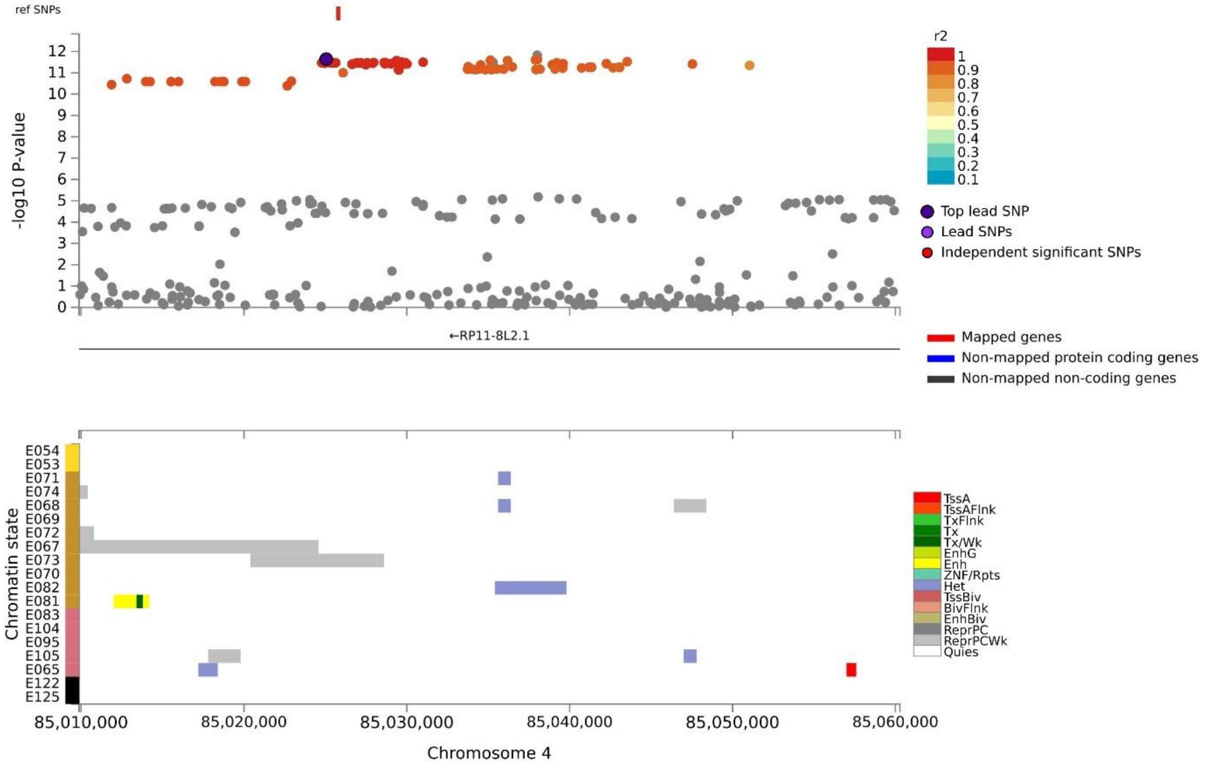

Supplementary Figure 43- Regional association plot with relevant tissue epigenomic data for RSFA locus RP11-8L2.1

RSFA – *WWC2*

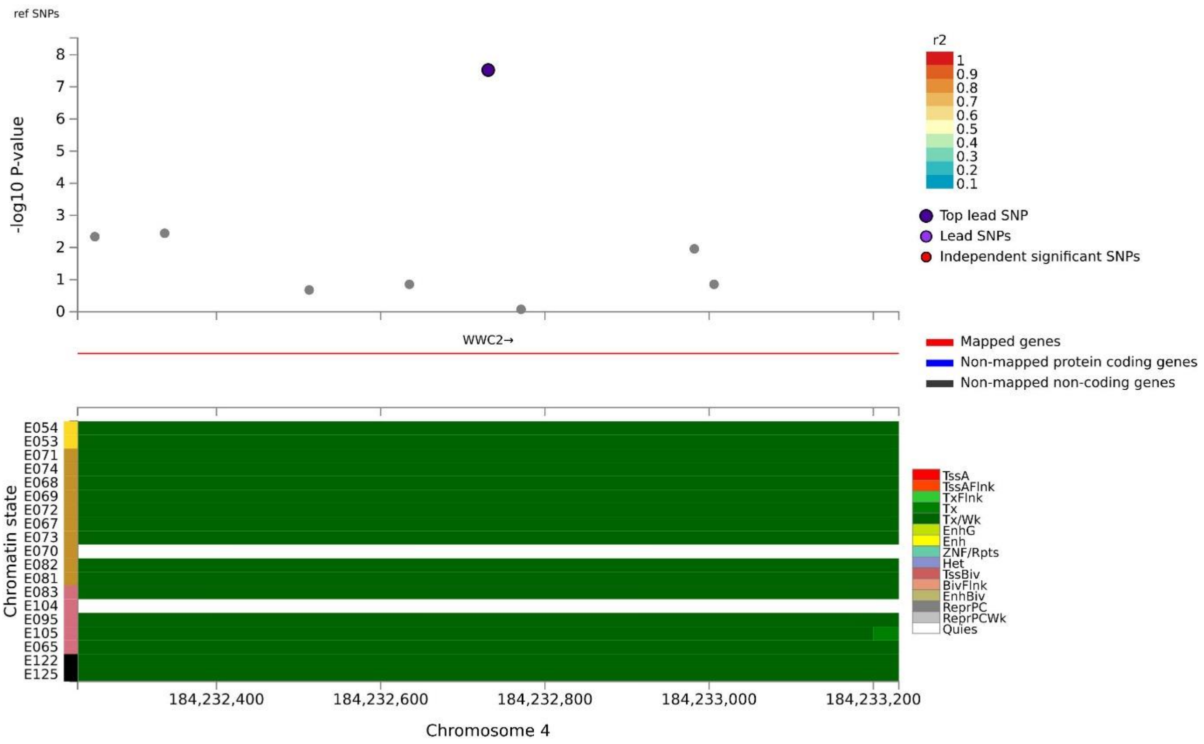

Figure 2 - Regional association plot with relevant tissue epigenomic data for RSFA locus WWC2

RSFA – *FOXQ1*

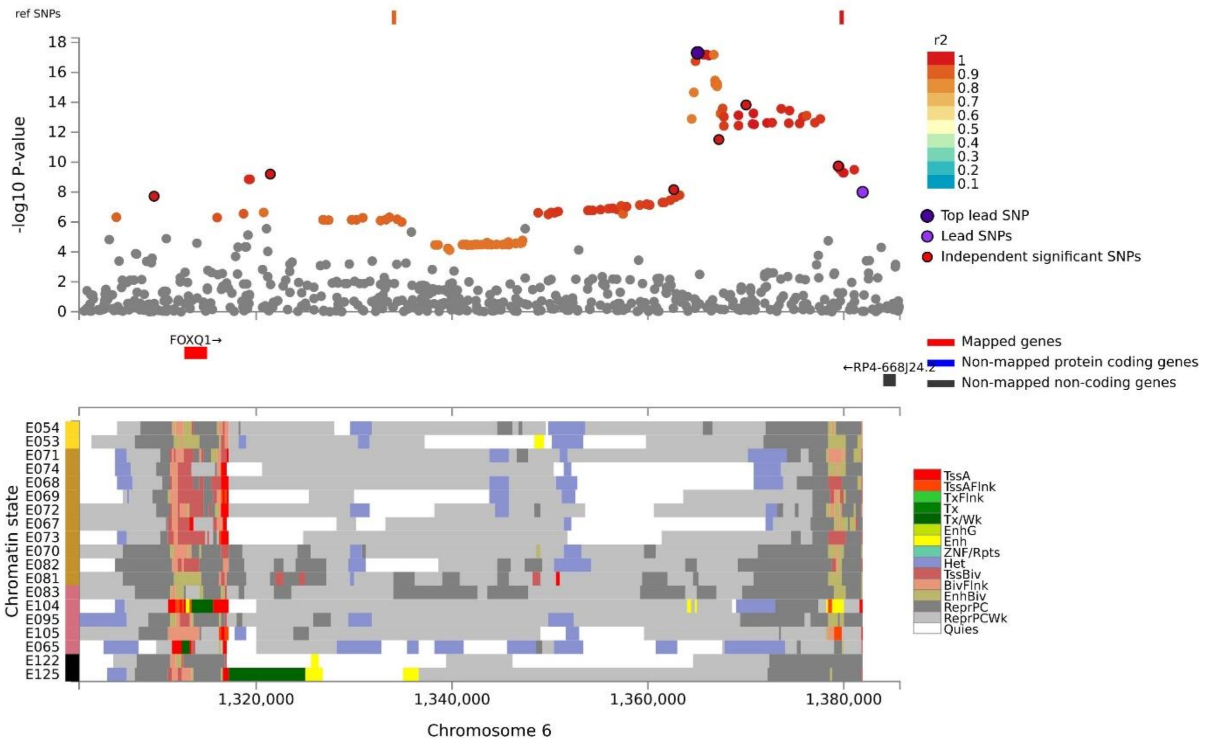

Supplementary Figure 44- Regional association plot with relevant tissue epigenomic data for RSFA locus FOXQ1

RSFA – *SENP6*

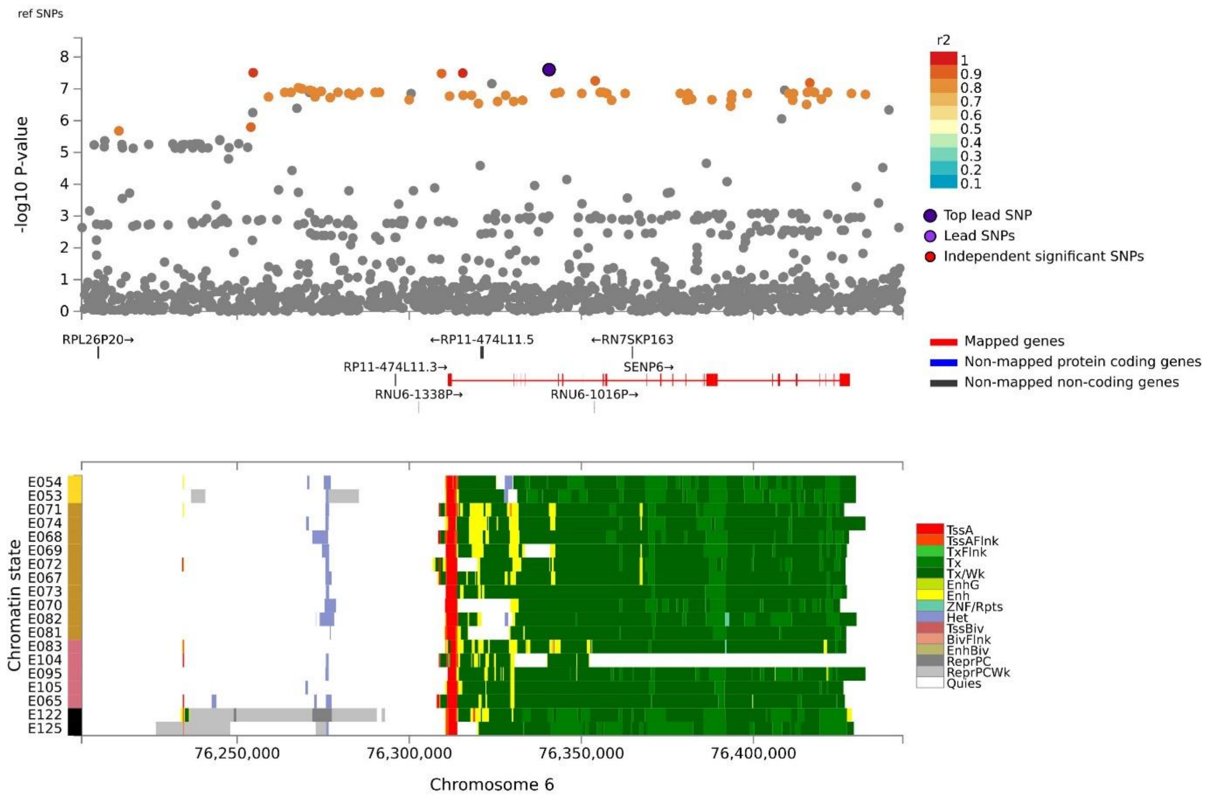

Supplementary Figure 45- Regional association plot with relevant tissue epigenomic data for RSFA locus SENP6

RSFA – *UFL1*

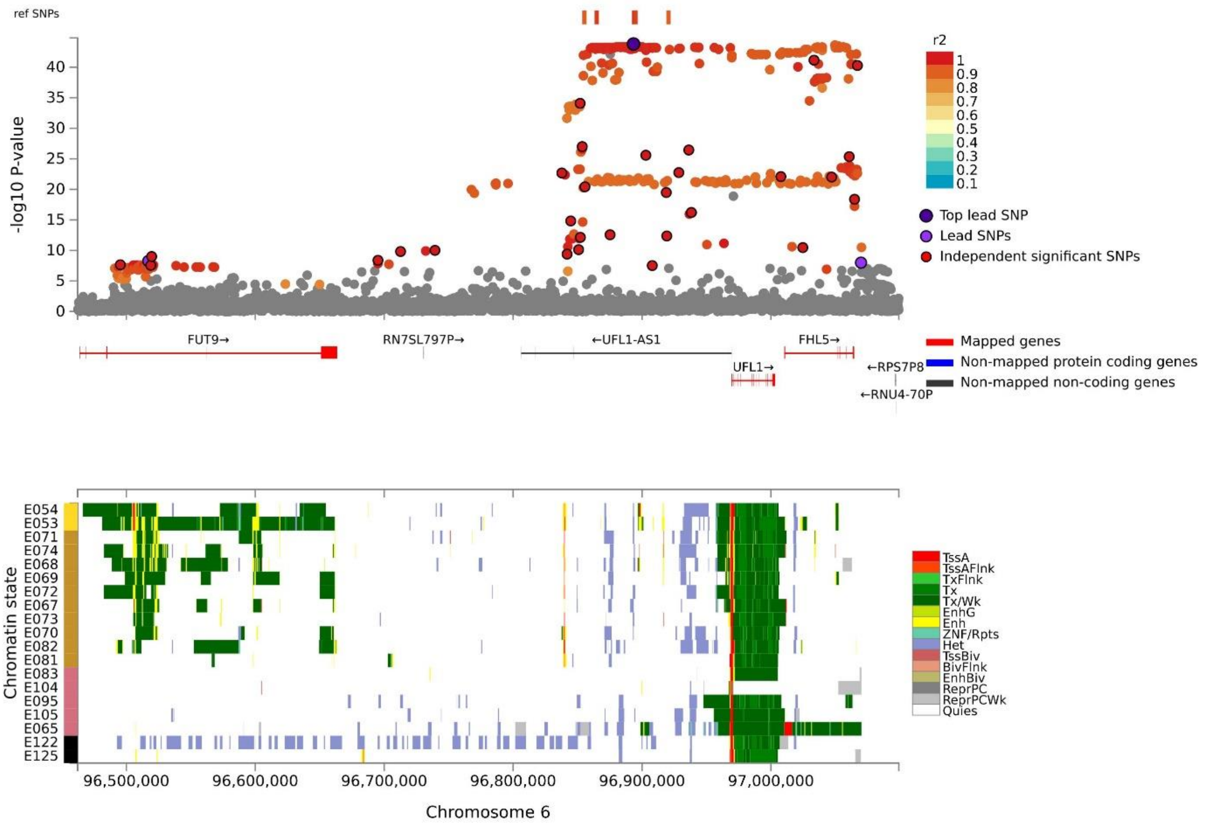

Supplementary Figure 46- Regional association plot with relevant tissue epigenomic data for RSFA locus UFL1

RSFA – *DGKB*

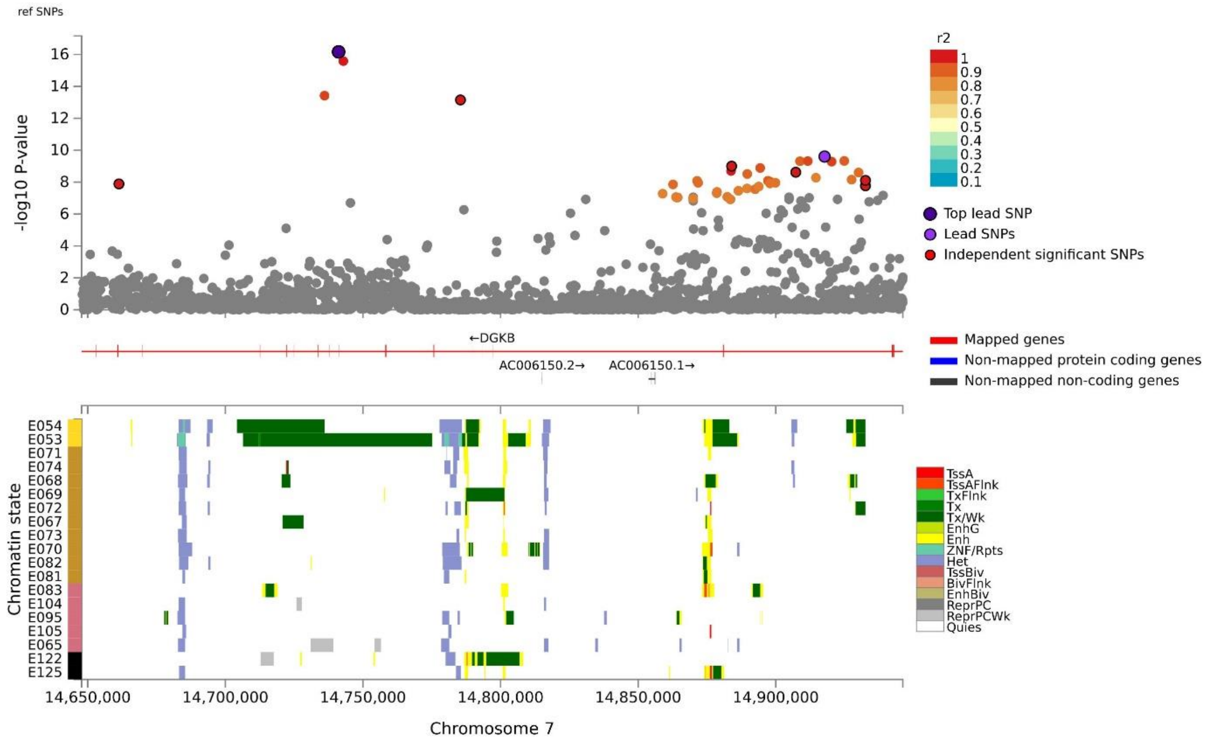

Supplementary Figure 47- Regional association plot with relevant tissue epigenomic data for RSFA locus DGKB

RSFA – *EGFR*

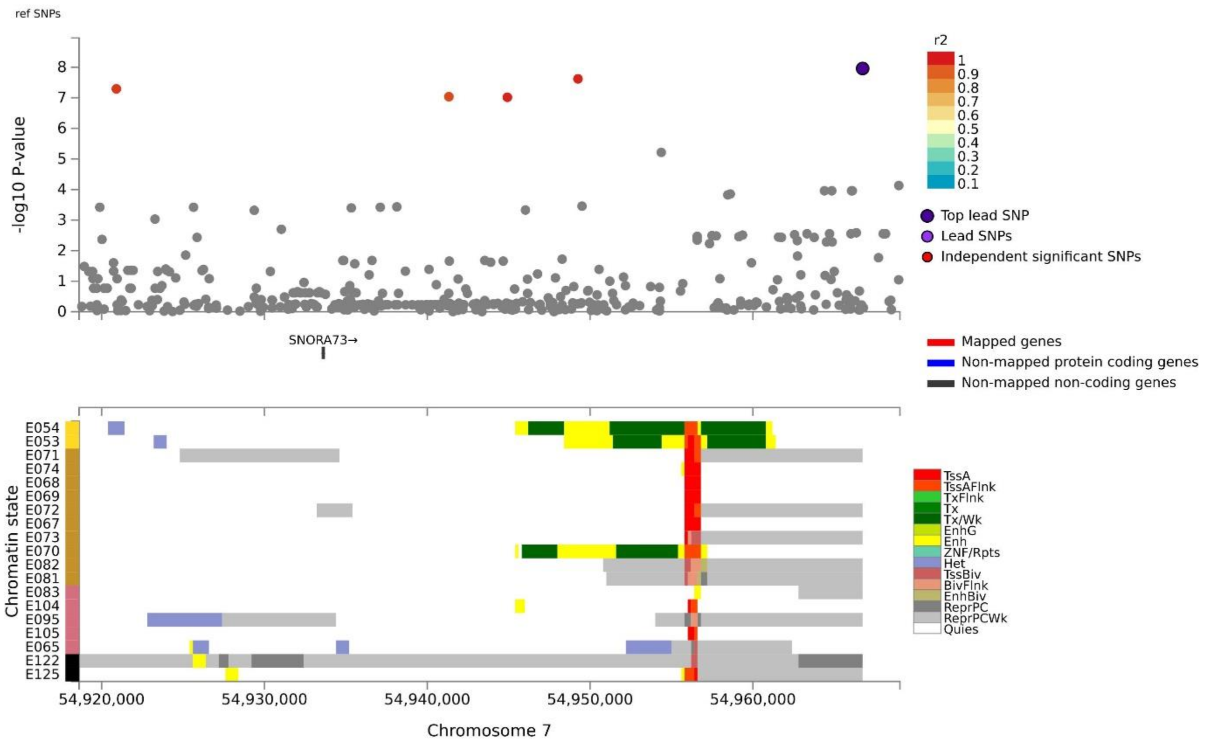

Supplementary Figure 48- Regional association plot with relevant tissue epigenomic data for RSFA locus EGFR

RSFA – *PIP5K1B*

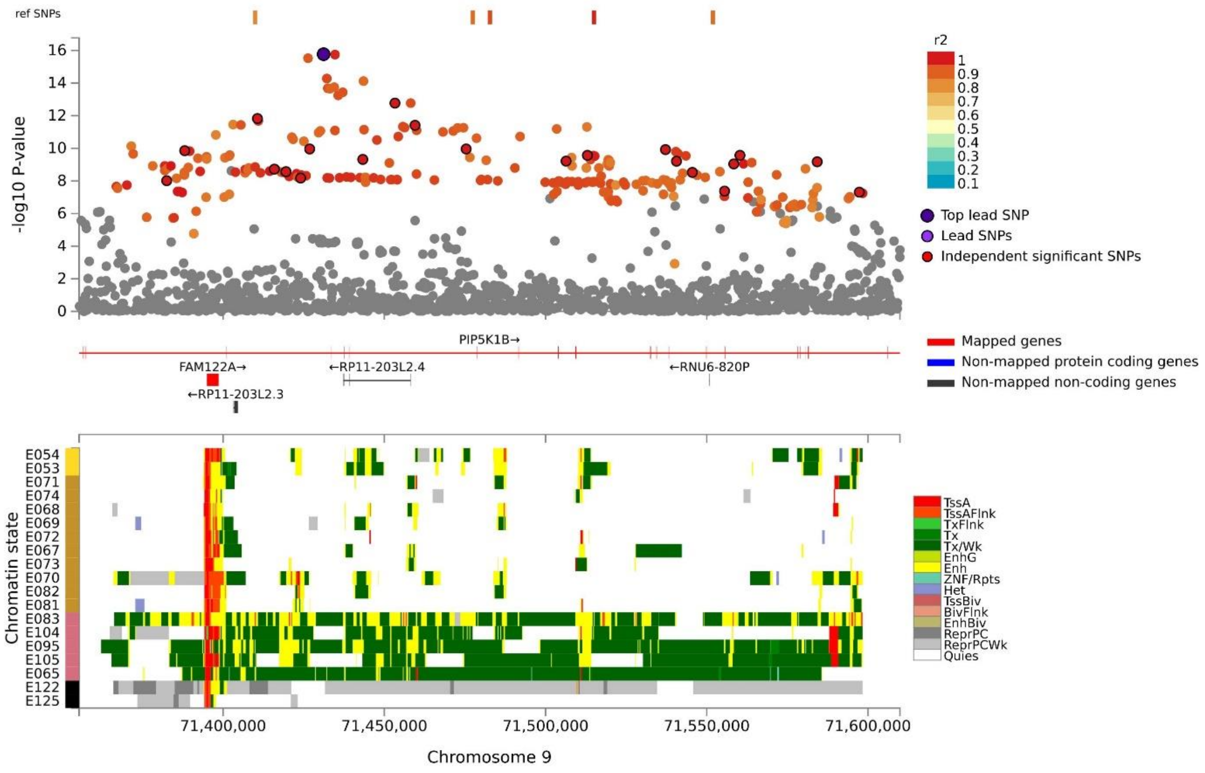

Supplementary Figure 49- Regional association plot with relevant tissue epigenomic data for RSFA locus PIP5K1B

RSFA – *PLCE1*

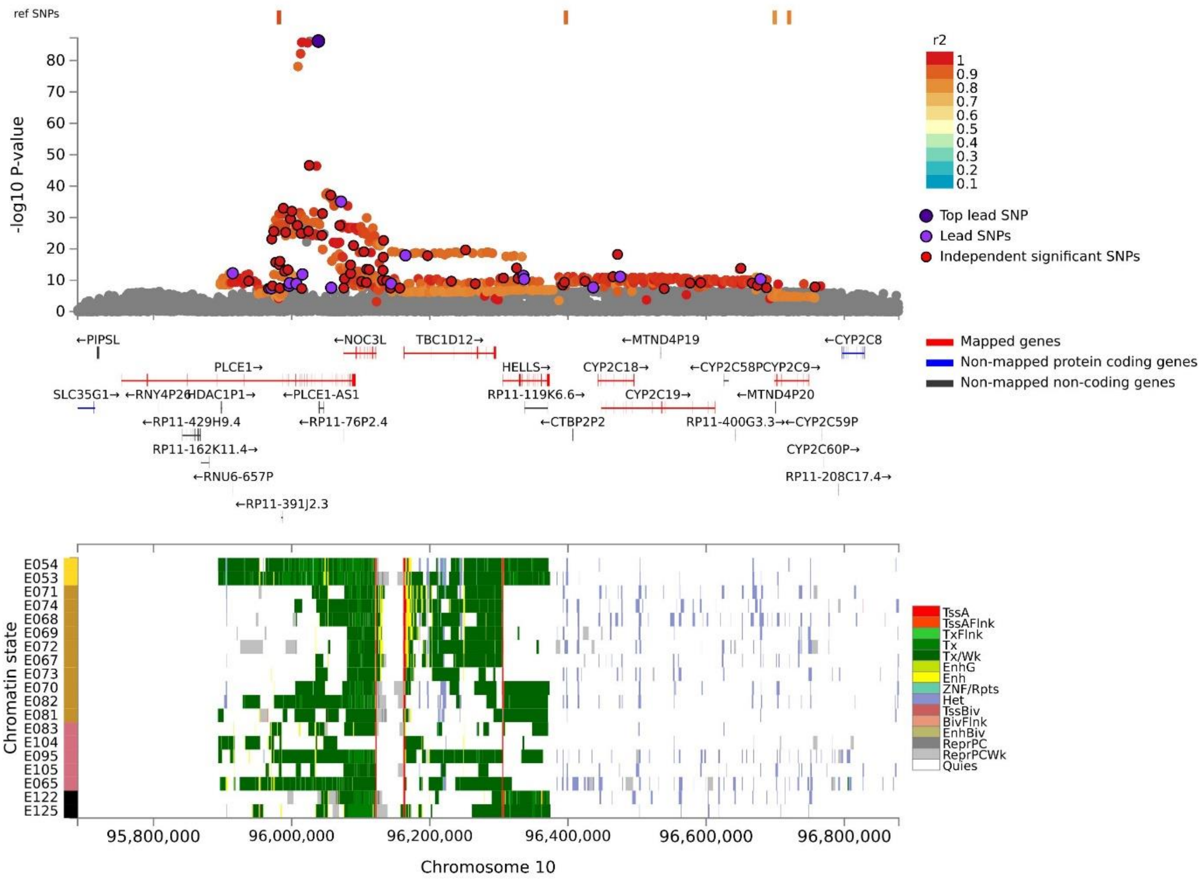

Supplementary Figure 50- Regional association plot with relevant tissue epigenomic data for RSFA locus PLCE1

RSFA – *C10orf91-INPP5A*

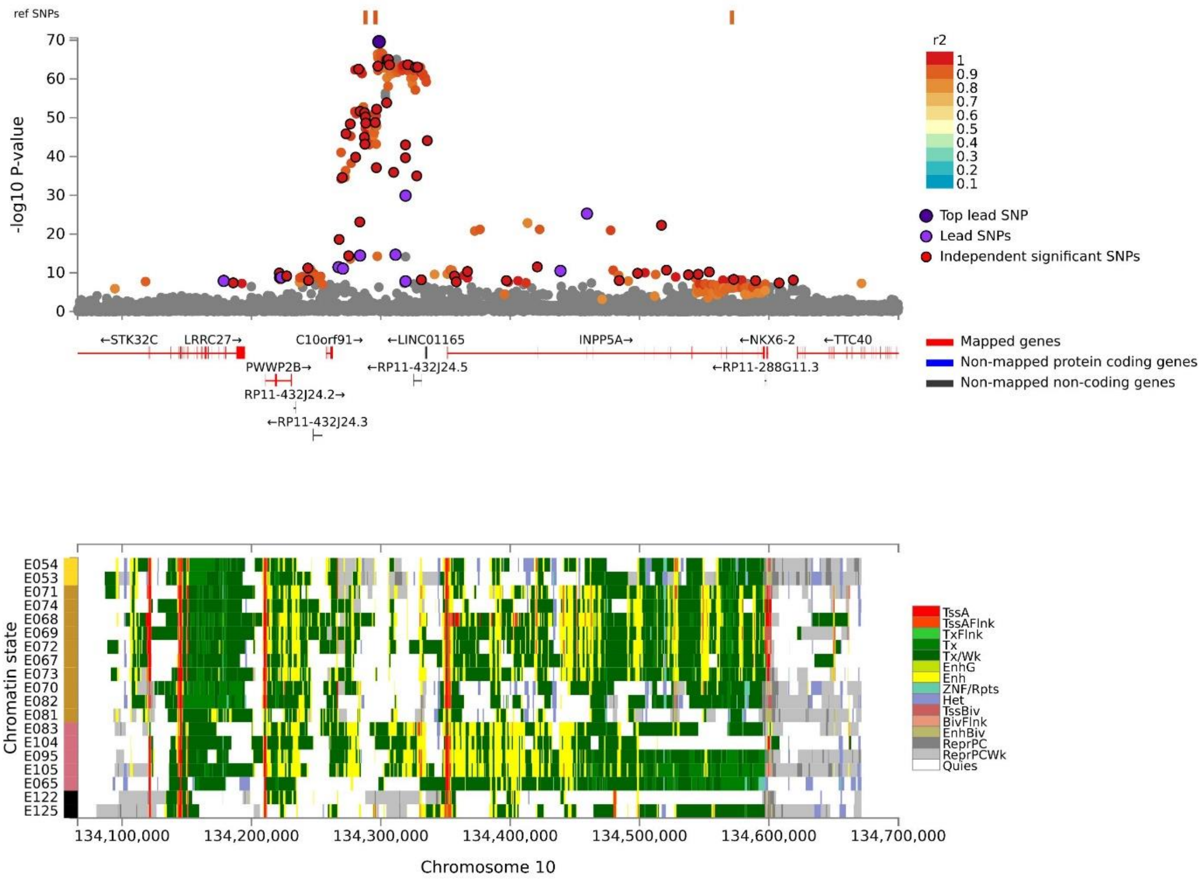

Supplementary Figure 51- Regional association plot with relevant tissue epigenomic data for RSFA locus C10orf91-INPP5A

RSFA – *IFITM2*

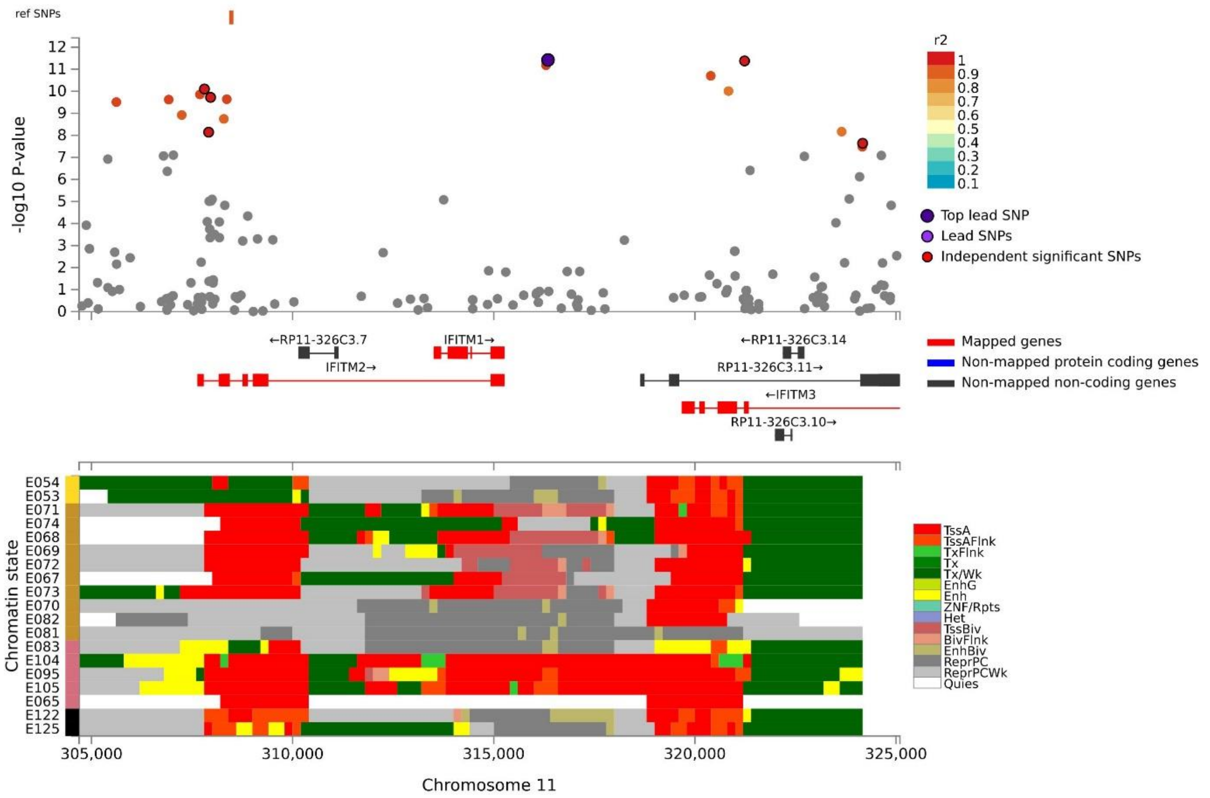

Supplementary Figure 52- Regional association plot with relevant tissue epigenomic data for RSFA locus IFITM2

RSFA – *MRVI1*

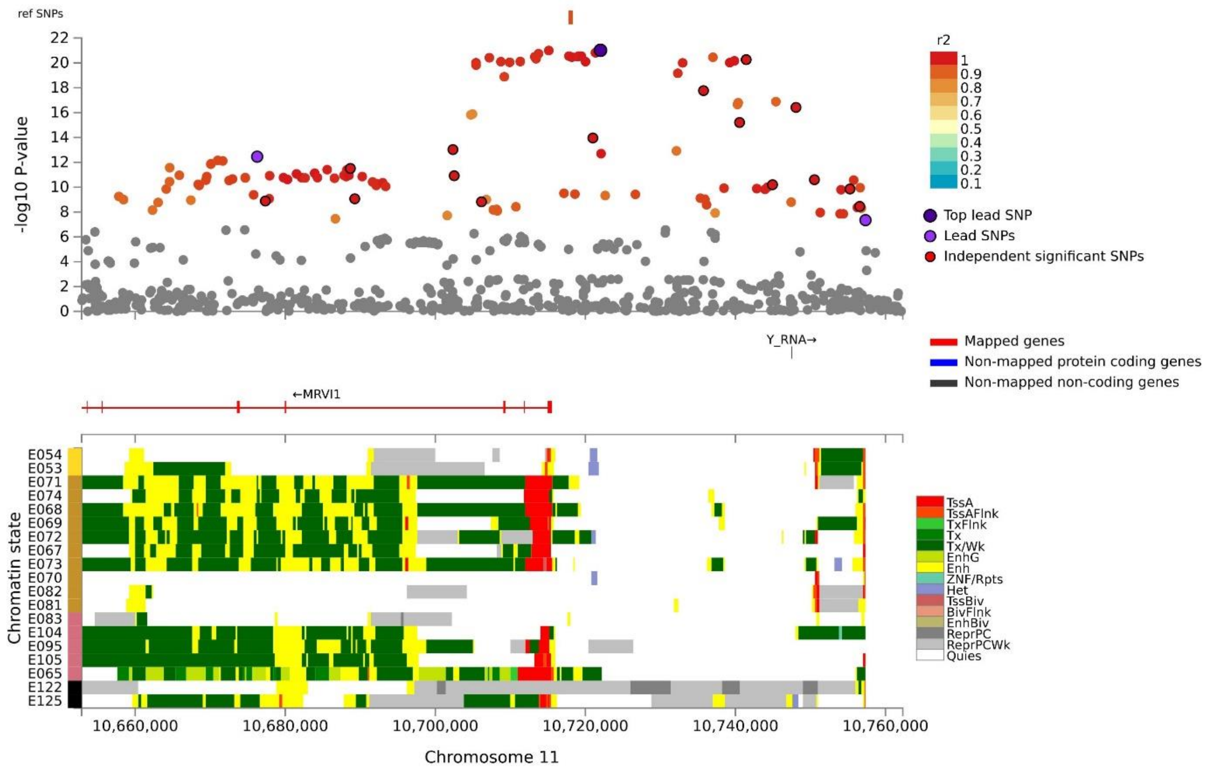

Supplementary Figure 53- Regional association plot with relevant tissue epigenomic data for RSFA locus MRVI1

RSFA – *ANO3*

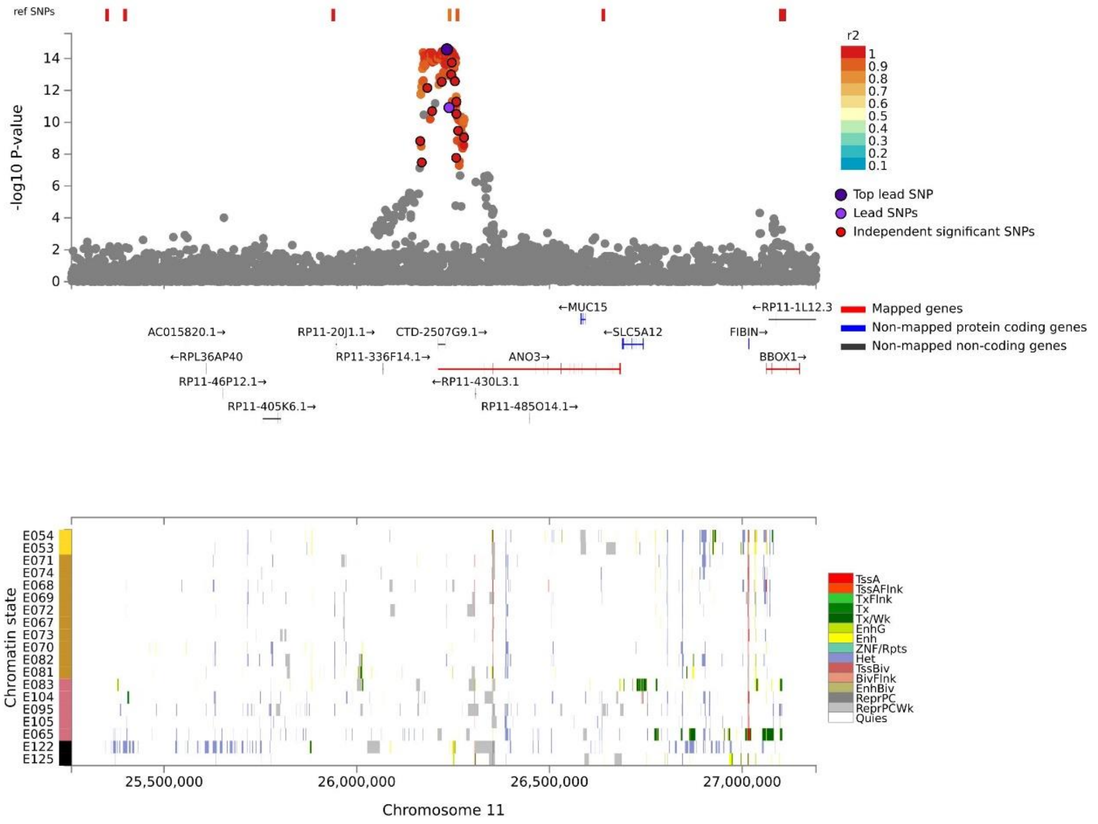

Supplementary Figure 54- Regional association plot with relevant tissue epigenomic data for RSFA locus ANO3

RSFA – *ANO1*

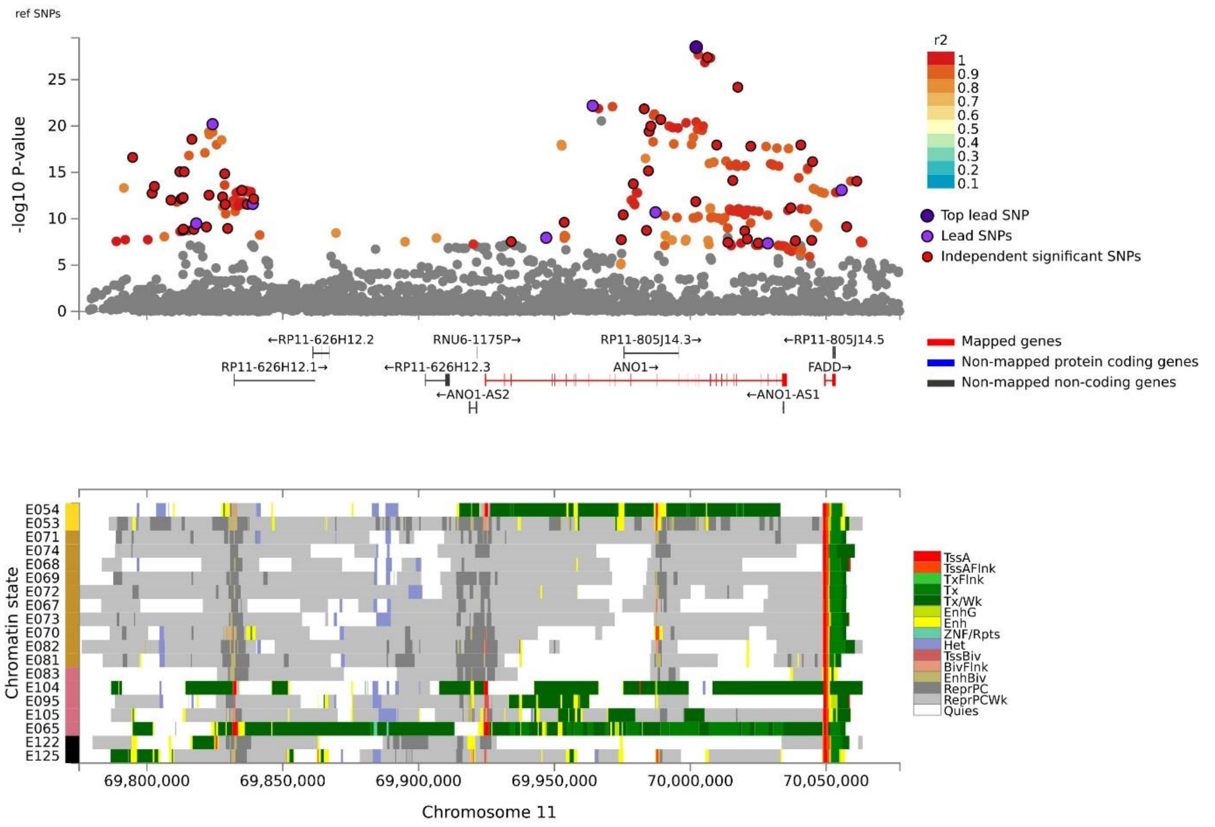

Supplementary Figure 55- Regional association plot with relevant tissue epigenomic data for RSFA locus ANO1

RSFA – *TRPC6*

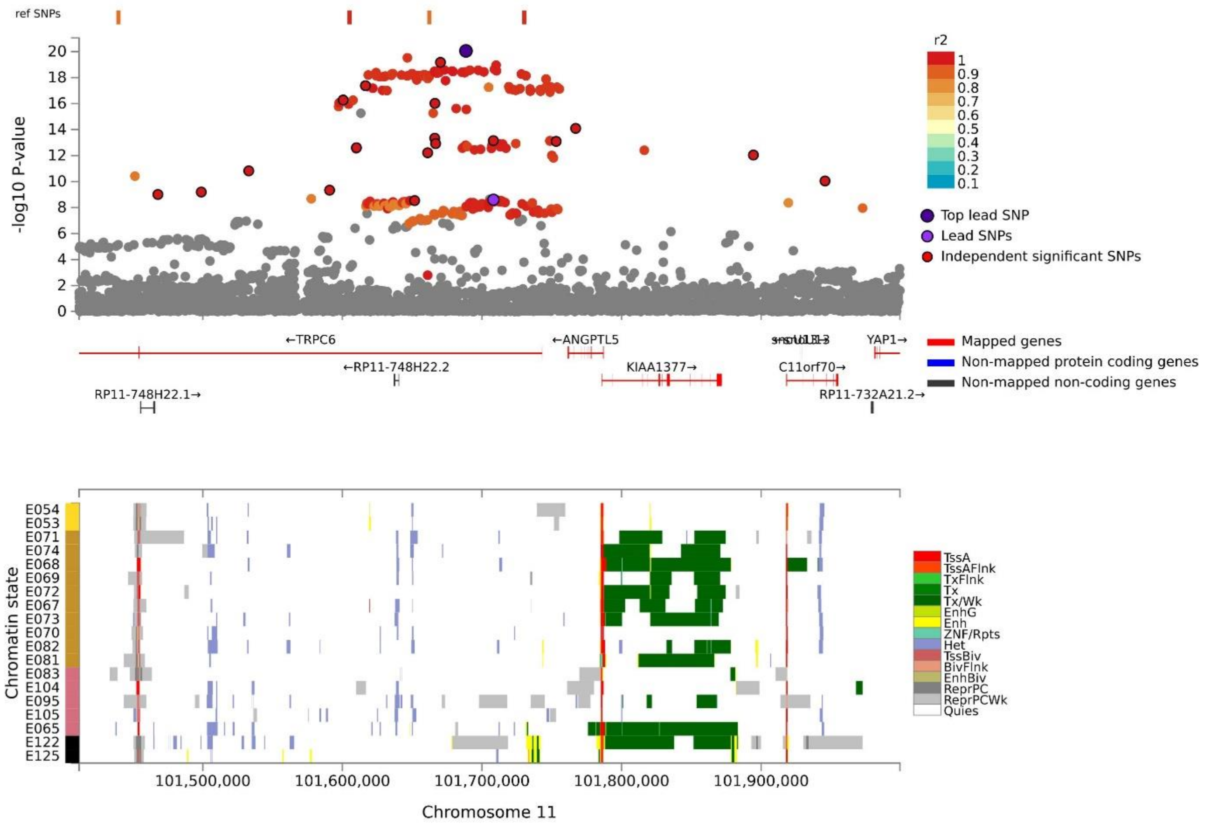

Supplementary Figure 56- Regional association plot with relevant tissue epigenomic data for RSFA locus TRPC6

RSFA – *PAWR*

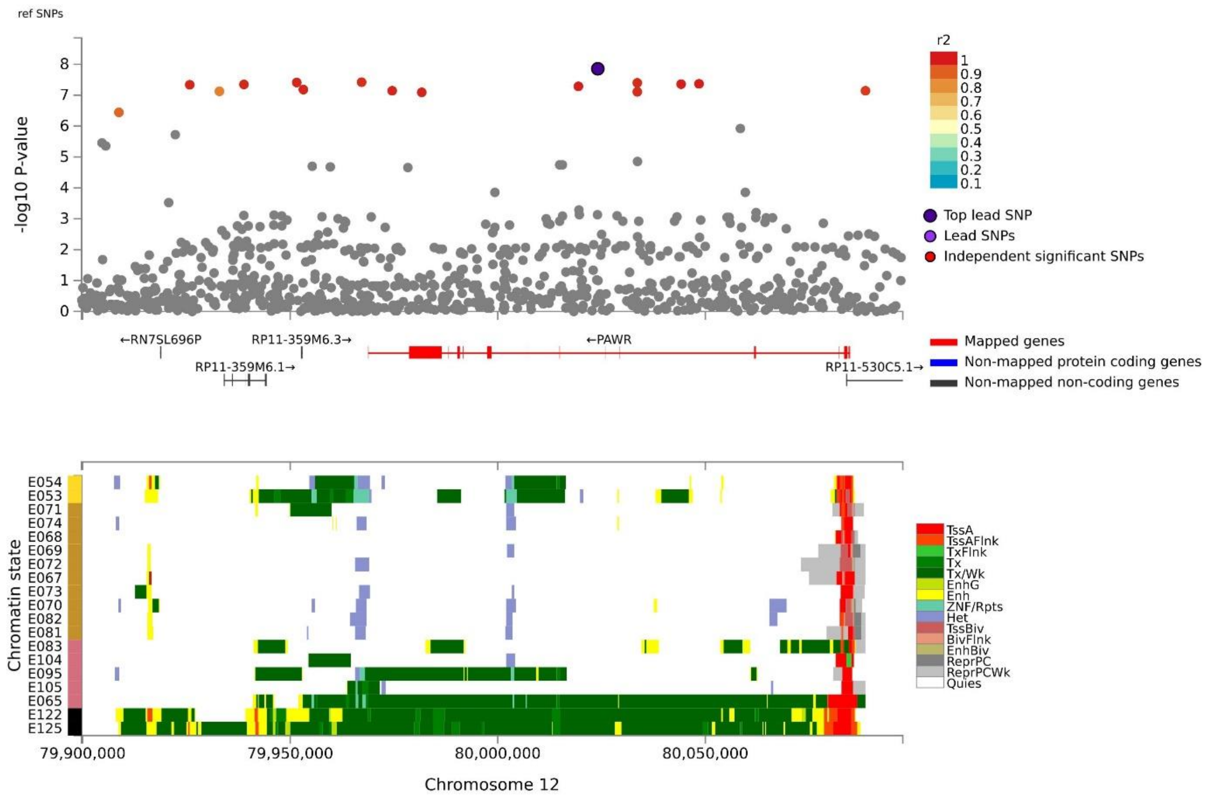

Supplementary Figure 57- Regional association plot with relevant tissue epigenomic data for RSFA locus PAWR

RSFA – *ATP2B1*

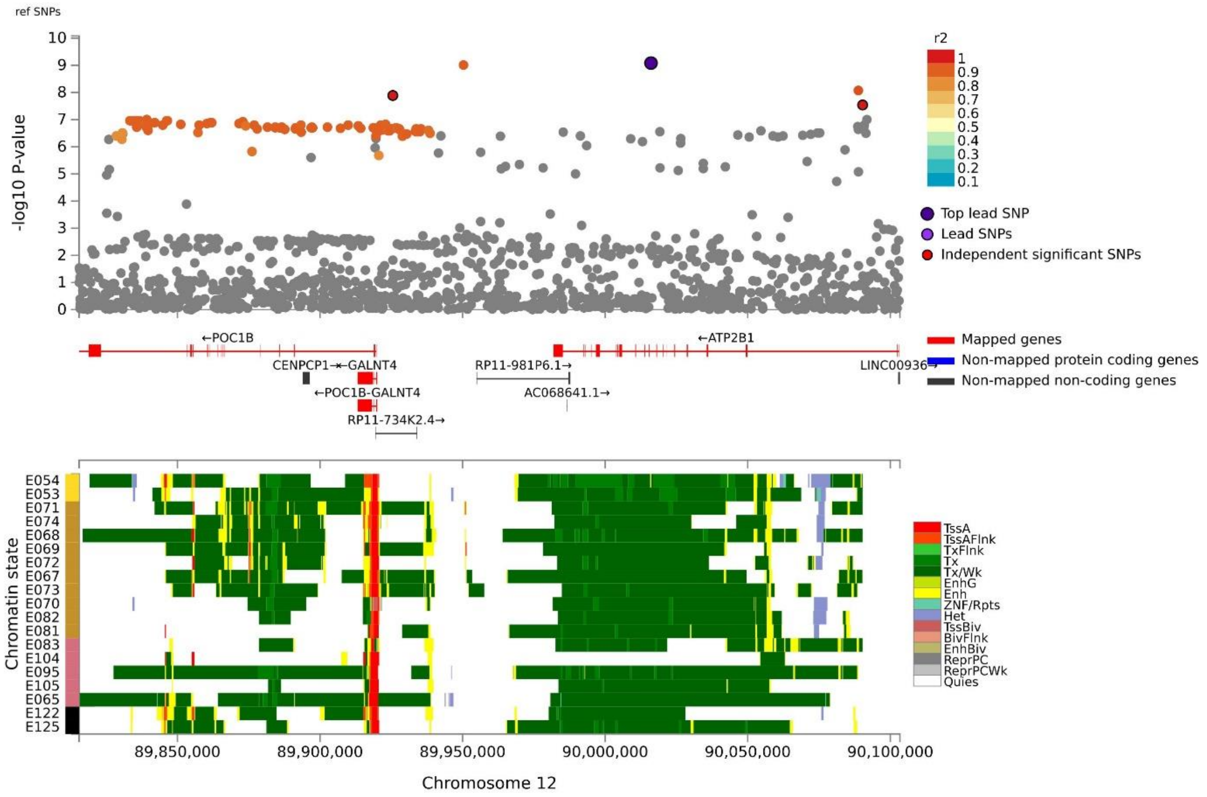

Supplementary Figure 58- Regional association plot with relevant tissue epigenomic data for RSFA locus ATP2B1

RSFA – *HIC1*

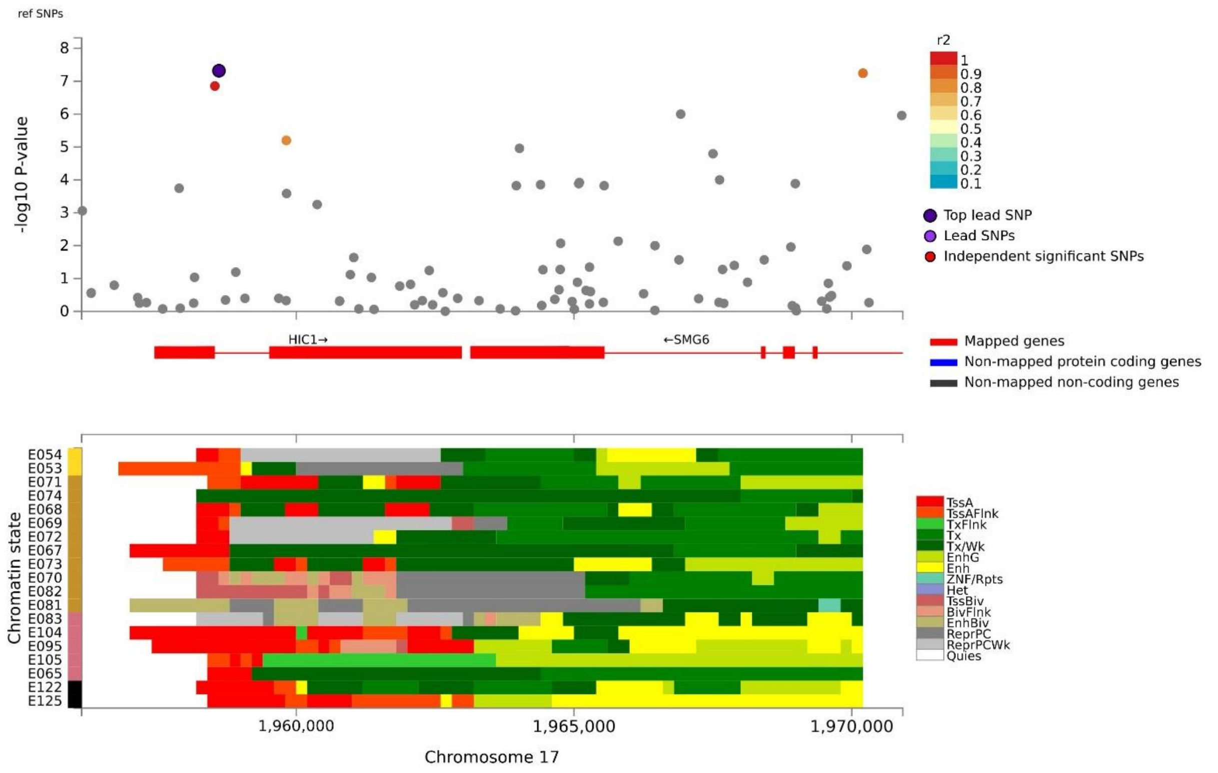

Supplementary Figure 59- Regional association plot with relevant tissue epigenomic data for RSFA locus HIC1

RSFA – *EPN2*

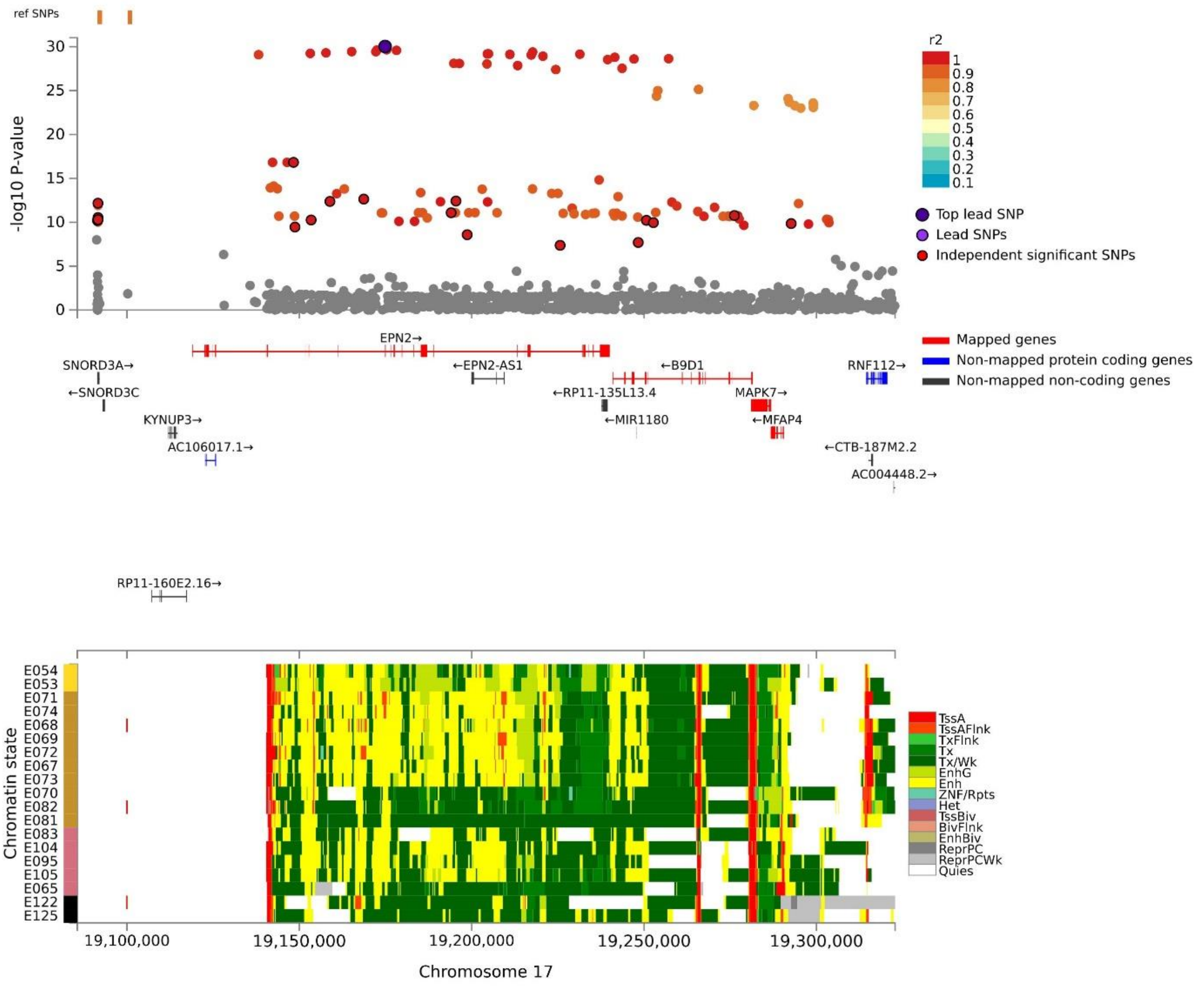

Supplementary Figure 60- Regional association plot with relevant tissue epigenomic data for RSFA locus EPN2

RSFA – *APOE*

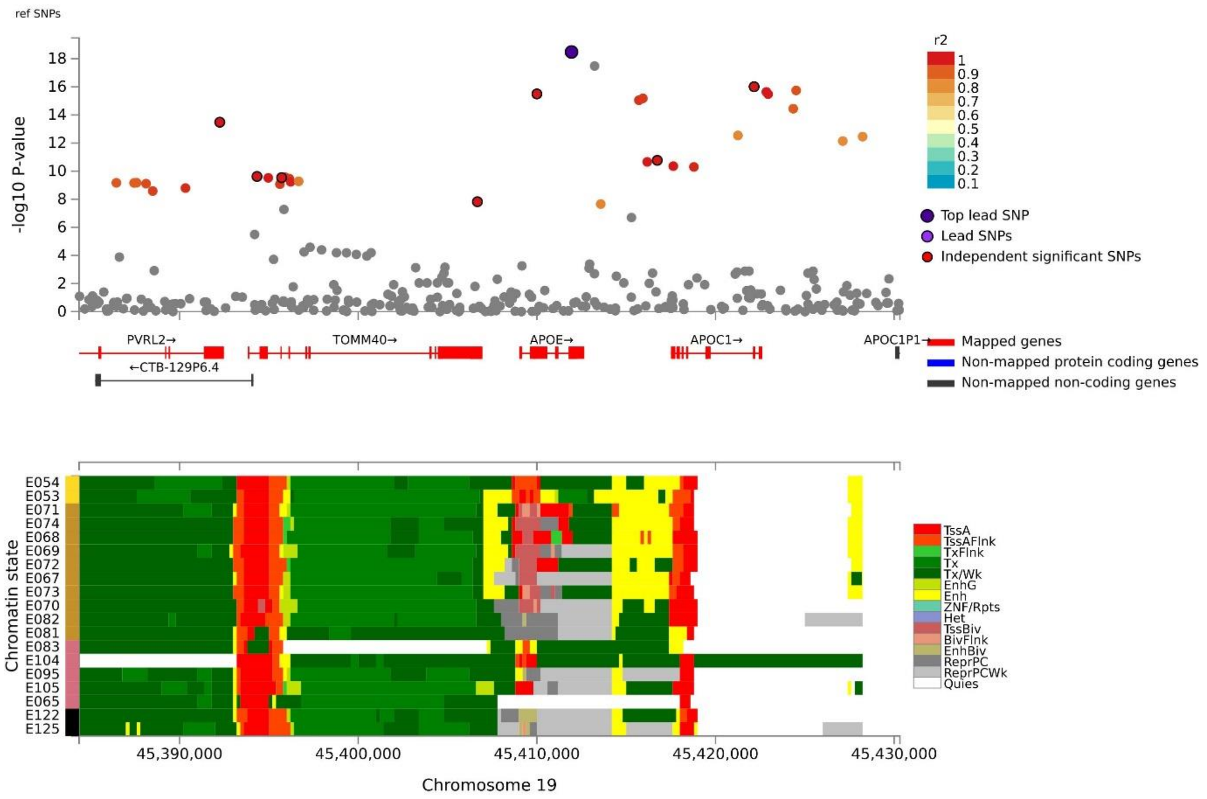

Supplementary Figure 61- Regional association plot with relevant tissue epigenomic data for RSFA locus APOE

Associations without (upper) and with (lower) adjustment for RSFA

Global efficiency

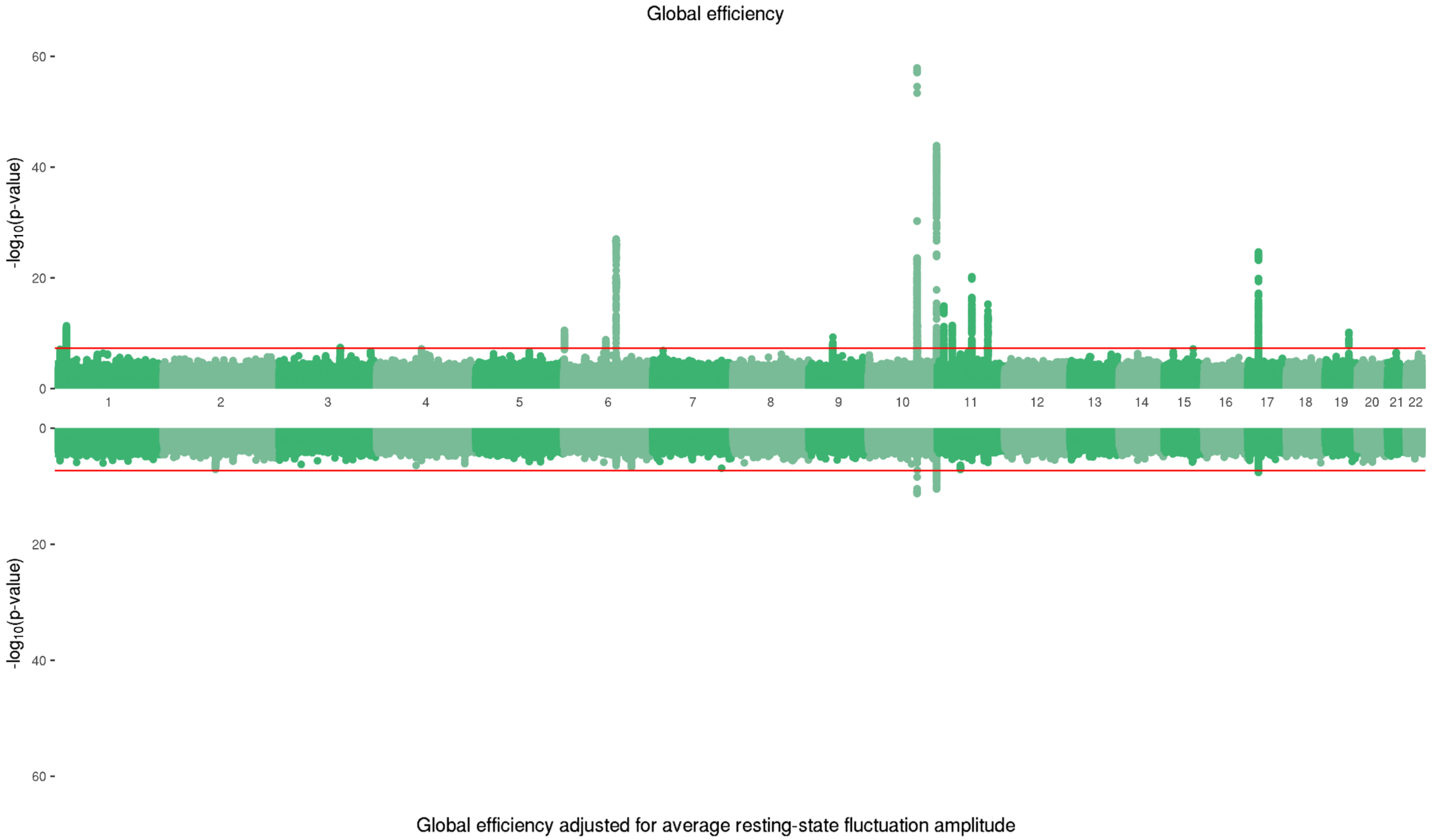

Supplementary Figure 62- Miami plot for GWAS of global network efficiency with (bottom) and without (top) adjustment for RSFA

Local efficiency

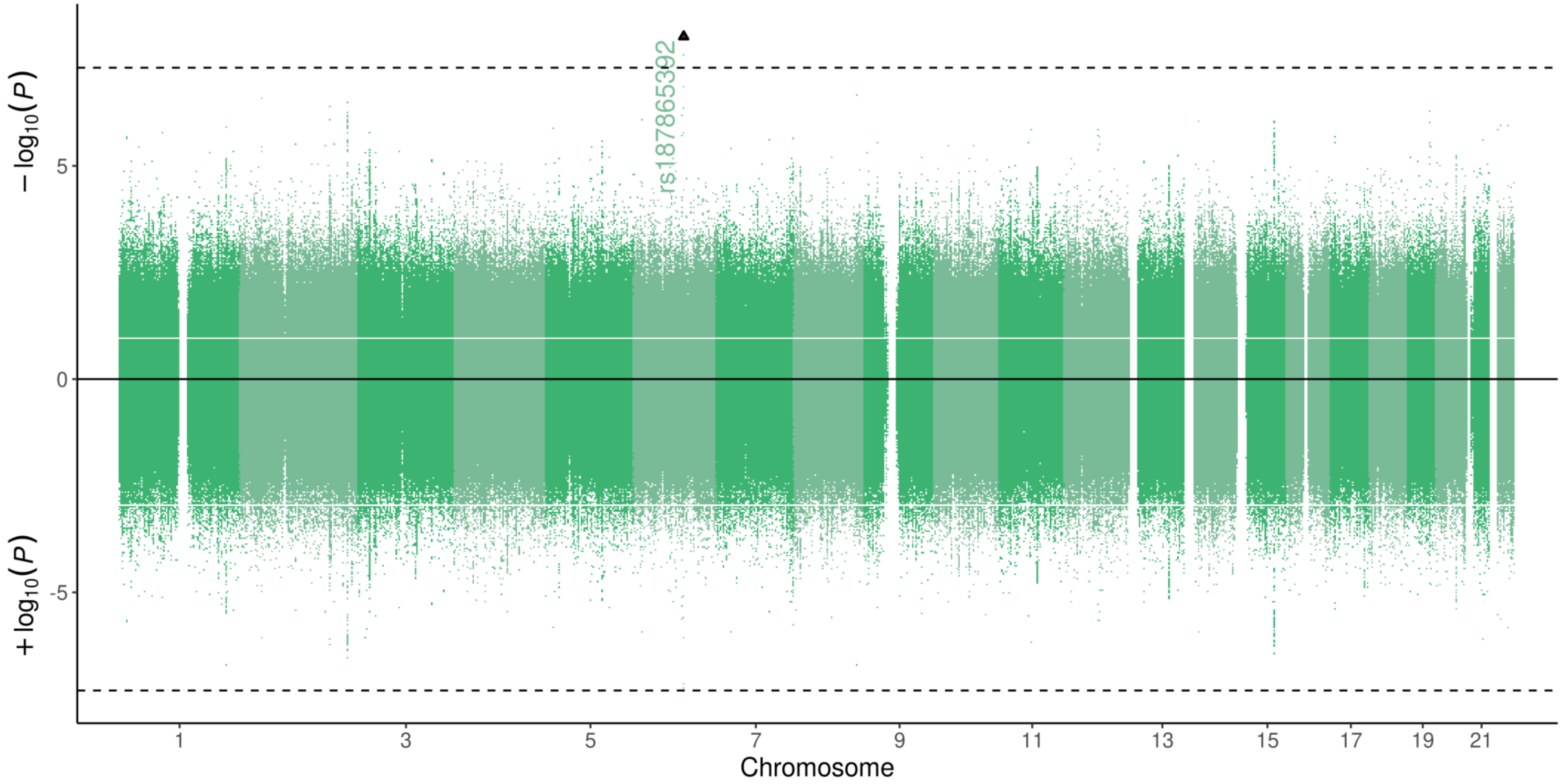

Supplementary Figure 63- Miami plot for GWAS of local network efficiency with (bottom) and without (top) adjustment for RSFA

Default-mode network

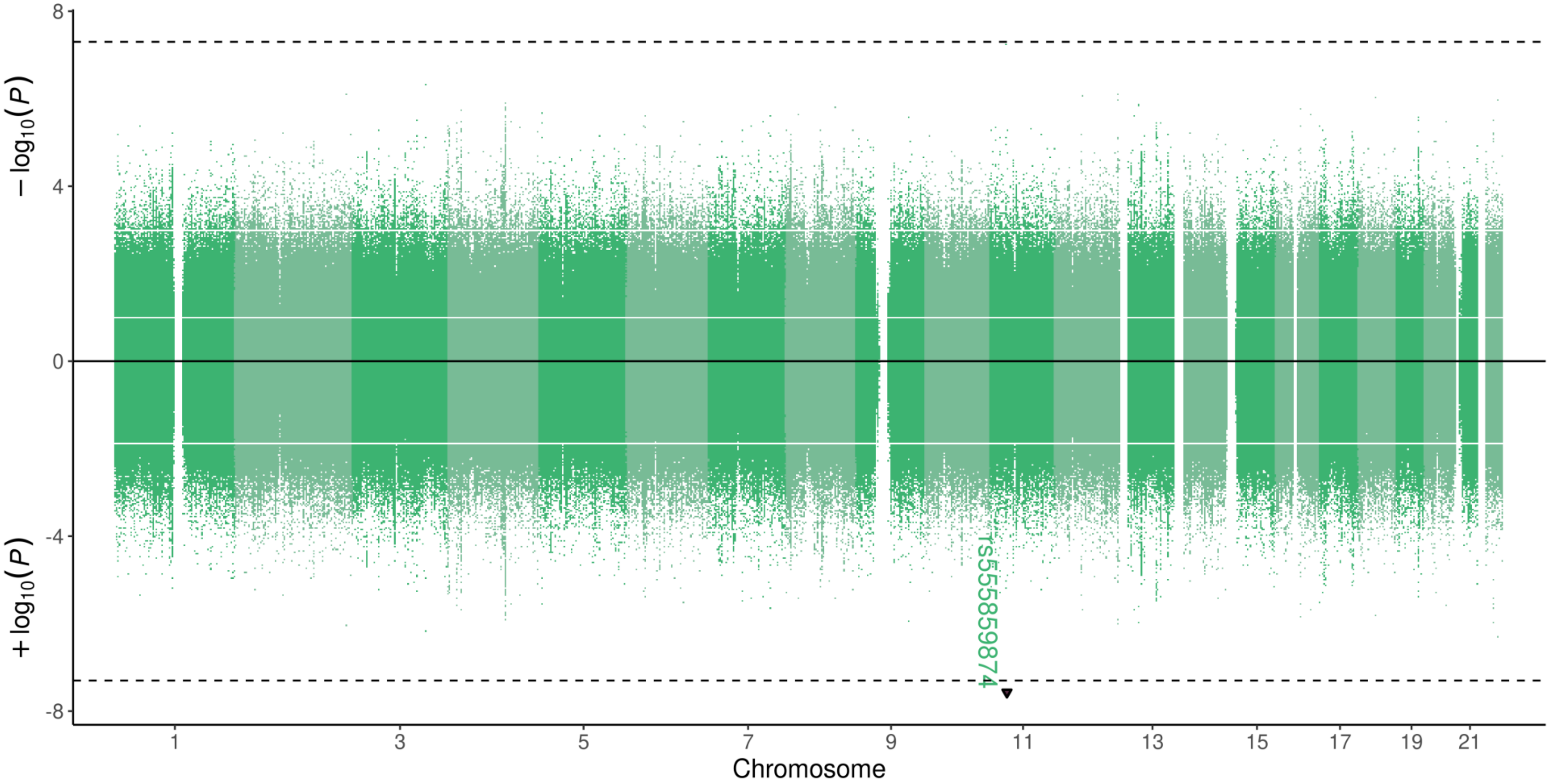

Supplementary Figure 64- Miami plot for GWAS of default-mode network with (bottom) and without (top) adjustment for RSFA

Frontoparietal network

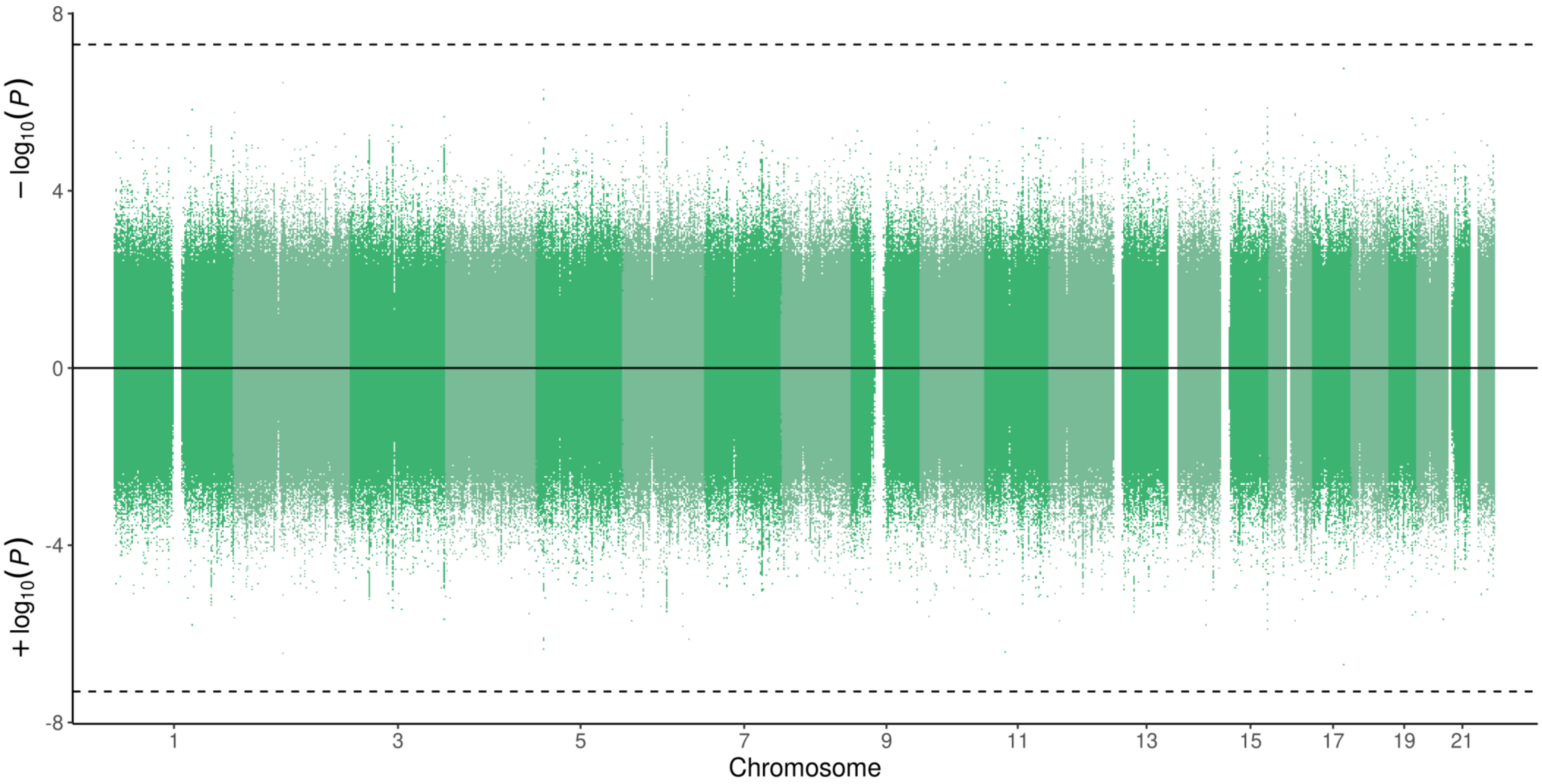

Supplementary Figure 65- Miami plot for GWAS of the frontoparietal network with (bottom) and without (top) adjustment for RSFA

Medial frontal network

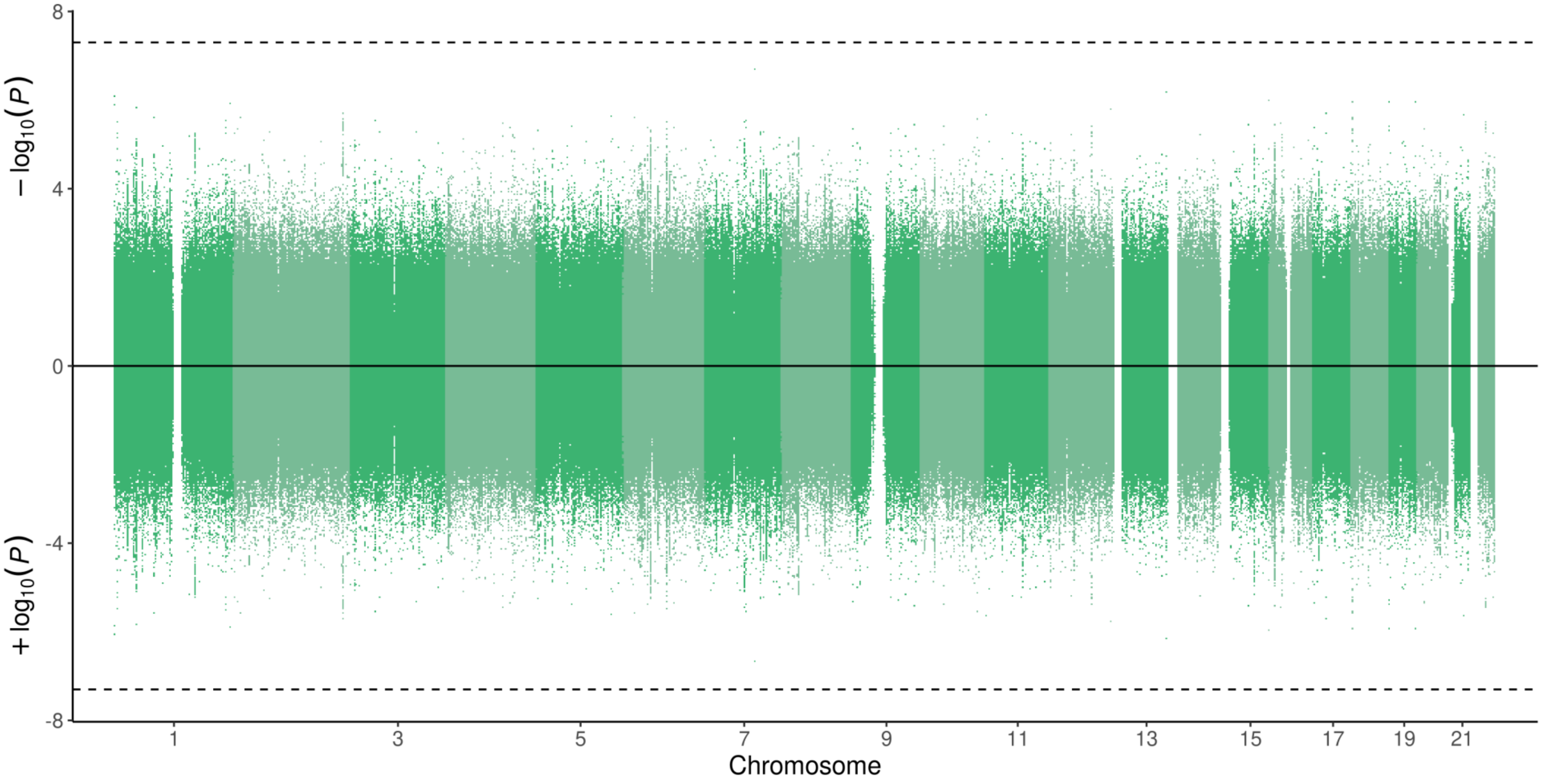

Supplementary Figure 66- Miami plot for GWAS of the medial frontal network with (bottom) and without (top) adjustment for RSFA

Motor network

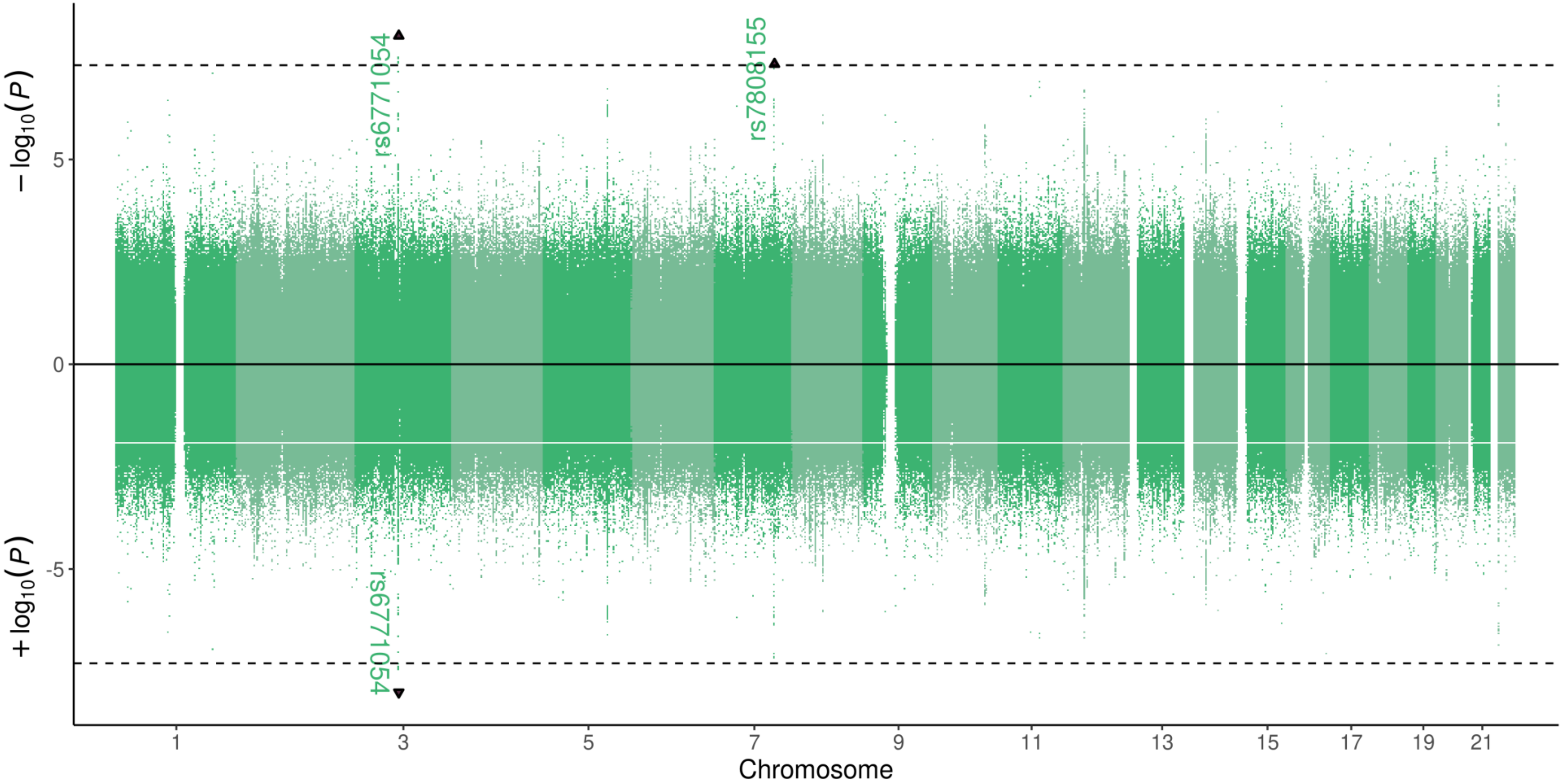

Supplementary Figure 67- Miami plot for GWAS of the motor network with (bottom) and without (top) adjustment for RSFA

Subcortical-cerebellum network

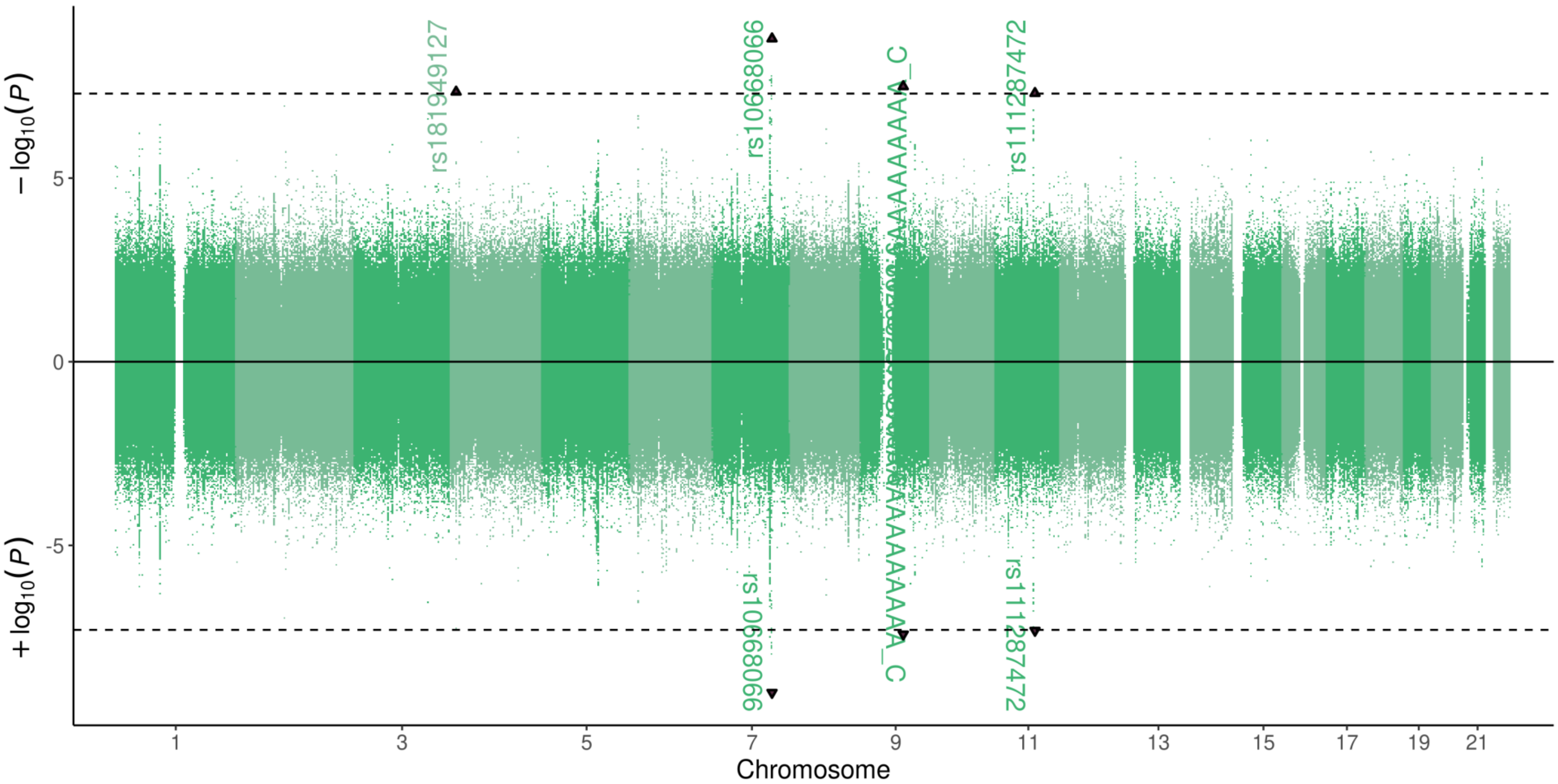

Supplementary Figure 68- Miami plot for GWAS of the subcortical-cerebellum network with (bottom) and without (top) adjustment for RSFA

Visual association network

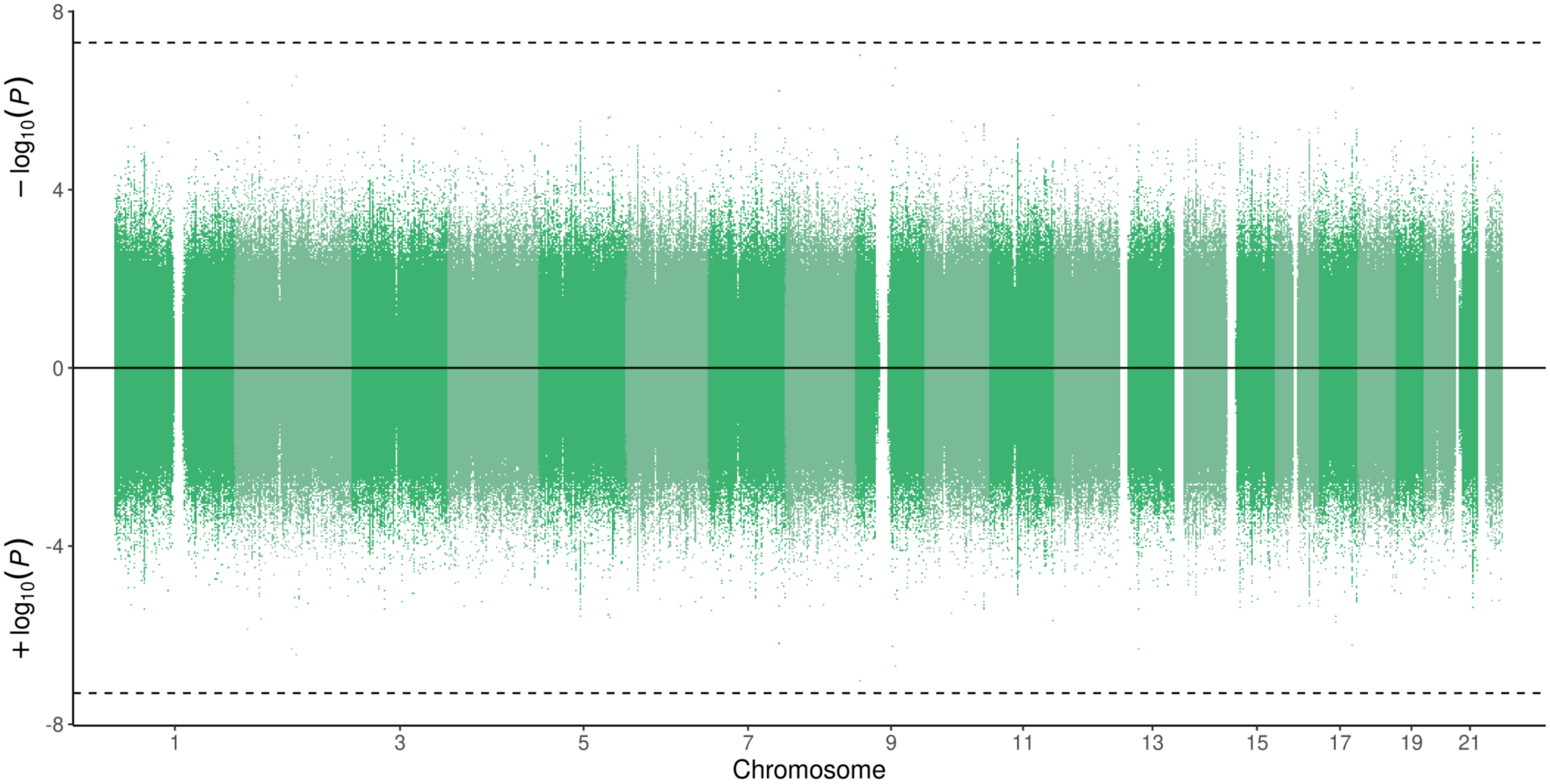

Supplementary Figure 69- Miami plot for GWAS of the visual association network with (bottom) and without (top) adjustment for RSFA

Visual network I

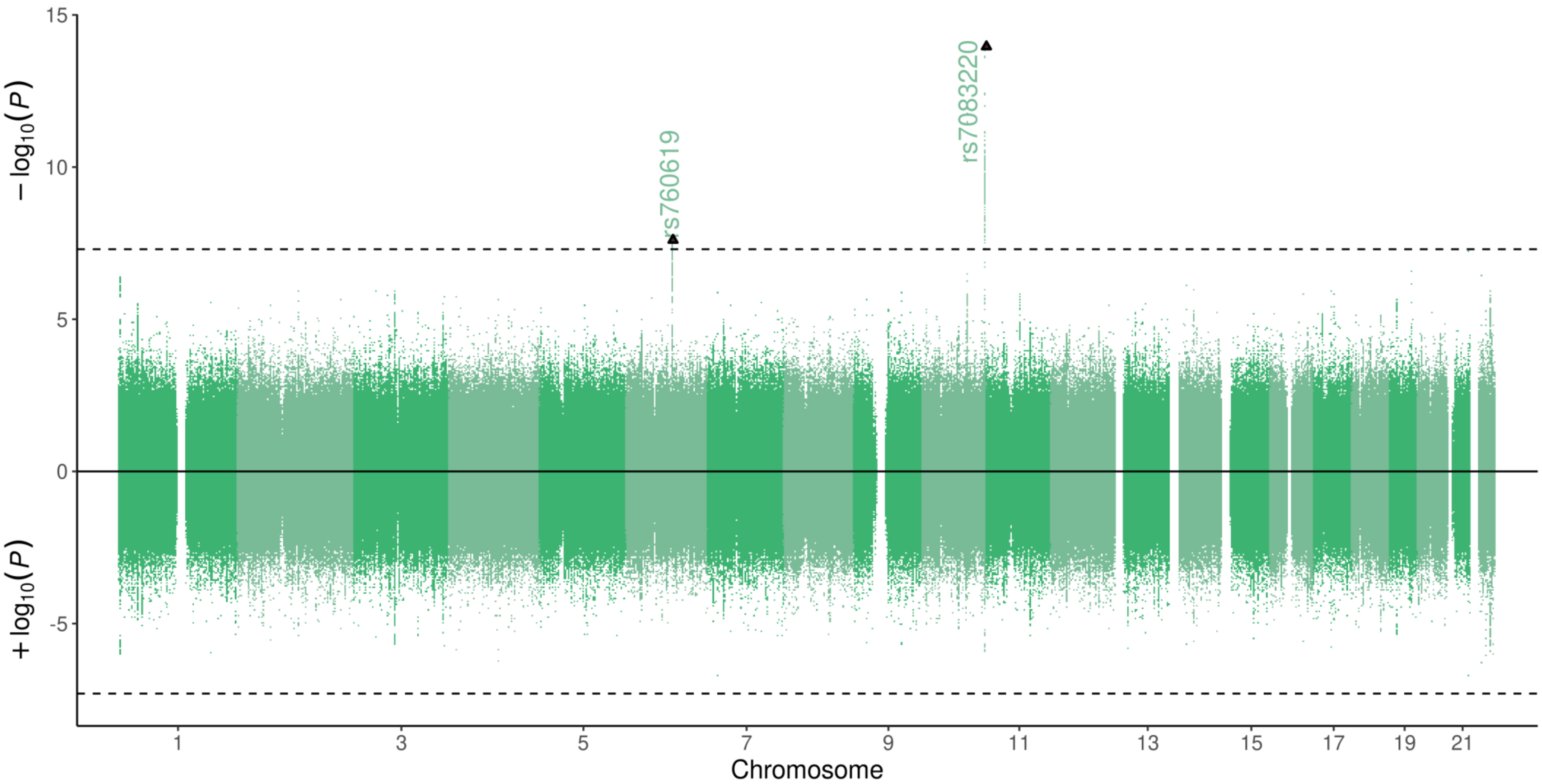

Supplementary Figure 70- Miami plot for GWAS of visual network I with (bottom) and without (top) adjustment for RSFA

Visual network 2

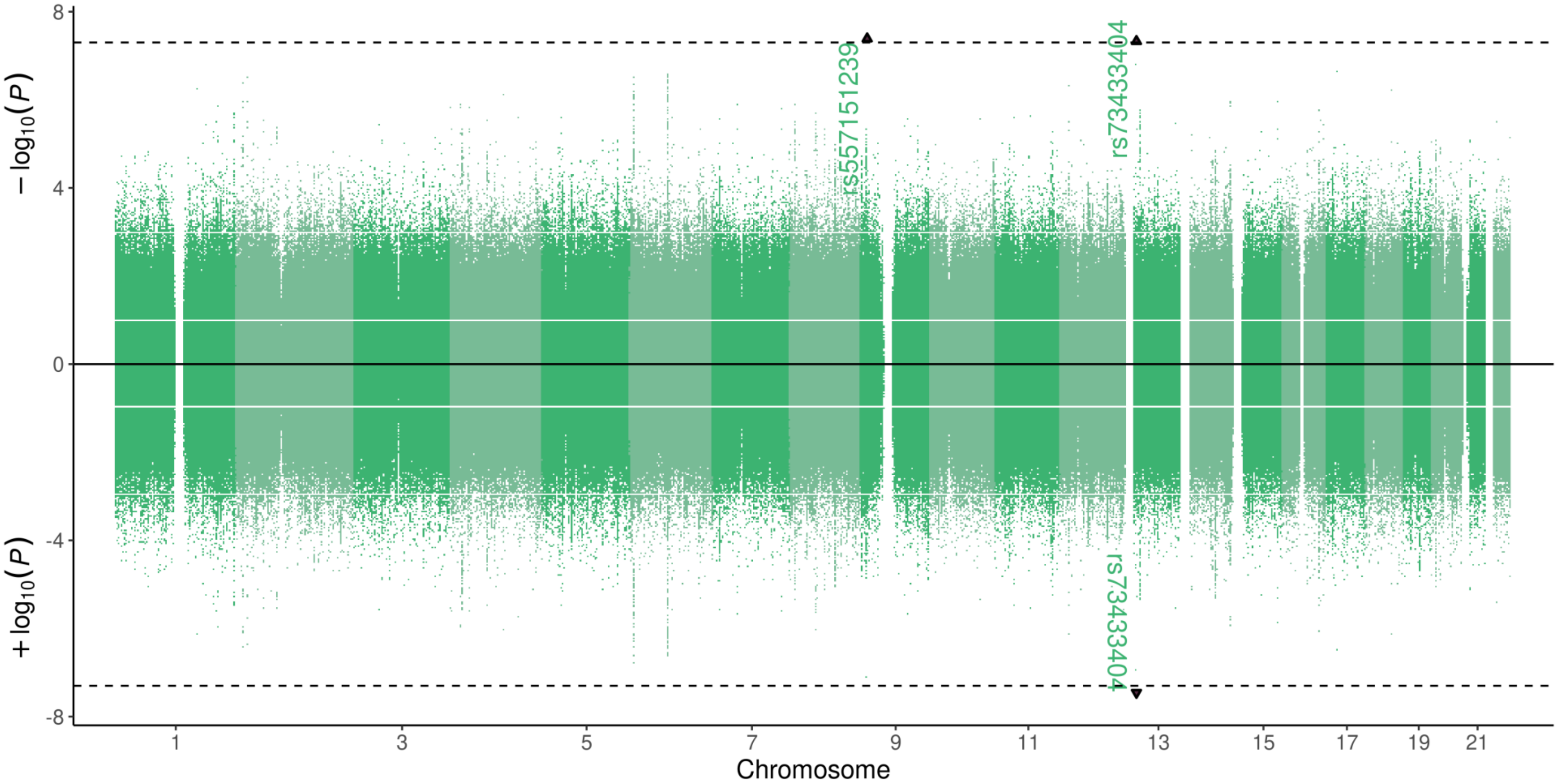

Supplementary Figure 71- Miami plot for GWAS of the visual network II with (bottom) and without (top) adjustment for RSFA
